# Health-related quality of life in patients with newly diagnosed advanced ovarian cancer receiving maintenance olaparib plus bevacizumab (PAOLA-1/ENGOT-ov25)

**DOI:** 10.1093/jnci/djag122

**Published:** 2026-04-24

**Authors:** Florence Joly, Amélie Anota, Sylvie Chabaud, Claire Cropet, Frank Priou, Philipp Harter, Sandro Pignata, Isabel Palacio, Edgar Petru, Hiroaki Kobayashi, Ignace Vergote, Gabriella Parma, Johanna Mäenpää, Eric Raymond, Beyhan Ataseven, Carmela Pisano, Nuria Lainez, Irina Tsibulak, Kan Yonemori, Peter Vuylsteke, Maria Teresa Lapresa, Mansoor Raza Mirza, Patrick Bouchaert, Paul Buderath, Domenica Lorusso, Ana Herrero, Lauriane Eberst, Alexander Burges, Giovanni Scambia, Eugenia Ortega, Cécile Guillemet, Eric Pujade-Lauraine, Jean-Emmanuel Kurtz, Isabelle Ray-Coquard

**Affiliations:** Centre François Baclesse, University Unicaen, Inserm U1086 Anticipe, Caen, France; Groupe d’Investigateurs Nationaux pour l’Etude des Cancers Ovariens (GINECO), France; Centre Léon Bérard, Lyon, France; Centre Léon Bérard, Lyon, France; Centre Léon Bérard, Lyon, France; Groupe d’Investigateurs Nationaux pour l’Etude des Cancers Ovariens (GINECO), France; CHD Les Oudairies, La Roche Sur Yon, France; Ev. Kliniken Essen-Mitte, Essen, Germany; Philipps University, Marburg, Germany; Arbeitsgemeinschaft Gynäkologische Onkologie (AGO) Studiengruppe, Germany; Istituto Nazionale Tumori IRCCS, ‘Fondazione G Pascale’, Naples, Italy; Multicenter Italian Trials in Ovarian Cancer and Gynecologic Malignancies (MITO), Italy; Hospital Universitario Central de Asturias, Oviedo, Spain; Grupo Español de Investigación en Cáncer de Ovario (GEICO), Spain; Medical University Graz, Graz, Austria; Arbeitsgemeinschaft Gynaekologische Onkologie (AGO-Austria), Austria; Kagoshima University Hospital, Kagoshima, Japan; Gynecologic Oncology Trial and Investigation Consortium (GOTIC), Japan; University Hospital Leuven, Leuven Cancer Institute, Leuven, Belgium, European Union; Belgium and Luxembourg Gynaecological Oncology Group (BGOG), Belgium, European Union; European Institute of Oncology, Milan, Italy; Mario Negri Gynecologic Oncology Group (MaNGO), Italy; Tampere University and University Hospital, Tampere, Finland; Nordic Society of Gynecologic Oncology (NSGO), Finland; Groupe d’Investigateurs Nationaux pour l’Etude des Cancers Ovariens (GINECO), France; Groupe Hospitalier Saint-Joseph, Paris, France; Ev. Kliniken Essen-Mitte, Essen, Germany; Arbeitsgemeinschaft Gynäkologische Onkologie (AGO) Studiengruppe, Germany; Istituto Nazionale Tumori IRCCS, ‘Fondazione G Pascale’, Naples, Italy; Multicenter Italian Trials in Ovarian Cancer and Gynecologic Malignancies (MITO), Italy; Grupo Español de Investigación en Cáncer de Ovario (GEICO), Spain; Hospital Universitario de Navarra, Pamplona, Spain; Arbeitsgemeinschaft Gynaekologische Onkologie (AGO-Austria), Austria; Medical University Innsbruck, Innsbruck, Austria; Gynecologic Oncology Trial and Investigation Consortium (GOTIC), Japan; National Cancer Center Hospital, Tokyo, Japan; Belgium and Luxembourg Gynaecological Oncology Group (BGOG), Belgium, European Union; CHU UCL Namur, Namur, Belgium, European Union; CHU Brugmann, Brussels, Belgium, European Union; Mario Negri Gynecologic Oncology Group (MaNGO), Italy; European Institute of Oncology, Milan, Italy; Rigshospitalet, Copenhagen University Hospital, Copenhagen, Denmark; Groupe d’Investigateurs Nationaux pour l’Etude des Cancers Ovariens (GINECO), France; Hôpital de la Milétrie, Centre Hospitalier Universitaire de Poitiers, Pôle Régional de Cancérologie, Poitiers, France; Arbeitsgemeinschaft Gynäkologische Onkologie (AGO) Studiengruppe, Germany; West German Cancer Center, University Hospital Essen, Universitätsklinikum Essen, Essen, Germany; Multicenter Italian Trials in Ovarian Cancer and Gynecologic Malignancies (MITO), Italy; Humanitas San Pio X and Humanitas University, Milan, Italy; Grupo Español de Investigación en Cáncer de Ovario (GEICO), Spain; Hospital Universitario Miguel Servet, Zaragoza, Spain; Groupe d’Investigateurs Nationaux pour l’Etude des Cancers Ovariens (GINECO), France; ICANS (Institut de Cancérologie Strasbourg Europe), Strasbourg, France; Arbeitsgemeinschaft Gynäkologische Onkologie (AGO) Studiengruppe, Germany; University Hospital, LMU Munich, Munich, Germany; Multicenter Italian Trials in Ovarian Cancer and Gynecologic Malignancies (MITO), Italy; Fondazione Policlinico Universitario A. Gemelli IRCCS and Università Cattolica del Sacro Cuore di Roma, Rome, Italy; Grupo Español de Investigación en Cáncer de Ovario (GEICO), Spain; Hospital Universitario Miguel Servet, Zaragoza, Spain; Groupe d’Investigateurs Nationaux pour l’Etude des Cancers Ovariens (GINECO), France; Center Henri Becquerel, Rouen, France; ARCAGY Research, Paris, France; Groupe d’Investigateurs Nationaux pour l’Etude des Cancers Ovariens (GINECO), France; ICANS (Institut de Cancérologie Strasbourg Europe), Strasbourg, France; Groupe d’Investigateurs Nationaux pour l’Etude des Cancers Ovariens (GINECO), France; Centre Léon Bérard, University Claude Bernard Lyon I, Lyon, France

## Abstract

**Background:**

We analyzed health-related quality of life (HRQoL) and time until definitive HRQoL deterioration for patients with newly diagnosed advanced ovarian cancer receiving olaparib plus bevacizumab or placebo plus bevacizumab in PAOLA-1.

**Methods:**

Health-related QoL and time until definitive HRQoL deterioration, prespecified secondary endpoints, were assessed by European Organisation for Research and Treatment of Cancer Core Quality of Life Questionnaire (QLQ-C30) and Ovarian Cancer module (QLQ-OV28) at baseline and then every 12 weeks for 2 years. Health-related QoL and time until definitive HRQoL deterioration by homologous recombination deficiency status and effect of progression on HRQoL were post hoc analyses.

**Results:**

A total of 806 patients were randomly assigned (olaparib plus bevacizumab *n* = 537; placebo plus bevacizumab *n* = 269). There were no clinically meaningful between-group differences in adjusted mean global change from baseline in QLQ-C30 or QLQ-OV28 domains overall (between-group difference in QLQ-C30 Global Heath Status [GHS] score = 1.65, 95% CI = −0.27 to 3.56) or in the homologous recombination deficiency–positive subgroup (between-group difference in QLQ-C30 GHS = 1.23, 95% CI = −1.25 to 3.71). Time until definitive HRQoL deterioration estimates of QLQ-C30 GHS scores did not differ between treatment arms in the modified intention-to-treat population (hazard ratio [HR] = 0.88, 95% CI = 0.72 to 1.07) and favored olaparib plus bevacizumab vs placebo plus bevacizumab in the homologous recombination deficiency–positive subgroup (HR = 0.70, 95% CI = 0.52 to 0.93). Analyses of patients (103 of 465 [22.2%]) following disease progression showed clinically meaningful deterioration in QLQ-C30 emotional and social scores.

**Conclusion:**

Adding maintenance olaparib to bevacizumab showed no clinically meaningful detrimental effect on global HRQoL either overall or in the homologous recombination deficiency–positive subgroup.

**ClinicalTrials.gov ID:**

NCT02477644.

## Introduction

A key goal of ovarian cancer maintenance therapy with poly(adenosine diphosphate [ADP]-ribose) polymerase inhibitors is to improve progression-free survival (PFS) and overall survival while maintaining health-related quality of life (HRQoL). Patients with advanced ovarian cancer in clinical response after first-line treatment frequently experience few or no disease-related symptoms, and the benefits of delaying progression must be balanced against treatment-related adverse events, which may negatively impact HRQoL.^1^ Few poly(ADP-ribose) polymerase inhibitor trials have assessed HRQoL beyond progression.^1^^,^^2^

The phase III PAOLA-1/ENGOT-ov25 trial showed statistically significant PFS benefit with the addition of maintenance olaparib to bevacizumab in patients with newly diagnosed advanced ovarian cancer.^3^ Substantial PFS benefit was seen in patients whose tumors tested positive for homologous recombination deficiency (defined as the presence of a *BRCA1* and/or *BRCA2* [BRCA] mutation and/or genomic instability), with a median PFS of 37.2 months with olaparib plus bevacizumab vs 17.7 months with placebo plus bevacizumab (hazard ratio [HR] = 0.33, 95% CI = 0.25 to 0.45).^3^ These results led to the approval of maintenance olaparib plus bevacizumab in patients with homologous recombination deficiency–positive advanced ovarian cancer in response to first-line platinum-based chemotherapy plus bevacizumab.^4^^,^^5^ Olaparib plus bevacizumab was also associated with a clinically meaningful improvement in overall survival in patients with homologous recombination deficiency–positive tumors, with 5-year overall survival rates of 65.5% vs 48.4% in the olaparib plus bevacizumab and placebo plus bevacizumab arms, respectively.^6^

As previously reported, global HRQoL, assessed via the adjusted mean change from baseline of the European Organisation for Research and Treatment of Cancer (EORTC) Core Quality of Life Questionnaire (QLQ-C30) score, was not affected to a clinically significant extent after the addition of placebo or olaparib to bevacizumab maintenance therapy in PAOLA-1.^3^

Here, we report detailed HRQoL analyses in the intention-to-treat (ITT) population and by homologous recombination deficiency status, time until definitive HRQoL deterioration, and post hoc exploratory analyses of HRQoL following disease progression and time without clinically significant symptoms or toxicity.

## Methods

### Study design and patients

The design of the PAOLA-1 phase III randomized, double-blind, placebo-controlled, multicenter trial (NCT024776447) has been previously reported.^3^ The protocol was approved by ethics committees at each participating site, and the trial was performed in accordance with the ethical principles of the Declaration of Helsinki and Good Clinical Practice guidelines. All patients provided written informed consent.

Eligible patients had newly diagnosed advanced (International Federation of Gynecology and Obstetrics stage III or IV) high-grade serous or high-grade endometrioid ovarian, primary peritoneal, and/or fallopian tube cancer; other epithelial nonmucinous ovarian cancers were permitted if patients had a deleterious germline BRCA mutation. Patients were eligible irrespective of biomarker or surgical status. Patients had no evidence of disease or were in clinical complete or partial response following surgery and platinum-based chemotherapy plus bevacizumab (Supplementary Methods).

The MyChoice HRD Plus assay (Myriad Genetic Laboratories, Inc, Salt Lake City, UT, USA) was used retrospectively to determine the prespecified tumor homologous recombination deficiency status of patients (a positive test was defined as a tumor BRCA mutation and/or genomic instability score ≥42) before the primary analysis. Before trial entry, tumor BRCA mutation status was determined by central French academic laboratories.

### Study treatment

Patients were randomly assigned 2:1 to olaparib tablets (300 mg twice daily) or placebo 3-9 weeks after their last chemotherapy dose. Random assignment was stratified by first-line treatment outcome at screening and tumor BRCA mutation status. Maintenance therapy continued for up to 24 months or until investigator-assessed radiological disease progression (according to modified RECIST v1.1), unacceptable toxicity, or other discontinuation criteria were met. All patients received bevacizumab 15 mg/kg every 3 weeks for up to 15 months (including alongside platinum-based chemotherapy).

### Study endpoints

The primary endpoint of PFS (data cutoff March 22, 2019)^3^ and the key secondary endpoint of overall survival (data cutoff March 22, 2022)^6^ have been reported previously. Prespecified secondary endpoints included HRQoL, reported as mean change in Global Health Status (GHS) score and functional and symptomatic scale scores over time using the EORTC QLQ-C30^8^ and EORTC Quality of Life Questionnaire Ovarian Cancer module (QLQ-OV28),^9^ and time until definitive HRQoL deterioration (defined as the time interval between random assignment and the occurrence of the first clinically relevant deterioration in QLQ-C30 and QLQ-OV28 scores from baseline with no further clinically relevant improvement from baseline, or all-cause death). Health-related QoL and time until definitive HRQoL deterioration by homologous recombination deficiency status, HRQoL pre- vs postprogression, and time without clinically significant symptoms or toxicity were post hoc exploratory analyses.

### Study assessments

EORTC QLQ-C30,^8^ a validated 30-question instrument specific to patients with cancer, includes a GHS scale, 5 functional scales (physical, role, emotional, cognitive, and social functioning), and 9 symptomatic scales (fatigue, nausea and vomiting, pain, dyspnea, insomnia, appetite loss, constipation, diarrhea, and financial difficulties). EORTC QLQ-OV28^9^ is a validated ovarian cancer–specific 28-question instrument designed for use in patients with local or advanced disease who undergo surgery with or without chemotherapy. It includes 3 functional scales (body image, sexual function, and attitude to disease and treatment) and 5 symptomatic scales (abdominal and gastrointestinal symptoms, peripheral neuropathy, hormonal and menopausal symptoms, other chemotherapy side effects, and hair loss). For QLQ-C30 and QLQ-OV28, scores range from 0 to 100, with higher scores for GHS and functional scales representing higher global QoL and higher functional level, respectively, and higher scores for symptomatic scales representing higher symptomatic burden.

Patients self-completed questionnaires (on paper) in their native language at baseline and every 12 weeks (±6 weeks) for 2 years following random assignment during clinic visits, irrespective of whether they experienced disease progression. Only the questionnaire closest to the planned measurement timepoint was used if more than 1 questionnaire was available. Questionnaires were no longer completed once patients discontinued the study, completed 2 years of follow-up, or died.

### Statistical analyses

The ITT population included all randomly assigned patients regardless of intervention received. Health-related QoL analyses were performed on patients who completed a baseline and at least 1 postbaseline questionnaire (modified ITT population).

Health-related QoL data were analyzed in the modified ITT population and in homologous recombination deficiency–positive and homologous recombination deficiency–negative subgroups. All questionnaires completed during 2 years of follow-up were included in the HRQoL analysis, even if the patient had progressed. Each scale of QLQ-C30 and QLQ-OV28 was analyzed independently. Adjusted mean change from each HRQoL score was analyzed by mixed model for repeated measures until 2 years of follow-up. The Kaplan–Meier method was used to generate time-to-event curves for time until definitive HRQoL deterioration with a univariate Cox model used to estimate hazard ratios (HRs) and 95% confidence intervals. The prespecified minimal important difference for clinically relevant change in any HRQoL scale and time until definitive HRQoL deterioration was fixed at 10 points.^10^ All *P* values less than .05 were considered statistically significant.

The HRQoL postprogression analysis was conducted on all available HRQoL data for both treatment arms combined. EORTC QLQ-C30 scores following diagnosis of RECIST progression (up to a maximum of 60 days after progression) were compared with the last measurement collected prior to progression (up to a maximum of 60 days before progression). Results were expressed as mean change in HRQoL score between pre- and postprogression with 95% confidence interval.

Time without clinically significant symptoms or toxicity was analyzed in the ITT population and by homologous recombination deficiency status (data cutoff March 22, 2019) and calculated as the total number of days without symptoms or toxicity between random assignment and first disease progression or censoring for progression (PFS minus toxicity) for 3 toxicity states (see Supplementary Methods and Figure S1).

Detailed methodology is provided in the Supplementary Methods.

## Results

### Patient disposition

A total of 806 eligible patients were randomly assigned to olaparib plus bevacizumab (*n* = 537) or placebo plus bevacizumab (*n* = 269) (ITT population), and 535 and 267 patients, respectively, received investigational treatment (Figure 1). A total of 770 patients (517 in the olaparib plus bevacizumab arm and 253 in the placebo plus bevacizumab arm) had a baseline and at least 1 follow-up score and constitute the modified ITT population.

**Figure 1.**
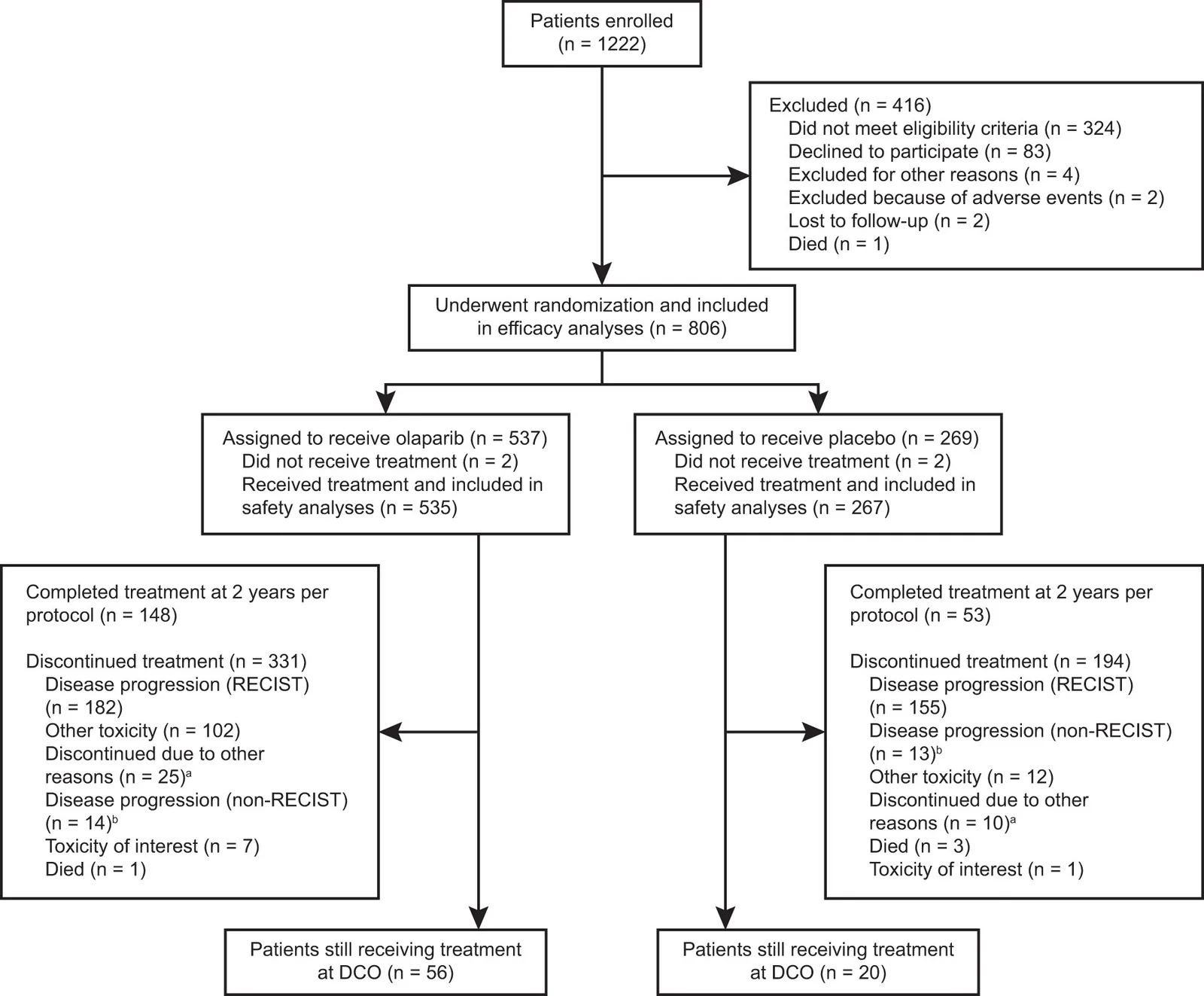
CONSORT diagram (data cutoff [DCO] March 22, 2019). ^a^Other reasons included consent withdrawn (*n* = 4, olaparib plus bevacizumab; *n* = 4, placebo plus bevacizumab), lost to follow-up (*n* = 1, olaparib plus bevacizumab), missing (*n* = 1, olaparib plus bevacizumab), and other (*n* = 19, olaparib plus bevacizumab; *n* = 6, placebo plus bevacizumab). ^b^Disease progression defined by criteria other than RECIST (*n* = 9, olaparib plus bevacizumab; *n* = 10, placebo plus bevacizumab) or symptomatic deterioration (*n* = 5, olaparib plus bevacizumab; *n* = 3, placebo plus bevacizumab).

Baseline characteristics were well balanced between treatment arms in the ITT population and homologous recombination deficiency–positive subgroup (Table 1).^3^ Baseline QLQ-C30 GHS scores were also similar between treatment arms (Table 1).

**Table 1.** Baseline characteristics in the ITT population and the HRD-positive subgroup.^a^

Baseline characteristic	ITT population		HRD-positive subgroup	
	Olaparib plus bevacizumab (*n* = 537)	Placebo plus bevacizumab (*n* = 269)	Olaparib plus bevacizumab (*n* = 255)	Placebo plus bevacizumab (*n* = 132)
Age, years, median (range)	61.0 (32.0-87.0)	60.0 (26.0-85.0)	58.0 (32.0-77.0)	58.0 (35.0-82.0)
Eastern Cooperative Oncology Group performance status, No. (%)				
0	378 (70)	189 (70)	190 (75)	100 (76)
1	153 (28)	76 (28)	61 (24)	31 (23)
Missing	6 (1)	4 (1)	4 (2)	1 (1)
Primary tumor location, No. (%)				
Ovary	456 (85)	238 (88)	217 (85)	118 (89)
Fallopian tubes	39 (7)	11 (4)	24 (9)	5 (4)
Primary peritoneal	42 (8)	20 (7)	14 (5)	9 (7)
International Federation of Gynecology and Obstetrics stage, No. (%)				
III	378 (70)	186 (69)	182 (71)	90 (68)
IV	159 (30)	83 (31)	73 (29)	42 (32)
Histology, No. (%)				
Serous	519 (97)	253 (94)	242 (95)	124 (94)
Endometrioid	12 (2)	8 (3)	9 (4)	4 (3)
Other	6 (1)	8 (3)	4 (2)	4 (3)
History of cytoreductive surgery, No. (%)				
Upfront surgery	271 (50)	138 (51)	145 (57)	79 (60)
Macroscopic residual disease	111 (41)	53 (38)	55 (38)	30 (38)
Complete resection	160 (59)	85 (62)	90 (62)	49 (62)
Interval surgery	228 (42)	110 (41)	100 (39)	45 (34)
Macroscopic residual disease	65 (29)	35 (32)	24 (24)	13 (29)
Complete resection	163 (71)	75 (68)	76 (76)	32 (71)
No surgery	38 (7)	21 (8)	10 (4)	8 (6)
Response after first-line therapy, No. (%)				
No evidence of disease^b^	290 (54)	141 (52)	150 (59)	71 (54)
Clinical complete response^c^	106 (20)	53 (20)	52 (20)	26 (20)
Clinical partial response^d^	141 (26)	75 (28)	53 (21)	35 (27)
Deleterious tumor BRCA mutation,^e^ No. (%)				
Yes	157 (29)	80 (30)	150 (59)	65 (49)
No	380 (71)	189 (70)	105 (41)	67 (51)
Tumor HRD status,^f^ No. (%)				
HRD-positive	255 (47)	132 (49)	255 (100)	132 (100)
HRD-negative or unknown	282 (53)	137 (51)	0	0
HRD-negative	192 (36)	85 (32)	0	0
Unknown	90 (17)	52 (19)	0	0

Abbreviations: CA-125 = cancer antigen 125; HRD = homologous recombination deficiency; ITT = intention-to-treat.

a Percentages may not total 100 because of rounding.

b Defined as no measurable or assessable disease after cytoreductive surgery, in addition to no radiologic evidence of disease and a normal CA-125 level after chemotherapy.

c Defined as the disappearance of all measurable or assessable disease and normalization of CA-125 levels following chemotherapy.

d Defined as radiologic evidence of disease, an abnormal CA-125 level, or both.

e Per the electronic case report form.

f Homologous recombination deficiency–positive was defined as a tumor BRCA mutation and/or genomic instability score of at least 42 on the MyChoice CDx assay (Myriad Genetic Laboratories, Inc., Salt Lake City, UT, USA). Homologous recombination deficiency–negative was defined as a genomic instability score less than 42. Unknown was defined as an inconclusive, missing, or failed MyChoice CDx assay.

### Treatment exposure

Among the patients who received investigational treatment, the median duration of treatment for olaparib was 17.3 months (range = 0.0-33.0 months) and for placebo was 15.6 months (range = 0.1-26.2 months) in the olaparib plus bevacizumab and placebo plus bevacizumab arms, respectively. The median duration of treatment for bevacizumab since random assignment was 11.0 months (range = 0.7-21.4 months) and 10.6 months (range = 0.7-17.1 months), respectively (data cutoff March 22, 2019).

### EORTC QLQ-C30 and QLQ-OV28 compliance and completion rates

In the ITT population, baseline QLQ-C30 compliance rates were high and well balanced between treatment arms (96.1% [516 of 537] and 94.1% [253 of 269] with olaparib plus bevacizumab and placebo plus bevacizumab, respectively) (Table S1). Compliance rates remained high through week 96 (72.1% [302 of 419] and 71.0% [147 of 207], respectively), with little difference between treatment arms at any timepoint (Table S1). Similar compliance rates were reported for the QLQ-OV28 (Table S2). Completion rates are reported in Tables S1 and S2; “site missed administration” was generally the most common reason for questionnaires not being completed (Table S3).

### Adjusted mean change from baseline in HRQoL

In the modified ITT population, the adjusted mean global change from baseline in QLQ-C30 GHS score was −1.12 (95% CI = −2.21 to −0.02) and −2.76 (95% CI = −4.34 to −1.19) with olaparib plus bevacizumab and placebo plus bevacizumab, respectively (difference between arms = 1.65, 95% CI = −0.27 to 3.56) (Table 2). Because the time-by-treatment interaction for this dimension was statistically significant (*P* = .0015), results by timepoint should also be considered (Table S4). The mean change from baseline in GHS remained close to zero through week 96 in both treatment arms (Figure 2A).

**Figure 2.**
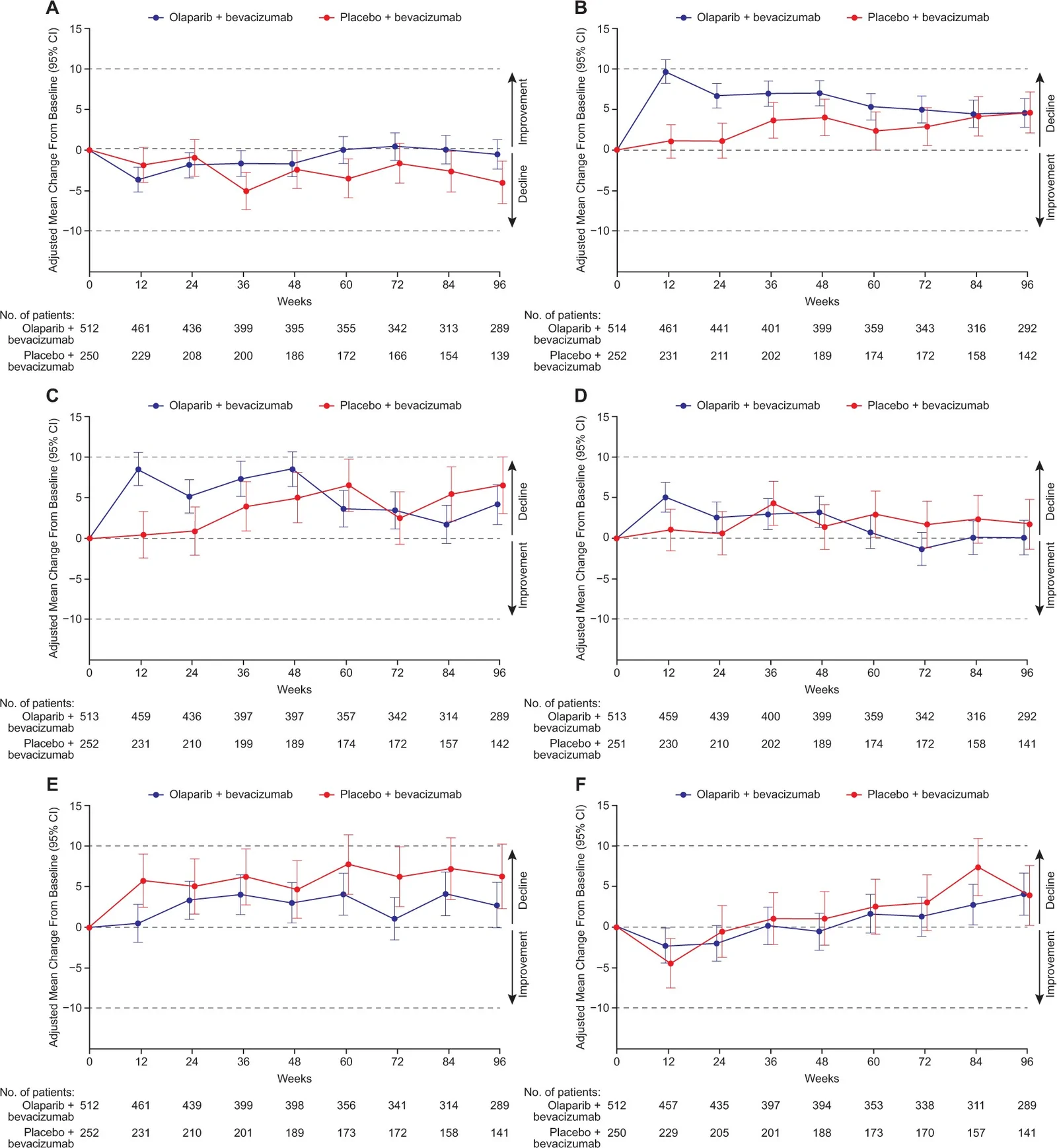
Adjusted mean change over time in European Organisation for Research and Treatment of Cancer Core Quality of Life Questionnaire scores for **(A)** Global Health Status, **(B)** nausea and vomiting, **(C)** appetite loss, **(D)** fatigue, **(E)** insomnia, and **(F)** constipation in the modified intention-to-treat population. Adjusted mean change using mixed model for repeated measures at each follow-up timepoint; error bars represent 95% confidence interval. **Horizontal dotted lines** represent 10-point minimal important difference. For Global Health Status, higher scores represent higher global quality of life. For symptomatic scales, higher scores represent higher symptomatic burden.

**Table 2.** Baseline European Organisation for Research and Treatment of Cancer Core Quality of Life Questionnaire scores and adjusted mean global change from baseline in the modified intention-to-treat population.^a^

Scale scores	Olaparib plus bevacizumab (*n* = 517)	Placebo plus bevacizumab (*n* = 253)	Between-group difference	Treatment-by-time interaction, *P*
Functional scales				
*Global Health Status*				
Baseline	*n* = 512	*n* = 250		
Mean (SD)	68.6 (18.0)	67.2 (16.2)		
Median (range)	66.7 (0-100)	66.7 (25-100)		
Adjusted mean global change from baseline (95% CI)	−1.12 (−2.21 to −0.02)	−2.76 (−4.34 to −1.19)	1.65 (−0.27 to 3.56)	.0015
*Physical functioning*				
Baseline	*n* = 513	*n* = 252		
Mean (SD)	78.8 (17.6)	75.7 (19.1)		
Median (range)	80.0 (20-100)	80.0 (20-100)		
Adjusted mean global change from baseline (95% CI)	0.48 (−0.56 to 1.53)	0.38 (−1.11 to 1.87)	0.11 (−1.72 to 1.93)	.1318
* Role functioning*				
Baseline	*n* = 514	*n* = 252		
Mean (SD)	73.4 (27.2)	72.2 (27.0)		
Median (range)	83.3 (0-100)	66.7 (0-100)		
Adjusted mean global change from baseline (95% CI)	0.46 (−1.00 to 1.93)	−1.33 (−3.42 to 0.76)	1.79 (−0.76 to 4.35)	.0740
* Emotional functioning*				
Baseline	*n* = 515	*n* = 251		
Mean (SD)	76.9 (21.5)	77.2 (19.7)		
Median (range)	83.3 (0-100)	83.3 (0-100)		
Adjusted mean global change from baseline (95% CI)	−2.98 (−4.25 to −1.71)	−4.68 (−6.50 to −2.85)	1.69 (−0.53 to 3.92)	.4289
* Cognitive functioning*				
Baseline	*n* = 515	*n* = 251		
Mean (SD)	82.6 (20.9)	81.4 (19.5)		
Median (range)	83.3 (0-100)	83.3 (0-100)		
Adjusted mean global change from baseline (95% CI)	−1.61 (−2.80 to −0.42)	−2.76 (−4.47 to −1.05)	1.15 (−0.93 to 3.24)	.9978
*Social functioning*				
Baseline	*n* = 515	*n* = 251		
Mean (SD)	75.0 (26.9)	74.6 (25.3)		
Median (range)	83.3 (0-100)	83.3 (0-100)		
Adjusted mean global change from baseline (95% CI)	2.44 (0.99 to 3.89)	1.80 (−0.28 to 3.88)	0.64 (−1.89 to 3.18)	.5827
Symptomatic scales				
*Fatigue*^b^				
Baseline	*n* = 513	*n* = 251		
Mean (SD)	33.7 (22.8)	34.5 (22.5)		
Median (range)	33.3 (0-100)	33.3 (0-100)		
Adjusted mean global change from baseline (95% CI)	1.61 (0.29 to 2.93)	1.93 (0.05 to 3.81)	−0.32 (−2.62 to 1.98)	.0006
*Nausea and vomiting*^b^				
Baseline	*n* = 514	*n* = 252		
Mean (SD)	4.6 (12.5)	3.6 (9.2)		
Median (range)	0.0 (0-100)	0.0 (0-50)		
Adjusted mean global change from baseline (95% CI)	6.24 (5.26 to 7.22)	3.02 (1.62 to 4.41)	3.22 (1.51 to 4.93)	<.0001
*Pain*^b^				
Baseline	*n* = 516	*n* = 253		
Mean (SD)	22.4 (24.2)	23.8 (22.9)		
Median (range)	16.7 (0-100)	16.7 (0-100)		
Adjusted mean global change from baseline (95% CI)	3.48 (1.98 to 4.98)	7.33 (5.19 to 9.47)	−3.85 (−6.47 to −1.24)	.3168
*Dyspnea*^b^				
Baseline	*n* = 509	*n* = 252		
Mean (SD)	23.1 (25.7)	23.0 (27.3)		
Median (range)	33.3 (0-100)	16.7 (0-100)		
Adjusted mean global change from baseline (95% CI)	0.25 (−1.28 to 1.78)	−0.13 (−2.30 to 2.04)	0.38 (−2.28 to 3.04)	.0796
*Insomnia*^b^				
Baseline	*n* = 512	*n* = 252		
Mean (SD)	27.0 (30.1)	24.6 (27.2)		
Median (range)	33.3 (0-100)	33.3 (0-100)		
Adjusted mean global change from baseline (95% CI)	2.81 (1.18 to 4.44)	6.10 (3.78 to 8.43)	−3.29 (−6.13 to −0.45)	.7097
*Appetite loss*^b^				
Baseline	*n* = 513	*n* = 252		
Mean (SD)	8.3 (18.2)	9.7 (20.2)		
Median (range)	0.0 (0-100)	0.0 (0-100)		
Adjusted mean global change from baseline (95% CI)	5.24 (3.88 to 6.60)	3.84 (1.89 to 5.78)	1.40 (−0.97 to 3.77)	<.0001
*Constipation*^b^				
Baseline	*n* = 512	*n* = 250		
Mean (SD)	17.8 (26.8)	17.6 (27.6)		
Median (range)	0.0 (0-100)	0.0 (0-100)		
Adjusted mean global change from baseline (95% CI)	0.61 (−0.85 to 2.06)	1.72 (−0.36 to 3.79)	−1.11 (−3.65 to 1.42)	.2833
*Diarrhea*^b^				
Baseline	*n* = 510	*n* = 252		
Mean (SD)	10.1 (20.3)	13.1 (23.6)		
Median (range)	0.0 (0-100)	0.0 (0-100)		
Adjusted mean global change from baseline (95% CI)	−0.59 (−1.77 to 0.58)	1.50 (−0.18 to 3.17)	−2.09 (−4.14 to −0.04)	.1862
*Financial difficulties*^b^				
Baseline	*n* = 508	*n* = 246		
Mean (SD)	14.6 (27.6)	16.3 (28.0)		
Median (range)	0.0 (0-100)	0.0 (0-100)		
Adjusted mean global change from baseline (95% CI)	−1.40 (−2.74 to −0.06)	−0.69 (−2.61 to 1.24)	−0.72 (−3.06 to 1.63)	.9181

Abbreviations: HRQoL = health-related quality of life.

a Longitudinal analysis of HRQoL data was conducted using a mixed model for repeated measures until 2 years of follow-up for each HRQoL scale separately. When time-by-treatment interactions are statistically significant, time-specific estimates (Table S4) may be more relevant than estimates of mean global change.

b Symptomatic scale where higher scores represent higher symptomatic burden. For all other HRQoL scales, higher scores represent higher QoL level.

EORTC QLQ-C30 scores for nausea and vomiting favored placebo plus bevacizumab vs olaparib plus bevacizumab from weeks 12 to 72 (Figure 2B). EORTC QLQ-C30 appetite loss scores favored placebo plus bevacizumab vs olaparib plus bevacizumab from weeks 12 to 48 (Figure 2C). EORTC QLQ-C30 fatigue scores initially favored placebo plus bevacizumab (week 12) but favored olaparib plus bevacizumab from week 60 onward (Figure 2D). EORTC QLQ-C30 insomnia scores favored olaparib plus bevacizumab vs placebo plus bevacizumab from weeks 12 to 96 (Figure 2E). Over time there was minimal difference between treatment arms in QLQ-C3 scores for constipation (Figure 2F). None of the differences between treatment groups exceeded the minimal important difference (Table S4).

In other dimensions of the QLQ-C30 and QLQ-OV28, none of the between-group differences with mixed model were clinically relevant at the 10-point minimal important difference (Table 2; Tables S4 and S5 and Figures S2, S3, and S4).

### Adjusted mean change from baseline in HRQoL by homologous recombination deficiency status

In the homologous recombination deficiency–positive and homologous recombination deficiency–negative subgroups, clinically relevant worsening or improvement in some QLQ-C30 and QLQ-OV28 domain scores was seen with olaparib plus bevacizumab or placebo plus bevacizumab at certain timepoints (Tables S6, S7, S8, and S9). For example, with olaparib plus bevacizumab, clinically relevant worsening of nausea or vomiting and appetite loss were observed at week 12 in the homologous recombination deficiency–positive subgroup (Table S6). However, no between-group differences in any adjusted mean QLQ-C30 or QLQ-OV28 change from baseline scores were clinically relevant at the 10-point minimal important difference, either at specific timepoints or globally in the homologous recombination deficiency–positive or homologous recombination deficiency–negative subgroups (Figures S5 and S6). In the homologous recombination deficiency–positive subgroup, the difference between the olaparib plus bevacizumab and placebo plus bevacizumab arms in the adjusted mean global change from baseline in QLQ-C30 GHS score was 1.23 (95% CI = −1.25 to 3.71) (Table S6).

### Mean change in QLQ-C30 scores postprogression

Health-related QoL questionnaires completed following progression were available for 103 of 465 (22.2%) patients who had RECIST progression during the 2 years of follow-up. For treatment arms combined, clinically meaningful deterioration in QLQ-C30 scores following progression was seen in emotional functioning (change pre- to postprogression mean = −12.30, 95% CI= −16.46 to −8.13) and social functioning (mean = −11.17, 95% CI = −16.21 to −6.12) (Figure 3). Mean changes in QLQ-C30 scores by treatment arm following progression are reported in Table S10.

**Figure 3.**
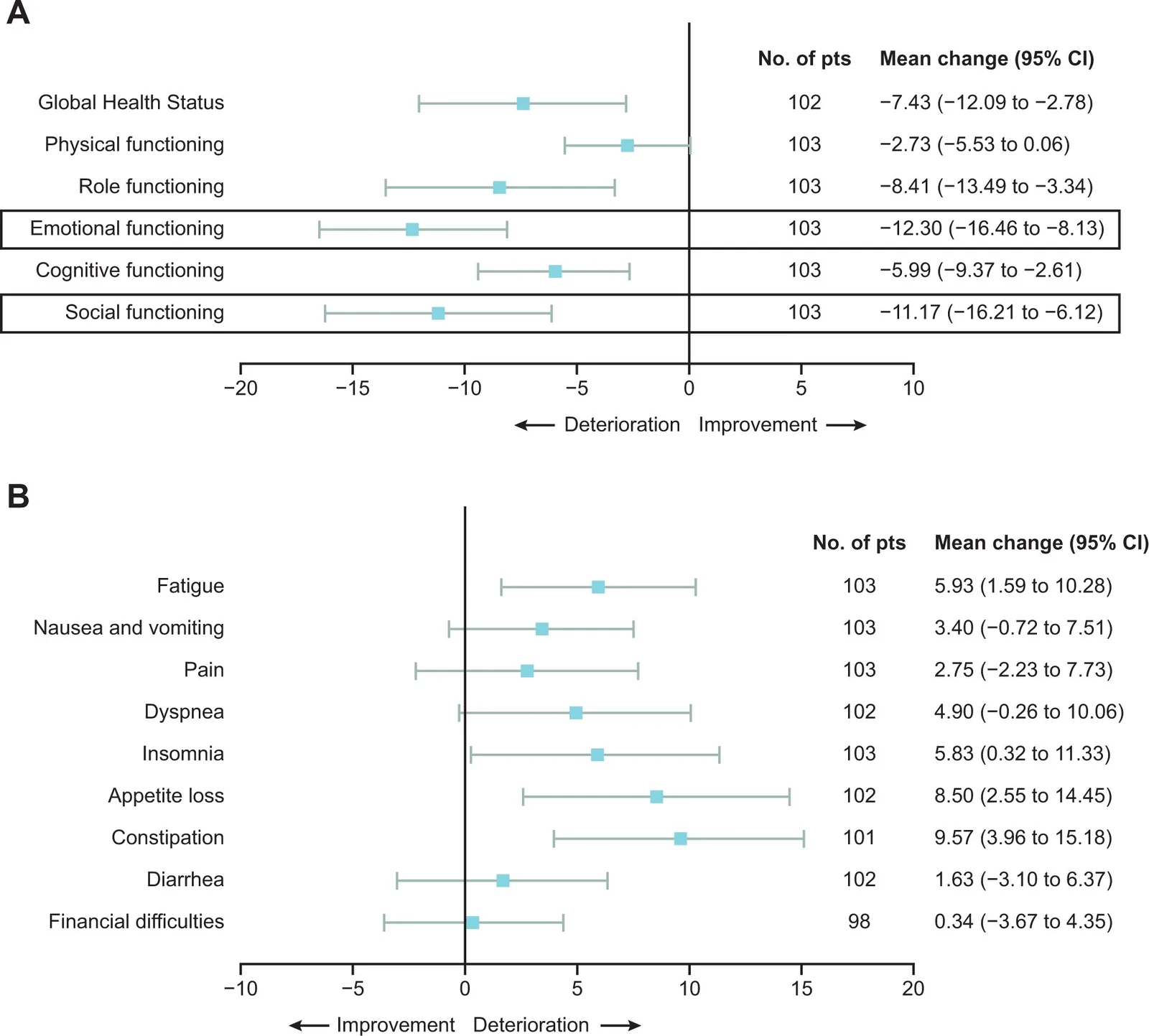
Mean change in **(A)** functional and **(B)** symptomatic EORTC QLQ-C30 scores following RECIST progression. Data are shown for olaparib plus bevacizumab and placebo plus bevacizumab treatment arms combined. EORTC QLQ-C30 scores following diagnosis of RECIST progression were compared with the last measurement collected prior to progression. Assessments were conducted up to a maximum of 60 days before and 60 days after progression. EORTC QLQ-C30 scores with clinically meaningful deterioration following RECIST progression are highlighted. Abbreviations: EORTC QLQ-C30 = European Organisation for Research and Treatment of Cancer Core Quality of Life Questionnaire; pts = patients.

### Time until definitive HRQoL deterioration

In the modified ITT population, time until definitive HRQoL deterioration estimates of QLQ-C30 GHS did not differ between treatment arms (HR = 0.88, 95% CI = 0.72 to 1.07) (Figure 4A). In the homologous recombination deficiency–positive subgroup, time until definitive HRQoL deterioration estimates of QLQ-C30 GHS favored olaparib plus bevacizumab vs placebo plus bevacizumab (HR = 0.70, 95% CI = 0.52 to 0.93) (Figure 4B). In the modified ITT population, time until definitive HRQoL deterioration estimates for each QLQ-C30 and QLQ-OV28 scale score are reported in Figure 5. In summary, apart from QLQ-C30 appetite loss (HR = 1.36, 95% CI = 1.10 to 1.67) and nausea and vomiting (HR = 1.63, 95% CI = 1.35 to 1.98) scores, which were numerically in favor of placebo plus bevacizumab vs olaparib plus bevacizumab, time until definitive HRQoL deterioration estimates for each QLQ-C30 and QLQ-OV28 scale score did not differ to a statistically significant extent between treatment arms.

**Figure 4.**
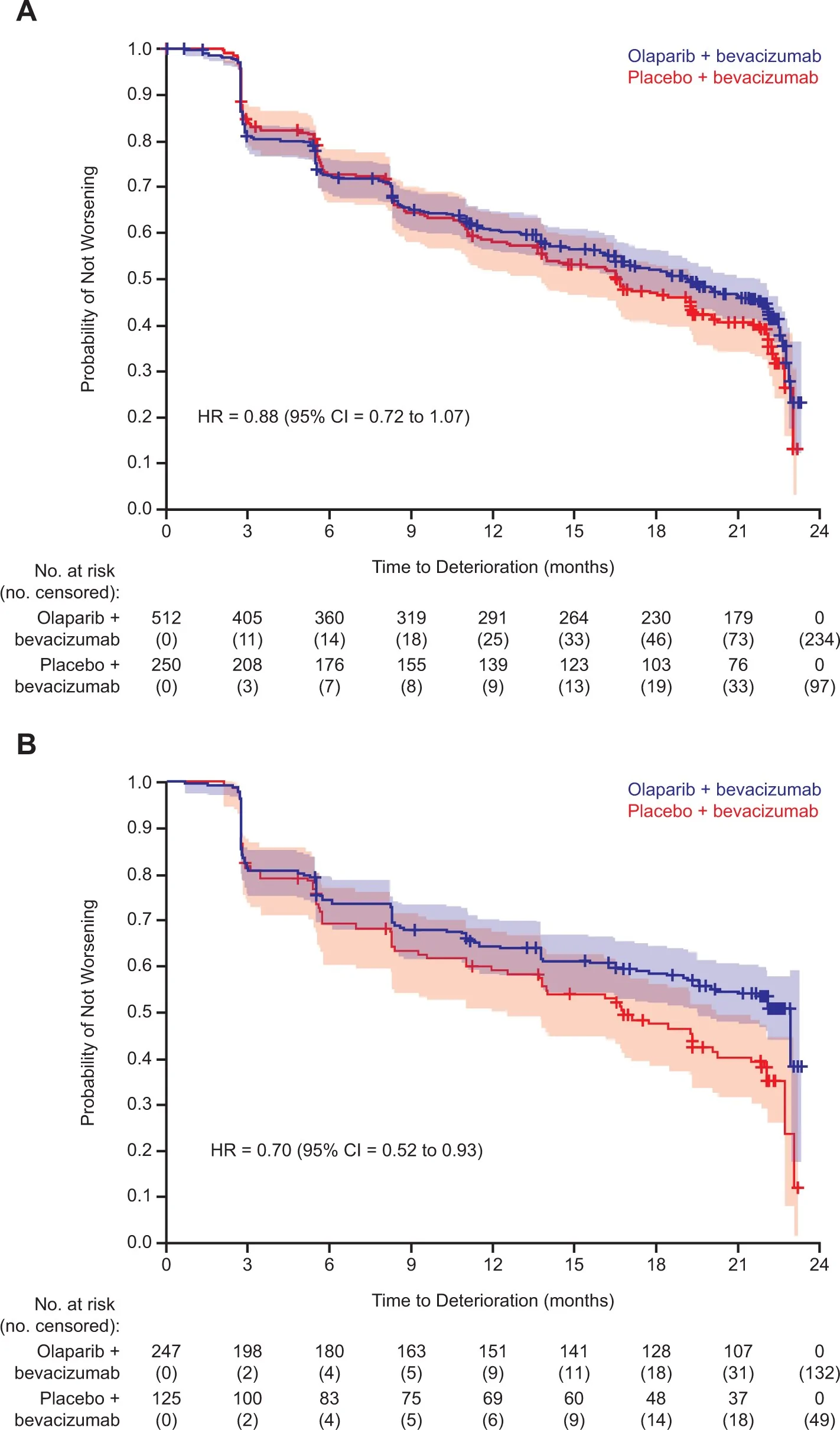
Time until definitive health-related quality of life deterioration of European Organisation for Research and Treatment of Cancer Core Quality of Life Questionnaire Global Health Status scores **(A)** in the modified intention-to-treat population and **(B)** in the homologous recombination deficiency–positive subgroup. In the modified intention-to-treat population, there were 375 events of health-related quality of life deterioration and 56 deaths. In the homologous recombination deficiency–positive subgroup, there were 181 events of health-related quality of life deterioration and 10 deaths.

**Figure 5.**
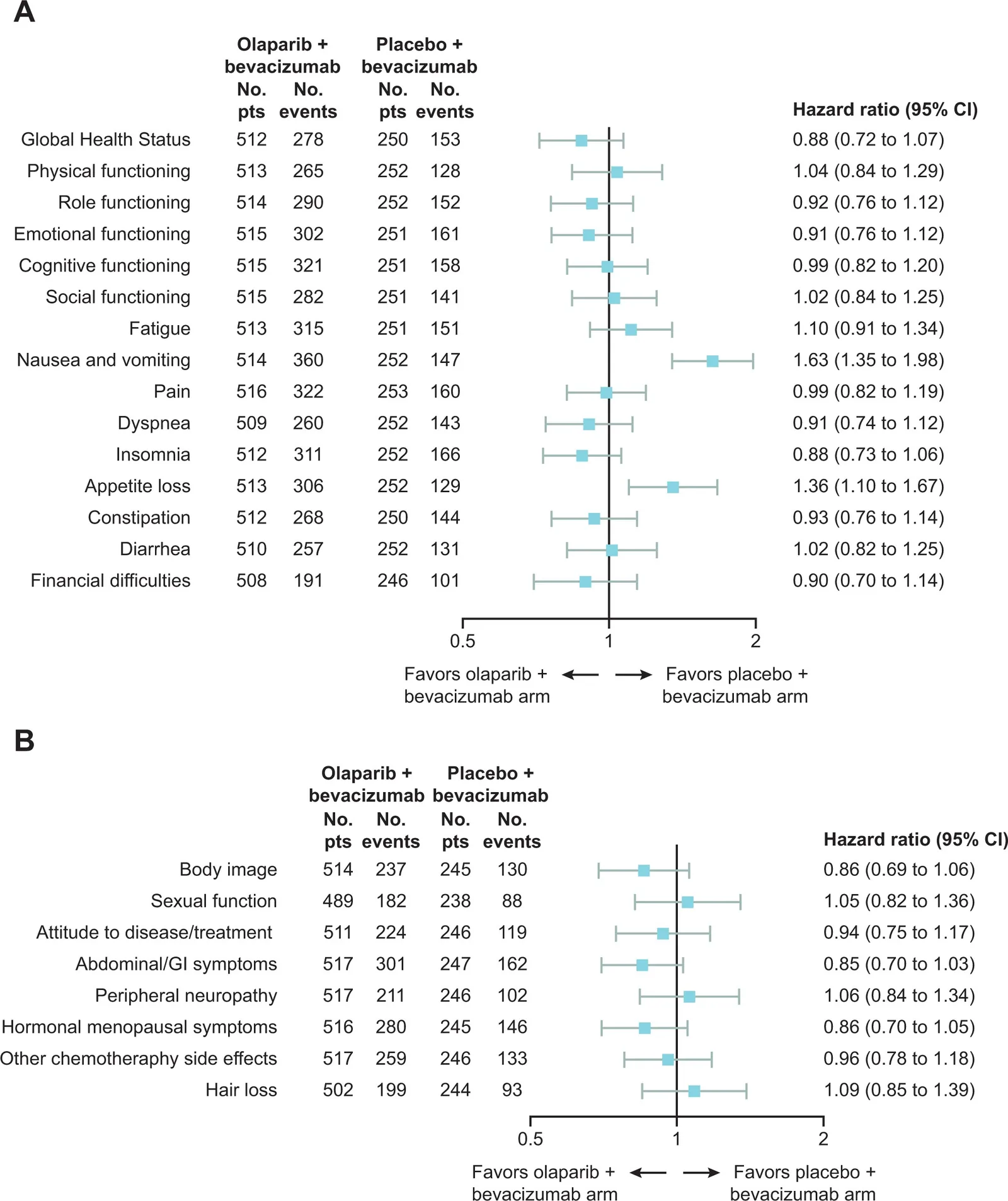
Time until definitive deterioration of the **(A)** European Organisation for Research and Treatment of Cancer Core Quality of Life Questionnaire scales and **(B)** European Organisation for Research and Treatment of Cancer Quality of Life Questionnaire Ovarian Cancer module scales in the modified intention-to-treat population. Error bars represent 95% confidence intervals. Abbreviations: GI = gastrointestinal; pts = patients.

### Time without clinically significant symptoms or toxicity in the overall population and by homologous recombination deficiency status

Median time without clinically significant symptoms or toxicity statistically significantly (*P* < .0001) favored olaparib plus bevacizumab over placebo plus bevacizumab overall and in the homologous recombination deficiency–positive subgroup for all 3 toxicity states (see Supplementary Results and Figure S7).

## Discussion

The addition of olaparib to bevacizumab had no detrimental effect on global HRQoL, indicating that HRQoL was maintained in women with newly diagnosed advanced ovarian cancer, either overall or in the homologous recombination deficiency–positive subgroup.

The most common nonhematologic adverse events reported by patients receiving olaparib include fatigue, nausea, and vomiting.^3^^,^^11^^,^^12^ Although worsening in QLQ-C30 scores for fatigue, nausea or vomiting, and appetite loss appeared to peak at week 12 with olaparib plus bevacizumab, it should be noted that symptom scores at earlier timepoints were not available as patients did not complete their first postbaseline questionnaire until week 12. Particular attention should be paid to adverse events during the first few months of maintenance therapy during routine clinical practice to optimize adherence. EORTC QLQ-C30 scores for fatigue, nausea or vomiting, and appetite loss resolved to the level of placebo plus bevacizumab over time, which could be explained by habituation or dose modification of olaparib. Time until definitive HRQoL deterioration hazard ratios for QLQ-C30 appetite loss and nausea and vomiting also favored placebo plus bevacizumab over olaparib plus bevacizumab, suggesting that these symptoms may be bothersome in some patients receiving olaparib plus bevacizumab. Importantly, in the homologous recombination deficiency–positive subgroup, no clinically relevant differences between treatment arms were seen in mean change from baseline in HRQoL scores, suggesting that maintenance olaparib was not detrimental to HRQoL in this subgroup. Furthermore, olaparib plus bevacizumab therapy resulted in time until definitive HRQoL deterioration benefit in the homologous recombination deficiency–positive subgroup. Interestingly, constipation was not found to be a major HRQoL symptom for patients receiving olaparib plus bevacizumab in PAOLA-1, whereas it was worse over time in patients who received maintenance niraparib in the PRIMA trial.^13^

Despite the inclusion of bevacizumab in PAOLA-1, our findings are consistent with those seen with maintenance olaparib monotherapy in the phase III SOLO1 trial in patients with newly diagnosed advanced ovarian cancer and a BRCA mutation.^1^ In SOLO1, maintenance olaparib monotherapy had no detrimental effect on HRQoL.^1^

Guidelines for analyzing HRQoL in cancer trials recommend evaluating time-to-event outcomes and the magnitude of events (improvement or worsening using the linear mixed model),^14^ rather than evaluating endpoints such as time without clinically significant symptoms or toxicity. However, time without clinically significant symptoms or toxicity provides important insight into the effect of maintenance treatment on otherwise symptom-free patients, and olaparib monotherapy demonstrated a substantial time without clinically significant symptoms or toxicity benefit in SOLO1.^1^ In our post hoc exploratory analysis of the ITT population, we observed a substantial, statistically significant time without clinically significant symptoms or toxicity benefit across 3 toxicity states, demonstrating robustness of the time without clinically significant symptoms or toxicity results. Furthermore, a recent analysis of the association between toxicity and QoL supported selection of all grade 2 or higher adverse events with symptoms to accurately reflect QoL.^15^ An even greater time without clinically significant symptoms or toxicity benefit was observed with olaparib plus bevacizumab over placebo plus bevacizumab in the homologous recombination deficiency–positive subgroup, which may reflect the substantial PFS benefit observed in the homologous recombination deficiency–positive subgroup.^3^ These findings support the association between time without clinically significant symptoms or toxicity and HRQoL and suggest that the delayed disease progression^3^ and clinically meaningful overall survival benefit^6^ seen with olaparib plus bevacizumab vs placebo plus bevacizumab in patients with homologous recombination deficiency–positive tumors was not offset by adverse effects of maintenance olaparib.

In our post hoc exploratory combined treatment arm analysis of HRQoL before and after RECIST progression, most QLQ-C30 domains (including GHS) revealed postprogression deterioration in HRQoL, with clinically meaningful deterioration in emotional functioning and social functioning. Deterioration of HRQoL following first recurrence in patients with advanced ovarian cancer has been demonstrated in several studies.^1^^,^^2^^,^^16^ Anxiety and depression items in the EuroQol 5-Dimension 5-Level, which partially correspond to emotional functioning in the QLQ-C30, were the most frequently affected domains postprogression in SOLO1.^1^ Of QLQ-C30 domains assessed in the phase III PRIMA trial of maintenance niraparib, only emotional functioning exhibited clinically meaningful deterioration postprogression, which was sustained throughout the 24-week follow-up period.^2^ These studies indicate an emotional burden associated with disease progression. Poly(ADP-ribose) polymerase inhibitor maintenance therapy has the potential to help a proportion of patients avoid the substantial burden associated with disease progression; in the homologous recombination deficiency–positive subgroup in PAOLA-1, 46.1% of patients in the olaparib plus bevacizumab arm had not relapsed at 5 years vs 19.2% in the placebo plus bevacizumab arm.^6^

The limitations of PAOLA-1 have been described previously^3^ and include the absence of an olaparib monotherapy comparator arm and lack of prespecified analyses by homologous recombination deficiency status from the multiple testing procedure. Low compliance can be a challenge for patient-reported outcomes studies;^17^^,^^18^ however, compliance rates in this analysis were high throughout the study with no notable difference between treatment arms. Disease progression adversely affected HRQoL data collection, with postprogression questionnaires completed by only 22.2% of patients. Health-related QoL instruments administered via electronic devices are now available, which may facilitate higher completion rates and mitigate the risk of collection bias in future studies. Other digital tools (eg, smartphone apps) may also prove useful for monitoring HRQoL.^19^ Underestimation or overestimation of patient-reported HRQoL may result from underreporting symptoms during remission and overreporting symptoms following relapse. Fatigue and asthenia was one of the most common adverse events reported in PAOLA-1;^3^ the 3 QLQ-C30 items contributing to the fatigue score can be easily implemented in clinical practice. A specific questionnaire to analyze fatigue (EORTC QLQ-FA12) is also available. Wearable technologies also have potential for monitoring symptoms such as fatigue.^20^ Furthermore, although there were instances of missing data, this was likely mitigated by the large sample size and high compliance rates.

In conclusion, the addition of maintenance olaparib to bevacizumab had no clinically meaningful impact on global HRQoL either overall or in the homologous recombination deficiency–positive subgroup. Time until definitive HRQoL deterioration estimates of QLQ-C30 GHS favored olaparib plus bevacizumab over placebo plus bevacizumab. Maintenance therapy with olaparib plus bevacizumab may allow an important fraction of patients with homologous recombination deficiency–positive ovarian cancer to evade the substantial burden imposed by disease progression. In conclusion, in patients with newly diagnosed advanced ovarian cancer in response after first-line platinum-based chemotherapy plus bevacizumab, the addition of olaparib provided a substantial PFS benefit while maintaining HRQoL.

## Supplementary Material

djag122_Supplementary_Data

## Data Availability

ARCAGY-GINECO has a long history of academic data sharing for research purposes. The process is similar for every trial sponsored by ARCAGY-GINECO (or ARCAGY Research). Researchers have to submit a request to the sponsor directly or through the principal investigator. The request should be written in a predefined format of a short synopsis indicating the objective of the research, the methodology intended to be used, including the statistical analysis plan, and the variables within the database required for the research. A scientific board will review and approve the requests on a case-by-case basis. Only encoded datasets will be used, which enables us to fulfill legal and ethical obligations to protect our patients while at the same time utilizing patient data in progressing medical research to its full potential in the best interests of public health. A specific agreement between the sponsor and the researcher is requested for data transfer. This data transfer agreement details both parties’ responsibilities to ensure the required level of data integrity and legal and ethical obligations. In the case of sharing encoded patient-level data, please note that the full dataset may not be shared in view of the following: clinical consent for some countries prohibits secondary use of the data; patients may withdraw their consent for participation in the trial at any point; other aspects might also be taken into consideration to protect patient privacy (eg, review of rare clinical events where information is aggregated to a higher level before sharing).
