# Supplementary material for: Health-related quality of life in patients with newly diagnosed advanced ovarian cancer receiving maintenance olaparib plus bevacizumab (PAOLA-1/ENGOT-ov25)
Source: J Natl Cancer Inst. 2026 Apr 24;118(10):1874–87. doi: 10.1093/jnci/djag122 (PMC13650380; doi:10.1093/jnci/djag122)
Supplement: Supplementary file 1 [file djag122_supplementary_data.pdf]

Data Supplement

Health-related quality of life in patients with newly diagnosed advanced ovarian cancer receiving maintenance olaparib plus bevacizumab (PAOLA-1/ENGOT-ov25)

Joly F, Anota A, Chabaud S, et al

This Data Supplement has been provided by the authors to give readers additional information about this work.

Contents

Methods 3

Full eligibility criteria 3

Inclusion criteria 3

Exclusion criteria 4

Mean change from baseline in health-related quality of life (HRQoL) scores: descriptive analyses over time 7

Mean change from baseline in HRQoL scores: mixed model for repeated measures (MMRM) 7

Time until definitive health-related quality of life deterioration (TUDD) 7

Time without significant symptoms or toxicity (TWiST) 7

Figure S1. TWiST analysis 8

Results 9

TWiST in the overall population and by homologous recombination deficiency (HRD) status 9

Table S1. EORTC QLQ-C30 compliance and completion rates at baseline and each follow-up visit in the ITT population 10

Table S2. EORTC QLQ-OV28 compliance and completion rates at baseline and each follow-up visit in the ITT population 11

Table S3. Reasons for EORTC QLQ-C30 or EORTC QLQ-OV28 questionnaires not being completed 12

Table S4. Baseline EORTC QLQ-C30 scores and adjusted mean change from baseline in the modified ITT population 14

Table S5. Baseline EORTC QLQ-OV28 scores and adjusted mean change from baseline in the modified ITT population 22

Table S6. Baseline EORTC QLQ-C30 scores and adjusted mean change from baseline in the HRD-positive subgroup 27

Table S7. Baseline EORTC QLQ-OV28 scores and adjusted mean change from baseline in the HRD-positive subgroup 35

Table S8. Baseline EORTC QLQ-C30 scores and mean change from baseline in the HRD-negative subgroup 40

Table S9. Baseline EORTC QLQ-OV28 scores and adjusted mean change from baseline in the HRD-negative subgroup 48

Table S10. Mean change in EORTC QLQ-C30 scores post-progression by treatment arm 53

Figure S2. Adjusted mean change over time in EORTC QLQ-C30 scores for (A) physical functioning, (B) role functioning, (C) emotional functioning, (D) cognitive functioning, and (E) social functioning in the modified ITT population 54

Figure S3. Adjusted mean change over time in EORTC QLQ-C30 scores for (A) pain, (B) dyspnea, and (C) diarrhea in the modified ITT population 57

Figure S4. Adjusted mean change over time in EORTC QLQ-OV28 scores for (A) body image, (B) Sexual function, (C) attitude to disease/treatment, (D) abdominal/GI symptoms, (E) peripheral neuropathy, (F) hormonal/menopause symptoms, (G) other chemotherapy side effects, and (H) hair loss in the modified ITT population 59

Figure S5. Between-group difference in adjusted mean global change from baseline for (A) functional scales and (B) symptomatic scales of the EORTC QLQ-C30 by HRD status[a] 64

Figure S6. Between-group difference in adjusted mean global change from baseline for (A) functional scales and (B) symptomatic scales of the EORTC QLQ-OV28 by HRD status[a] 66

Figure S7. TWiST by HRD status (HRD-positive and HRD-negative/unknown subgroups) for TOX defined as (A) all grade ≥2 AEs with symptoms, (B) grade ≥2 AEs with symptoms associated with olaparib (fatigue/asthenia, nausea, or vomiting), and (C) all grade ≥2 AEs. 67

Methods

Full eligibility criteria

Inclusion criteria

For inclusion in the study, patients should fulfill the following criteria:

1. Female and aged ≥18 years old
2. Signed informed consent and ability to comply with treatment and follow-up
3. Patients with newly diagnosed:
    - Ovarian cancer, primary peritoneal cancer, and/or fallopian tube cancer
    - Histologically confirmed (based on local histopathological findings):
        - High-grade serous or
        - High-grade endometrioid or
        - Other epithelial non-mucinous ovarian cancer in patients with a deleterious germline *BRCA1* and/or *BRCA2* mutation
    - Advanced stage: International Federation of Gynecology and Obstetrics (FIGO) stage IIIB, IIIC, or IV (1988 FIGO classification):
        - Using the current 2014 FIGO classification for stage III disease, women would be classified as having stage IIIA-IV ovarian cancer for this to be considered advanced stage
4. Completed first-line platinum-taxane chemotherapy prior to randomization:
    - Platinum-taxane-based regimen must have consisted of a minimum of six and a maximum of nine treatment cycles. However, if platinum-based therapy was discontinued early as a result of nonhematological toxicity specifically related to the platinum regimen (including neurotoxicity and hypersensitivity), the patient must have received a minimum of four cycles of the platinum regimen
    - Intravenous, intraperitoneal, or neoadjuvant platinum-based chemotherapy is allowed. For weekly therapy, 3 weeks is considered one cycle. Interval debulking surgery is allowed
5. Prior to randomization, patients must have received a minimum of three cycles of bevacizumab in combination with the three last cycles of platinum-based chemotherapy. Only in the case of interval cytoreductive surgery are patients permitted to receive only two cycles of bevacizumab in combination with the last three cycles of platinum-based chemotherapy. Bevacizumab treatment should be administered at a dose 15 mg/kg every 3 weeks up to a total of 15 months
6. Prior to randomization, patients must be without evidence of disease (NED) or in complete response or partial response from the first-line treatment. There should be no clinical evidence of disease progression (through physical examination, imaging, or cancer antigen [CA]-125 test) throughout first-line treatment and prior to study randomization:
    - Patients without assessable disease after initial cytoreductive surgery were considered to have NED at the end of the first-line chemotherapy and surgery strategy if disease has not progressed
    - Patients with measurable or assessable disease after initial cytoreductive surgery or at the start of neoadjuvant chemotherapy and whose disease was no

longer detectable at the end of the chemotherapy and surgery strategy were considered to have a complete response

7. Patients must be randomized at least 3 weeks and no more than 9 weeks after their last dose of chemotherapy (last dose is the day of the last infusion) and all major toxicities from prior chemotherapy must have resolved to Common Terminology Criteria for Adverse Events (CTCAE) grade 1 or better (except alopecia and peripheral neuropathy)
8. Patients must have normal organ and bone marrow function:
   - Hemoglobin ≥10.0 g/dL
   - Absolute neutrophil count ≥1.5 × 10^9/L
   - Platelet count ≥100 × 10^9/L
   - Total bilirubin ≤1.5 × institutional upper limit of normal (ULN)
   - Aspartate aminotransferase/serum glutamic oxaloacetic transaminase, and alanine aminotransferase/serum glutamic pyruvate transaminase ≤2.5 × ULN unless liver metastases are present, in which case they must be ≤5 × ULN
   - Serum creatinine ≤1.5 × institutional ULN
   - Patients not receiving anticoagulant medication who have an international normalized ratio (INR) ≤1.5 and an activated prothrombin time (aPTT) ≤1.5 × ULN. The use of full-dose oral or parenteral anticoagulants is permitted as long as the INR or aPTT is within therapeutic limits (according to the site's medical standard). If the patient is on oral anticoagulants, the dose must be stable for at least 2 weeks at the time of randomization
   - Urine dipstick for proteinuria <2+. If urine dipstick is ≥2+, 24-hour urine must demonstrate <1 g of protein in 24 hours
   - Normal blood pressure (BP) or adequately treated and controlled hypertension (systolic BP ≤140 mmHg and/or diastolic BP ≤90 mmHg)
9. Eastern Cooperative Oncology Group performance status 0-1
10. Formalin-fixed, paraffin-embedded tumor sample from the primary cancer must be available for central BRCA testing and test result must be available for stratification
11. Postmenopausal or evidence of non-childbearing status for women of childbearing potential prior to the first dose of study treatment
12. For France only: in France, a patient will be eligible for randomization in this study only if either affiliated to, or a beneficiary of, a social security category

Exclusion criteria

Patients must not enter the study if any of the following exclusion criteria are fulfilled:

1. Non-epithelial origin of the ovary, the fallopian tube or the peritoneum (ie, germ cell tumors)
2. Ovarian tumors of low malignant potential (eg, borderline tumors) or mucinous carcinoma
3. Synchronous primary endometrial cancer, unless both of the following criteria are met:
   - Stage <II

- Aged <60 years at the time of diagnosis of endometrial cancer with stage IA or IB grade I or II, or stage IA grade III endometrial carcinoma OR aged ≥60 years at the time of diagnosis of endometrial cancer with stage IA grade I or II endometrioid adenocarcinoma. Patients with serous or clear-cell adenocarcinoma or carcinosarcoma of the endometrium are not eligible

4. Other malignancy within the last 5 years except: (1) adequately treated nonmelanoma skin cancer; (2) curatively treated *in situ* cancer of the cervix; or (3) ductal carcinoma *in situ.* Patients with a history of localized malignancy diagnosed over 5 years ago may be eligible provided they completed adjuvant systemic therapy prior to randomization and remain free of recurrent or metastatic disease. Patients with a history of primary triple-negative breast cancer may be eligible provided they completed definitive anticancer treatment >3 years ago and remain breast cancer free prior to the start of study treatment
5. Patients with myelodysplastic syndromes (MDS)/acute myeloid leukemia (AML) history
6. Patients who, for at least one cycle, experienced a delay of >2 weeks due to prolonged hematologic recovery during first-line chemotherapy
7. Patients receiving radiotherapy within 6 weeks prior to study treatment
8. Major surgery within 4 weeks of starting study treatment. Patients must have recovered from any effects of any major surgery
9. Previous allogeneic bone marrow transplantation
10. Any previous treatment with poly(ADP-ribose) polymerase inhibitor, including olaparib
11. Administration of other simultaneous chemotherapy drugs, any other anticancer therapy or antineoplastic hormonal therapy, or simultaneous radiotherapy during the trial treatment period (hormonal replacement therapy is permitted as are steroidal antiemetics)
12. Current or recent (within 10 days prior to randomization) chronic use of aspirin >325 mg/day
13. Concomitant use of known potent cytochrome P450 3A4 inhibitors, such as ketoconazole, itraconazole, ritonavir, indinavir, saquinavir, telithromycin, clarithromycin, and nelfinavir
14. Prior history of hypertensive crisis (CTCAE grade 4) or hypertensive encephalopathy
15. Clinically significant (eg, active) cardiovascular disease, including:
    - Myocardial infarction or unstable angina pectoris within ≤6 months of randomization
    - New York Heart Association grade ≥2 congestive heart failure
    - Poorly controlled cardiac arrhythmia despite medication (patients with rate-controlled atrial fibrillation are eligible), or any clinically significant abnormal finding on a resting electrocardiogram
    - Peripheral vascular disease grade ≥3 (eg, symptomatic and interfering with activities of daily living requiring repair or revision)
16. Previous cerebrovascular accident, transient ischemic attack or subarachnoid hemorrhage within 6 months prior to randomization
17. History or evidence of hemorrhagic disorders within 6 months prior to randomization

18. Evidence of bleeding diathesis or significant coagulopathy (in the absence of coagulation)
19. History or clinical suspicion of brain metastases or spinal cord compression. Computed tomography (CT)/magnetic resonance imaging (MRI) of the brain is mandatory (within 4 weeks prior to randomization) in the case of suspected brain metastases. Spinal MRI is mandatory (within 4 weeks prior to randomization) in the case of suspected spinal cord compression
20. History or evidence upon neurological examination of central nervous system disease (eg, uncontrolled seizures), unless adequately treated with standard medical therapy
21. Significant traumatic injury during 4 weeks prior to randomization
22. Non-healing wound, active ulcer, or bone fracture. Patients with granulating incisions healing by secondary intention with no evidence of facial dehiscence or infection are eligible, but require 3-weekly wound examinations
23. History of vascular endothelial growth factor therapy-related abdominal fistula, gastrointestinal perforation, or active gastrointestinal bleeding within 6 months prior to the first study treatment
24. Current clinically relevant bowel obstruction, including sub-occlusive disease, related to the underlying disease
25. Patients with evidence of abdominal free air not explained by paracentesis or a recent surgical procedure
26. Evidence of any other disease, metabolic dysfunction, physical examination finding, or laboratory finding giving reasonable suspicion of a disease or condition that contraindicates the use of an investigational drug or puts the patient at high risk for treatment-related complications
27. Pregnant or lactating women
28. Participation in another clinical study with an investigational product during the chemotherapy cycle immediately prior to randomization
29. Patients unable to swallow orally administered medication and patients with gastrointestinal disorders likely to interfere with absorption of the study medication
30. Patients with a known hypersensitivity to olaparib or any of the excipients of the product
31. Immunocompromised patients, for example, with known active hepatitis (ie, hepatitis B or C) because of the risk of transmitting the infection through blood or other body fluids or patients known to be serologically positive for HIV

Mean change from baseline in health-related quality of life (HRQoL) scores: descriptive analyses over time

The actual mean change from baseline for each health-related quality of life (HRQoL) score was derived for each visit where there was available data and depicted graphically. At each follow-up timepoint, the mean change from baseline was represented for each treatment arm with its associated 95% confidence interval (CI).

Mean change from baseline in HRQoL scores: mixed model for repeated measures (MMRM)

The adjusted mean change in each HRQoL scale was analyzed by mixed model for repeated measures (MMRM) until 2 years of follow-up. To compare the treatment arms over time, adjusted mean estimates per treatment arm and associated 95% CIs were calculated with estimates of the between-treatment difference and the associated 95% CI.

Longitudinal analysis using MMRM was conducted separately for each of the HRQoL scales. The analysis included all questionnaires received up to 2 years of follow-up. It included all scores from baseline up to the scheduled visit at 2 years if at least 20 patients in each treatment arm had a score. The model included a treatment arm effect, a time effect, and a time by treatment interaction effect, and was adjusted on the baseline score and the baseline by time interaction effect. The model also included random effects on patients to reflect individual deviance from the intercept. An unstructured covariance matrix was considered.

Time until definitive health-related quality of life deterioration (TUDD)

Time until definitive health-related quality of life deterioration (TUDD) was defined as the time interval between randomization and the occurrence of the first clinically relevant deterioration in the HRQoL score from baseline with no further clinically relevant improvement from baseline, or all-cause death. The clinical relevance of the deterioration was qualified by the minimal important difference (MID), which was fixed to 10.0 points for each score. Patients who were alive with no deterioration before they dropped out of the study and those with deterioration followed by significant improvement were censored at the time of their last HRQoL assessment.

The Kaplan-Meier method was used to generate time-to-event curves for TUDD with a univariate Cox model used to estimate HRs and 95% CIs.

Time without significant symptoms or toxicity (TWiST)

Time without significant symptoms or toxicity (TWiST) was calculated as the total number of days without symptoms or toxicity (TOX) that occurred between randomization and first disease progression or censoring for progression (progression-free survival [PFS]

minus TOX) (see **Figure S1**) in the intention-to-treat population and by homologous recombination deficiency (HRD) status. In the TWiST analyses, three definitions of TOX state were considered: all grade ≥2 adverse events (AEs) with symptoms; grade ≥2 AEs with symptoms associated with olaparib (fatigue/asthenia, nausea, or vomiting); and all grade ≥2 AEs. Overlapping TOX intervals were not doubly counted. TWiST was presented using Kaplan-Meier curves of TOX and PFS. All p-values reported are nominal.

Figure S1. TWiST analysis

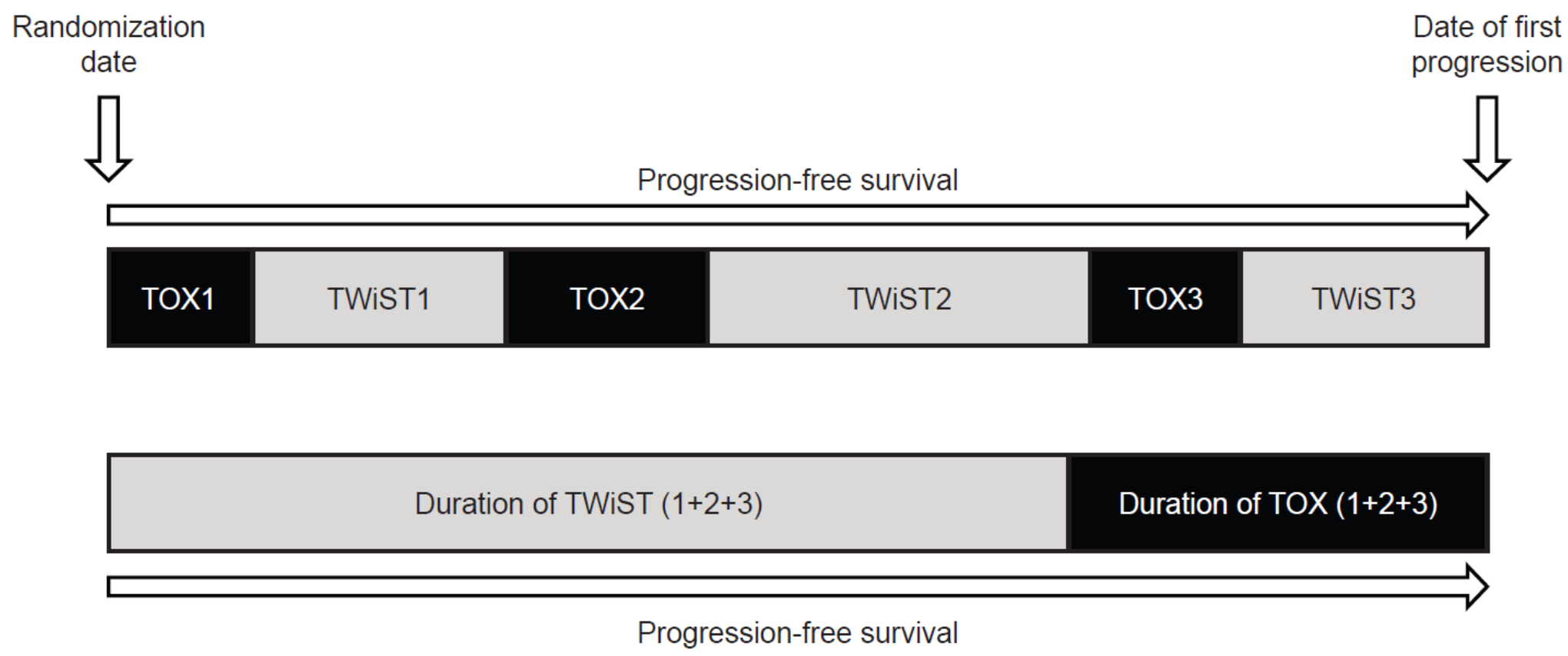

TOX = toxicity; TWiST = time without symptoms or toxicity.

Results

TWiST in the overall population and by homologous recombination deficiency (HRD) status

At the March 22, 2019 data cutoff, 677 patients (467 and 210 in the olaparib plus bevacizumab and placebo plus bevacizumab arms, respectively) reported at least one grade ≥2 AE *with* symptoms, 304 patients (245 and 59, respectively) reported at least one grade ≥2 AE associated with olaparib (fatigue/asthenia, nausea, or vomiting), and 769 (522 and 247, respectively) patients reported at least one grade ≥2 AE.

Median (95% CI) TWiST for all grade ≥2 AEs with symptoms with olaparib plus bevacizumab versus placebo plus bevacizumab was 17.2 (16.0 to 20.1) versus 11.3 (9.7 to 13.6) months (nominal $P<.0001$) in the overall population and 36.6 (22.4 to not estimable [NE]) versus 12.6 (9.8 to 15.6) months (nominal $P<.0001$) in the HRD-positive subgroup (**Figure S7A**).

For grade ≥2 AEs with symptoms associated with olaparib, median (95% CI) TWiST with olaparib plus bevacizumab versus placebo plus bevacizumab was 21.9 (20.2 to 23.5) versus 16.6 (14.6 to 18.0) months (nominal $P<.0001$) in the overall population and 36.6 (31.9 to NE) versus 17.4 (15.1 to 19.4) months (nominal $P<.0001$) in the HRD-positive subgroup (**Figure S7B**).

For all grade ≥2 AEs, median (95% CI) TWiST with olaparib plus bevacizumab versus placebo plus bevacizumab was 14.1 (12.5 to 16.1) versus 7.7 (5.9 to 9.1) months (nominal $P<.0001$) in the overall population and 24.4 (19.3 to NE) versus 7.4 (5.8 to 11.2) months (nominal $P<.0001$) in the HRD-positive subgroup (**Figure S7C**).

The difference in median TWiST between treatment arms did not reach nominal significance ($P<.05$) in the HRD-negative/unknown subgroup, irrespective of the type of AEs included (**Figure S7**).

Table S1. EORTC QLQ-C30 compliance and completion rates at baseline and each follow-up visit in the ITT population

Time	Olaparib plus bevacizumab (N = 537)			Placebo plus bevacizumab (N = 269)		
	Forms received/ expected	Compliance (%)	Completion (%)	Forms received/ expected	Compliance (%)	Completion (%)
Baseline	516/537	96.1	96.1	253/269	94.1	94.1
W12	483/528	91.5	89.9	243/266	91.4	90.3
W24	459/522	87.9	85.5	224/262	85.5	83.3
W36	420/509	82.5	78.2	213/257	82.9	79.2
W48	414/495	83.6	77.1	199/248	80.2	74.1
W60	377/475	79.4	70.2	183/239	76.6	68.0
W72	362/459	78.9	67.4	178/227	78.4	66.2
W84	330/438	75.3	61.5	166/218	76.1	61.7
W96	302/419	72.1	56.2	147/207	71.0	54.6

The compliance rate was defined as the number of patients who completed at least one score at the specific timepoint divided by the number of patients expected to complete the assessment at that visit (that is, patients who were alive and still in the study at that timepoint). The completion rate at a specific timepoint was defined as the number of patients who completed at least one score at that specific timepoint divided by the number of patients in the ITT population.

EORTC QLQ-C30 = European Organisation for Research and Treatment of Cancer Core Quality of Life Questionnaire; ITT = intention-to-treat; W = Week.

Table S2. EORTC QLQ-OV28 compliance and completion rates at baseline and each follow-up visit in the ITT population

Time	Olaparib plus bevacizumab (N = 537)			Placebo plus bevacizumab (N = 269)		
	Forms received/ expected	Compliance (%)	Completion (%)	Forms received/ expected	Compliance (%)	Completion (%)
Baseline	517/537	96.3	96.3	249/269	92.6	92.6
W12	484/528	91.7	90.1	241/266	90.6	89.6
W24	456/522	87.4	84.9	224/262	85.5	83.3
W36	418/509	82.1	77.8	213/257	82.9	79.2
W48	414/495	83.6	77.1	198/248	79.8	73.6
W60	377/475	79.4	70.2	183/239	76.6	68.0
W72	361/459	78.6	67.2	178/227	78.4	66.2
W84	330/438	75.3	61.5	166/218	76.1	61.7
W96	301/419	71.8	56.1	147/207	71.0	54.6

The compliance rate was defined as the number of patients who completed at least one score at the specific timepoint divided by the number of patients expected to complete the assessment at that visit (that is, patients who were alive and still in the study at that timepoint). The completion rate at a specific timepoint was defined as the number of patients who completed at least one score at that specific timepoint divided by the number of patients in the ITT population.

EORTC QLQ-OV28 = European Organisation for Research and Treatment of Cancer Quality of Life Questionnaire Ovarian Cancer module; ITT = intention-to-treat; W = Week.

Table S3. Reasons for EORTC QLQ-C30 or EORTC QLQ-OV28 questionnaires not being completed

	Olaparib plus bevacizumab (N = 537)	Placebo plus bevacizumab (N = 269)
**Baseline**		
Completed,[a] n (%)	517 (96.3)	253 (94.1)
Missing despite being expected, n (%)	20 (3.7)	16 (5.9)
Site missed administration[b]	6 (30.0)	7 (43.8)
Patient did not comply (not due to illness)[b]	4 (20.0)	2 (12.5)
Patient too ill[b]	0	0
Other[b]	2 (10.0)	1 (6.3)
Missing	8 (40.0)	6 (37.5)
**W12**		
Completed,[a] n (%)	484 (90.1)	243 (90.3)
Missing despite being expected, n (%)	44 (8.2)	23 (8.6)
Site missed administration[b]	32 (72.7)	14 (60.9)
Patient did not comply (not due to illness)[b]	4 (9.1)	3 (13.0)
Patient too ill[b]	2 (4.5)	1 (4.3)
Other[b]	5 (11.4)	2 (8.7)
Missing	1 (2.3)	3 (13.0)
**W24**		
Completed,[a] n (%)	459 (85.5)	224 (83.3)
Missing despite being expected, n (%)	63 (11.7)	38 (14.1)
Site missed administration[b]	42 (66.7)	24 (63.2)
Patient did not comply (not due to illness)[b]	5 (7.9)	4 (10.5)
Patient too ill[b]	2 (3.2)	1 (2.6)
Other[b]	7 (11.1)	3 (7.9)
Missing	7 (11.1)	6 (15.8)
**W36**		
Completed,[a] n (%)	420 (78.2)	213 (79.2)
Missing despite being expected, n (%)	89 (16.6)	44 (16.4)
Site missed administration[b]	56 (62.9)	27 (61.4)
Patient did not comply (not due to illness)[b]	10 (11.2)	6 (13.6)
Patient too ill[b]	3 (3.4)	0
Other[b]	5 (5.6)	2 (4.5)
Missing	15 (16.9)	9 (20.5)
**W48**		
Completed,[a] n (%)	414 (77.1)	199 (74.0)
Missing despite being expected, n (%)	81 (15.1)	49 (18.2)
Site missed administration[b]	45 (55.6)	24 (49.0)
Patient did not comply (not due to illness)[b]	7 (8.6)	9 (18.4)
Patient too ill[b]	5 (6.2)	2 (4.1)
Other[b]	5 (6.2)	4 (8.2)
Missing	19 (23.5)	10 (20.4)

	Olaparib plus bevacizumab (N = 537)	Placebo plus bevacizumab (N = 269)
**W60**		
Completed,[a] n (%)	377 (70.2)	183 (68.0)
Missing despite being expected, n (%)	98 (18.2)	56 (20.8)
Site missed administration[b]	57 (58.2)	22 (39.3)
Patient did not comply (not due to illness)[b]	7 (7.1)	11 (19.6)
Patient too ill[b]	1 (1.0)	2 (3.6)
Other[b]	5 (5.1)	3 (5.4)
Missing	28 (28.6)	18 (32.1)
**W72**		
Completed,[a] n (%)	362 (67.4)	178 (66.2)
Missing despite being expected, n (%)	97 (18.1)	49 (18.2)
Site missed administration[b]	49 (50.5)	19 (38.8)
Patient did not comply (not due to illness)[b]	8 (8.2)	11 (22.4)
Patient too ill[b]	6 (6.2)	1 (2.0)
Other[b]	8 (8.2)	4 (8.2)
Missing	26 (26.8)	14 (28.6)
**W84**		
Completed,[a] n (%)	330 (61.5)	166 (61.7)
Missing despite being expected, n (%)	108 (20.1)	52 (19.3)
Site missed administration[b]	66 (61.1)	26 (50.0)
Patient did not comply (not due to illness)[b]	10 (9.3)	4 (7.7)
Patient too ill[b]	3 (2.8)	1 (1.9)
Other[b]	5 (4.6)	7 (13.5)
Missing	24 (22.2)	14 (26.9)
**W96**		
Completed,[a] n (%)	302 (56.2)	147 (54.6)
Missing despite being expected, n (%)	117 (21.8)	60 (22.3)
Site missed administration[b]	44 (37.6)	34 (56.7)
Patient did not comply (not due to illness)[b]	10 (8.5)	5 (8.3)
Patient too ill[b]	5 (4.3)	1 (1.7)
Other[b]	7 (6.0)	3 (5.0)
Missing	51 (43.6)	17 (28.3)

Percentages may not total 100 because of rounding.

[a]Patients had completed at least one score in either the EORTC QLQ-C30 or EORTC QLQ-OV28 questionnaire.

[b]One of the four reasons on the electronic case report form for the investigator to select from if a patient did not complete the EORTC QLQ-C30 or EORTC QLQ-OV28 questionnaires at scheduled visits.

EORTC QLQ-C30 = European Organisation for Research and Treatment of Cancer Core Quality of Life Questionnaire; EORTC QLQ-OV28 = European Organisation for Research and Treatment of Cancer Quality of Life Questionnaire Ovarian Cancer module; W = Week.

Table S4. Baseline EORTC QLQ-C30 scores and adjusted mean change from baseline in the modified ITT population

Scale scores	Olaparib plus bevacizumab (N = 517)	Placebo plus bevacizumab (N = 253)	Between-group difference	Treatment-by-time interaction
Functional scales				
Global Health Status				
Baseline	N = 512	N = 250		
Mean (SD)	68.6 (18.0)	67.2 (16.2)		
Median (range)	66.7 (0-100)	66.7 (25-100)		
Adjusted mean global change from baseline (95% CI)	−1.12 (−2.21 to −0.02)	−2.76 (−4.34 to −1.19)	1.65 (−0.27 to 3.56)	*P*=.0015
Adjusted mean change from baseline (95% CI)				
W12	−3.66 (−5.18 to −2.14)	−1.87 (−4.04 to 0.30)	−1.79 (−4.44 to 0.86)	
W24	−1.86 (−3.42 to −0.30)	−0.97 (−3.22 to 1.28)	−0.89 (−3.63 to 1.85)	
W36	−1.65 (−3.26 to −0.04)	−5.06 (−7.34 to −2.78)	3.41 (0.62 to 6.21)	
W48	−1.70 (−3.31 to −0.08)	−2.42 (−4.76 to −0.07)	0.72 (−2.13 to 3.56)	
W60	0.01 (−1.67 to 1.68)	−3.50 (−5.91 to −1.09)	3.50 (0.57 to 6.44)	
W72	0.42 (−1.28 to 2.13)	−1.63 (−4.07 to 0.81)	2.06 (−0.92 to 5.04)	
W84	0.04 (−1.72 to 1.80)	−2.66 (−5.17 to −0.15)	2.70 (−0.36 to 5.76)	
W96	−0.55 (−2.36 to 1.26)	−4.01 (−6.61 to −1.40)	3.46 (0.29 to 6.63)	
Physical functioning				
Baseline	N = 513	N = 252		
Mean (SD)	78.8 (17.6)	75.7 (19.1)		
Median (range)	80.0 (20-100)	80.0 (20-100)		
Adjusted mean global change from baseline (95% CI)	0.48 (−0.56 to 1.53)	0.38 (−1.11 to 1.87)	0.11 (−1.72 to 1.93)	*P*=.1318
Adjusted mean change from baseline (95% CI)				
W12	−1.52 (−2.90 to −0.15)	−0.28 (−2.23 to 1.67)	−0.24 (−3.63 to 1.15)	
W24	−0.05 (−1.45 to 1.35)	1.79 (−0.22 to 3.80)	−1.83 (−4.29 to 0.62)	
W36	−0.71 (−2.15 to 0.74)	−0.52 (−2.56 to 1.52)	−0.18 (−2.69 to 2.32)	
W48	−0.66 (−2.10 to 0.78)	−0.20 (−2.29 to 1.88)	−0.46 (−3.00 to 2.08)	
W60	0.75 (−0.75 to 2.25)	−0.60 (−2.75 to 1.54)	1.35 (−1.27 to 3.98)	

Scale scores	Olaparib plus bevacizumab (N = 517)	Placebo plus bevacizumab (N = 253)	Between-group difference	Treatment-by-time interaction
W72	1.79 (0.28 to 3.31)	1.23 (−0.93 to 3.39)	0.56 (−2.08 to 3.21)	
W84	1.22 (−0.34 to 2.79)	0.52 (−1.70 to 2.74)	0.70 (−2.03 to 3.43)	
W96	3.04 (1.44 to 4.64)	1.09 (−1.21 to 3.39)	1.95 (−0.86 to 4.76)	
Role functioning				
Baseline	N = 514	N = 252		
Mean (SD)	73.4 (27.2)	72.2 (27.0)		
Median (range)	83.3 (0-100)	66.7 (0-100)		
Adjusted mean global change from baseline (95% CI)	0.46 (−1.00 to 1.93)	−1.33 (−3.42 to 0.76)	1.79 (−0.76 to 4.35)	*P*=.0740
Adjusted mean change from baseline (95% CI)				
W12	−2.44 (−4.48 to −0.40)	−2.70 (−5.58 to 0.18)	0.26 (−3.27 to 3.79)	
W24	−0.19 (−2.27 to 1.88)	−0.09 (−3.08 to 2.90)	−0.10 (−3.74 to 3.53)	
W36	−1.44 (−3.59 to 0.70)	−2.92 (−5.95 to 0.12)	1.48 (−2.24 to 5.19)	
W48	−1.69 (−3.84 to 0.46)	−0.49 (−3.61 to 2.62)	−1.20 (−4.98 to 2.59)	
W60	2.30 (0.07 to 4.54)	−2.81 (−6.01 to 0.39)	5.11 (1.20 to 9.02)	
W72	1.38 (0.12 to 4.65)	−0.73 (−3.95 to 2.49)	3.11 (−0.82 to 7.02)	
W84	1.48 (−0.86 to 3.82)	−0.23 (−3.55 to 3.09)	1.71 (−2.35 to 5.78)	
W96	3.31 (0.90 to 5.72)	−0.67 (−4.12 to 2.78)	3.98 (−0.23 to 8.19)	
Emotional functioning				
Baseline	N = 515	N = 251		
Mean (SD)	76.9 (21.5)	77.2 (19.7)		
Median (range)	83.3 (0-100)	83.3 (0-100)		
Adjusted mean global change from baseline (95% CI)	−2.98 (−4.25 to −1.71)	−4.68 (−6.50 to −2.85)	1.69 (−0.53 to 3.92)	*P*=.4289
Adjusted mean change from baseline (95% CI)				
W12	−2.33 (−4.03 to −0.64)	−2.96 (−5.38 to −0.54)	0.63 (−2.33 to 3.58)	
W24	−1.89 (−3.61 to −0.16)	−2.69 (−5.19 to −0.19)	0.80 (−2.24 to 3.84)	
W36	−1.92 (−3.70 to −0.14)	−3.76 (−6.29 to −1.22)	1.83 (−1.26 to 4.93)	
W48	−3.06 (−4.85 to −1.26)	−3.42 (−6.02 to −0.82)	0.36 (−2.79 to 3.52)	
W60	−2.69 (−4.55 to −0.84)	−6.84 (−9.51 to −4.17)	4.15 (0.90 to 7.39)	

Scale scores	Olaparib plus bevacizumab (N = 517)	Placebo plus bevacizumab (N = 253)	Between-group difference	Treatment-by-time interaction
W72	−3.61 (−5.49 to −1.73)	−4.77 (−7.46 to −2.07)	1.16 (−2.13 to 4.44)	
W84	−3.83 (−5.78 to −1,89)	−5.68 (−8.44 to −2.91)	1.84 (−1.54 to 5.22)	
W96	−4.52 (−6.51 to −2.53)	−7.30 (−10.77 to −4.43)	2.78 (−0.71 to 6.27)	
Cognitive functioning				
Baseline	N = 515	N = 251		
Mean (SD)	82.6 (20.9)	81.4 (19.5)		
Median (range)	83.3 (0-100)	83.3 (0-100)		
Adjusted mean global change from baseline (95% CI)	−1.61 (−2.80 to −0.42)	−2.76 (−4.47 to −1.05)	1.15 (−0.93 to 3.24)	*P*=.9978
Adjusted mean change from baseline (95% CI)				
W12	−0.57 (−2.15 to 1.02)	−1.65 (−3.91 to 0.61)	1.09 (−1.68 to 3.85)	
W24	−0.34 (−1.96 to 1.28)	−2.27 (−4.61 to 0.07)	1.93 (−0.91 to 4.78)	
W36	−1.76 (−3.43 to −0.10)	−2.55 (−4.92 to −0.18)	0.79 (−2.11 to 3.68)	
W48	−1.60 (−3.27 to 0.08)	−2.55 (−4.97 to −0.12)	0.95 (−2.00 to 3.90)	
W60	−0.92 (−2.65 to 0.81)	−2.30 (−4.80 to 0.19)	1.38 (−1.65 to 4.42)	
W72	1.62 (−3.38 to 0.13)	−2.70 (−5.22 to −0.17)	1.07 (−2.00 to 4.15)	
W84	−3.05 (−4.86 to −1.23)	−4.23 (−6.81 to −1.64)	1.18 (−1.98 to 4.34)	
W96	−3.00 (−4.86 to −1.14)	−3.83 (−6.52 to −1.15)	0.84 (−2.43 to 4.10)	
Social functioning				
Baseline	N = 515	N = 251		
Mean (SD)	75.0 (26.9)	74.6 (25.3)		
Median (range)	83.3 (0-100)	83.3 (0-100)		
Adjusted mean global change from baseline (95% CI)	2.44 (0.99 to 3.89)	1.80 (−0.28 to 3.88)	0.64 (−1.89 to 3.18)	*P*=.5827
Adjusted mean change from baseline (95% CI)				
W12	1.69 (−0.31 to 3.68)	1.64 (−1.19 to 4.48)	0.04 (−3.42 to 3.51)	
W24	2.34 (0.31 to 4.37)	3.39 (0.46 to 6.33)	1.05 (−4.63 to 2.52)	
W36	1.96 (−0.14 to 4.05)	1.62 (−1.36 to 4.60)	0.34 (−3.30 to 3.98)	
W48	1.70 (−0.41 to 3.81)	2.74 (−0.32 to 5.80)	−1.04 (−4.76 to 2.67)	
W60	3.26 (1.07 to 5.44)	0.42 (−2.72 to 3.56)	2.84 (−0.99 to 6.66)	

Scale scores	Olaparib plus bevacizumab (N = 517)	Placebo plus bevacizumab (N = 253)	Between-group difference	Treatment-by-time interaction
W72	4.75 (2.53 to 6.97)	2.79 (−0.40 to 5.97)	1.96 (−1.92 to 5.84)	
W84	2.07 (−0.22 to 4.37)	0.94 (−2.33 to 4.20)	1.14 (−2.85 to 5.13)	
W96	1.76 (−0.59 to 4.11)	0.83 (−2.58 to 4.24)	0.92 (−3.22 to 5.07)	
Symptomatic scales				
Fatigue[a]				
Baseline	N = 513	N = 251		
Mean (SD)	33.7 (22.8)	34.5 (22.5)		
Median (range)	33.3 (0-100)	33.3 (0-100)		
Adjusted mean global change from baseline (95% CI)	1.61 (0.29 to 2.93)	1.93 (0.05 to 3.81)	−0.32 (−2.62 to 1.98)	*P*=.0006
Adjusted mean change from baseline (95% CI)				
W12	4.99 (3.18 to 6.81)	0.95 (−1.63 to 3.52)	4.05 (0.90 to 71.9)	
W24	2.54 (0.69 to 4.39)	0.53 (−2.13 to 3.91)	2.01 (−1.23 to 5.25)	
W36	2.90 (0.99 to 4.81)	4.23 (−1.53 to 6.93)	−1.33 (−4.64 to 1.98)	
W48	3.17 (1.26 to 5.09)	1.31 (−1.46 to 4.08)	1.86 (−1.50 to 5.23)	
W60	0.67 (−1.32 to 2.66)	2.90 (0.05 to 5.75)	−2.23 (−5.71 to 1.25)	
W72	−1.39 (−3.41 to 0.64)	1.63 (−1.23 to 4.49)	−3.02 (−6.52 to 0.49)	
W84	0.01 (−2.08 to 2.08)	2.28 (−0.67 to 5.23)	−2.28 (−5.89 to 1.33)	
W96	0.01 (−2.14 to 2.15)	1.62 (−1.46 to 4.70)	−1.62 (−5.38 to 2.14)	
Nausea and vomiting[a]				
Baseline	N = 514	N = 252		
Mean (SD)	4.6 (12.5)	3.6 (9.2)		
Median (range)	0.0 (0-100)	0.0 (0-50)		
Adjusted mean global change from baseline (95% CI)	6.24 (5.26 to 7.22)	3.02 (1.62 to 4.41)	3.22 (1.51 to 4.93)	*P*<.0001
Adjusted mean change from baseline (95% CI)				
W12	9.70 (8.24 to 11.6)	1.08 (−0.99 to 3.15)	8.62 (6.09 to 11.15)	
W24	6.71 (5.22 to 8.20)	1.17 (−0.98 to 3.32)	5.54 (2.93 to 8.16)	
W36	6.98 (5.43 to 8.52)	3.68 (1.49 to 5.87)	3.30 (0.62 to 5.98)	
W48	7.04 (5.49 to 8.59)	3.05 (1.81 to 6.30)	2.99 (0.26 to 5.73)	

Scale scores	Olaparib plus bevacizumab (N = 517)	Placebo plus bevacizumab (N = 253)	Between-group difference	Treatment-by-time interaction
W60	5.35 (3.74 to 6.97)	1.41 (0.08 to 4.73)	2.95 (0.12 to 5.79)	
W72	5.02 (3.37 to 6.67)	8.91 (0.58 to 5.24)	2.12 (−0.74 to 4.98)	
W84	4.48 (2.78 to 6.19)	2.19 (1.77 to 6.61)	0.30 (−2.66 to 3.26)	
W96	4.58 (2.82 to 6.34)	4.65 (2.13 to 7.17)	−0.07 (−3.14 to 3.01)	
Pain[a]				
Baseline	N = 516	N = 253		
Mean (SD)	22.4 (24.2)	23.8 (22.9)		
Median (range)	16.7 (0-100)	16.7 (0-100)		
Adjusted mean global change from baseline (95% CI)	3.48 (1.98 to 4.98)	7.33 (5.19 to 9.47)	−3.85 (−6.47 to −1.24)	*P*=.3168
Adjusted mean change from baseline (95% CI)				
W12	5.31 (3.21 to 7.41)	9.41 (6.43 to 12.39)	−4.10 (−7.75 to −0.46)	
W24	4.01 (1.86 to 6.15)	8.20 (5.11 to 11.29)	−4.19 (−7.96 to −0.43)	
W36	6.74 (4.53 to 8.96)	11.31 (8.17 to 14.44)	−4.56 (−8.40 to −0.72)	
W48	7.25 (5.03 to 9.47)	7.78 (4.56 to 11.00)	−0.53 (−4.44 to 3.39)	
W60	2.97 (0.66 to 5.28)	6.30 (2.97 to 9.62)	−3.33 (−7.37 to 0.72)	
W72	2.11 (−0.23 to 4.60)	4.82 (1.48 to 8.15)	−2.70 (−6.78 to 1.38)	
W84	−0.04 (−2.47 to 2.38)	6.76 (3.31 to 10.20)	−6.80 (−11.01 to −2.59)	
W96	−0.53 (−3.02 to 1.97)	4.07 (0.48 to 7.65)	−4.59 (−8.96 to −0.23)	
Dyspnea[a]				
Baseline	N = 509	N = 252		
Mean (SD)	23.1 (25.7)	23.0 (27.3)		
Median (range)	33.3 (0-100)	16.7 (0-100)		
Adjusted mean global change from baseline (95% CI)	0.25 (−1.28 to 1.78)	−0.13 (−2.30 to 2.04)	0.38 (−2.28 to 3.04)	*P*=.0796
Adjusted mean change from baseline (95% CI)				
W12	1.87 (−0.28 to 1.03)	−3.13 (−6.16 to −0.11)	5.01 (1.29 to 8.72)	
W24	−0.35 (−2.55 to 1.84)	−1.87 (−5.01 to 1.28)	1.51 (−2.32 to 5.35)	
W36	−0.84 (−3.12 to 1.44)	−0.06 (−3.25 to 3.13)	−0.78 (−4.70 to 3.14)	
W48	−0.01 (−2.28 to 2.26)	−0.29 (−3.57 to 2.99)	0.28 (−3.71 to 4.27)	

Scale scores	Olaparib plus bevacizumab (N = 517)	Placebo plus bevacizumab (N = 253)	Between-group difference	Treatment-by-time interaction
W60	0.58 (−1.78 to 2.95)	2.27 (−1.10 to 5.65)	−1.69 (−5.81 to 2.43)	
W72	−0.04 (−2.44 to 2.36)	−0.33 (−3.72 to 3.06)	0.29 (−3.87 to 4.44)	
W84	0.17 (−2.31 to 2.66)	0.42 (−3.07 to 3.91)	−0.25 (−4.53 to 4.04)	
W96	0.61 (−1.94 to 3.16)	1.95 (−1.70 to 5.60)	−1.34 (−5.79 to 3.11)	
Insomnia[a]				
Baseline	N = 512	N = 252		
Mean (SD)	27.0 (30.1)	24.6 (27.2)		
Median (range)	33.3 (0-100)	33.3 (0-100)		
Adjusted mean global change from baseline (95% CI)	2.81 (1.18 to 4.44)	6.10 (3.78 to 8.43)	−3.29 (−6.13 to −0.45)	*P*=.7097
Adjusted mean change from baseline (95% CI)				
W12	0.47 (−1.85 to 2.79)	5.69 (2.41 to 8.97)	−5.22 (−9.24 to −1.20)	
W24	3.28 (0.92 to 5.65)	5.00 (1.59 to 8.40)	−1.71 (−5.86 to 2.44)	
W36	3.97 (1.51 to 6.42)	6.17 (2.71 to 9.64)	−2.21 (−6.45 to 2.04)	
W48	2.97 (0.52 to 5.43)	4.62 (1.07 to 8.17)	−1.64 (−5.96 to 2.67)	
W60	4.05 (1.49 to 6.62)	7.71 (4.04 to 11.38)	−3.65 (−8.13 to 0.82)	
W72	1.01 (−1.59 to 3.62)	6.20 (2.52 to 9.88)	−5.18 (−9.69 to −0.68)	
W84	4.05 (1.36 to 6.74)	7.19 (3.39 to 10.98)	−3.14 (−7.79 to 1.52)	
W96	2.68 (−0.09 to 5.46)	6.25 (2.28 to 10.22)	−3.56 (−8.41 to 1.28)	
Appetite loss[a]				
Baseline	N = 513	N = 252		
Mean (SD)	8.3 (18.2)	9.7 (20.2)		
Median (range)	0.0 (0-100)	0.0 (0-100)		
Adjusted mean global change from baseline (95% CI)	5.24 (3.88 to 6.60)	3.84 (1.89 to 5.78)	1.40 (−0.97 to 3.77)	*P*<.0001
Adjusted mean change from baseline (95% CI)				
W12	8.47 (6.45 to 10.49)	0.36 (−2.50 to 3.22)	8.11 (4.61 to 11.61)	
W24	5.08 (3.01 to 7.15)	0.80 (−2.18 to 3.78)	4.28 (0.65 to 7.90)	
W36	7.25 (5.10 to 9.40)	3.87 (0.82 to 6.91)	3.38 (−0.34 to 7.11)	
W48	8.45 (6.30 to 10.60)	4.95 (1.85 to 8.06)	3.50 (−0.28 to 7.27)	

Scale scores	Olaparib plus bevacizumab (N = 517)	Placebo plus bevacizumab (N = 253)	Between-group difference	Treatment-by-time interaction
W60	3.56 (1.32 to 5.81)	6.48 (3.27 to 9.69)	−2.91 (−6.83 to 1.01)	
W72	3.38 (1.10 to 5.66)	2.42 (−0.81 to 5.64)	0.96 (−2.99 to 4.91)	
W84	1.63 (−0.73 to 3.99)	5.36 (2.02 to 8.71)	−3.74 (−7.83 to 0.36)	
W96	4.08 (1.64 to 6.52)	6.47 (2.98 to 9.95)	−2.38 (−6.64 to 1.87)	
Constipation[a]				
Baseline	N = 512	N = 250		
Mean (SD)	17.8 (26.8)	17.6 (27.6)		
Median (range)	0.0 (0-100)	0.0 (0-100)		
Adjusted mean global change from baseline (95% CI)	0.61 (−0.85 to 2.06)	1.72 (−0.36 to 3.79)	−1.11 (−3.65 to 1.42)	*P*=.2833
Adjusted mean change from baseline (95% CI)				
W12	−2.31 (−4.45 to −0.16)	−4.49 (−7.53 to −1.46)	2.19 (−1.53 to 5.90)	
W24	−2.06 (−4.25 to 0.13)	−0.57 (−3.75 to 2.61)	−1.49 (−5.35 to 2.37)	
W36	0.14 (−2.13 to 2.41)	1.00 (−2.20 to 4.21)	−0.86 (−4.79 to 3.07)	
W48	−0.59 (−2.87 to 1.69)	1.05 (−2.24 to 4.34)	−1.64 (−5.65 to 2.36)	
W60	1.64 (−0.75 to 4.02)	2.52 (−0.88 to 5.92)	−0.88 (−5.04 to 3.27)	
W72	1.27 (−1.15 to 3.70)	2.99 (−0.43 to 6.42)	−1.72 (−5.92 to 2.48)	
W84	2.74 (0.23 to 5.24)	7.38 (3.83 to 10.92)	−4.64 (−8.98 to −0.30)	
W96	4.03 (1.45 to 6.61)	3.88 (0.19 to 7.57)	0.15 (−4.35 to 4.65)	
Diarrhea[a]				
Baseline	N = 510	N = 252		
Mean (SD)	10.1 (20.3)	13.1 (23.6)		
Median (range)	0.0 (0-100)	0.0 (0-100)		
Adjusted mean global change from baseline (95% CI)	−0.59 (−1.77 to 0.58)	1.50 (−0.18 to 3.17)	−2.09 (−4.14 to −0.04)	*P*=.1862
Adjusted mean change from baseline (95% CI)				
W12	1.74 (−0.06 to 3.53)	0.36 (−2.18 to 2.89)	1.38 (−1.73 to 4.49)	
W24	0.30 (−1.54 to 2.13)	1.83 (−0.82 to 4.47)	−1.53 (−4.76 to 1.70)	
W36	0.05 (−1.85 to 1.96)	3.59 (0.89 to 6.30)	−3.54 (−6.85 to −0.23)	
W48	−0.86 (−2.78 to 1.05)	3.25 (0.48 to 6.02)	−4.11 (−7.48 to −0.74)	

Scale scores	Olaparib plus bevacizumab (N = 517)	Placebo plus bevacizumab (N = 253)	Between-group difference	Treatment-by-time interaction
W60	−1.19 (−3.19 to 0.82)	1.64 (−1.22 to 4.51)	−2.83 (−6.33 to 0.68)	
W72	−1.67 (−3.71 to 0.38)	0.13 (−2.76 to 3.03)	−1.80 (−5.36 to 1.76)	
W84	−2.43 (−4.55 to −0.30)	−0.67 (−3.67 to 2.34)	−1.76 (−5.45 to 1.92)	
W96	−0.69 (−2.87 to 1.49)	1.86 (−1.27 to 4.98)	−2.54 (−6.37 to 1.28)	
Financial difficulties[a]				
Baseline	N = 508	N = 246		
Mean (SD)	14.6 (27.6)	16.3 (28.0)		
Median (range)	0.0 (0-100)	0.0 (0-100)		
Adjusted mean global change from baseline (95% CI)	−1.40 (−2.74 to −0.06)	−0.69 (−2.61 to 1.24)	−0.72 (−3.06 to 1.63)	*P*=.9181
Adjusted mean change from baseline (95% CI)				
W12	−1.01 (−2.78 to 0.76)	−0.33 (−2.84 to 2.17)	1.38 (−1.73 to 4.49)	
W24	−1.67 (−3.46 to 0.13)	−1.46 (−4.06 to 1.13)	−0.20 (−3.36 to 2.95)	
W36	−1.14 (−2.99 to 0.70)	−1.57 (−4.20 to 1.06)	0.42 (−2.79 to 3.64)	
W48	−1.31 (−3.16 to 0.55)	−0.83 (−3.51 to 1.84)	−0.47 (−3.73 to 2.79)	
W60	−1.52 (−3.44 to 0.40)	−0.24 (−3.00 to 2.52)	−2.83 (−6.33 to 0.68)	
W72	−2.07 (−4.02 to −0.12)	−0.10 (−2.67 to 2.88)	−1.80 (−5.36 to 1.76)	
W84	−1.11 (−3.11 to 0.90)	−0.06 (−2.92 to 2.80)	−1.76 (−5.45 to 1.92)	
W96	−1.38 (−3.43 to 0.67)	−1.08 (−4.07 to 1.90)	−2.54 (−6.37 to 1.28)	

Longitudinal analysis of HRQoL data was conducted using a mixed model for repeated measures until 2 years of follow-up for each HRQoL scale separately. When time-by-treatment interactions are significant, time-specific estimates may be more relevant than estimates of mean global change.

[a]Symptomatic scale where higher scores represent higher symptomatic burden. For all other HRQoL scales, higher scores represent higher QoL level.

Abbreviations: CI = confidence interval; EORTC QLQ-C30 = European Organisation for Research and Treatment of Cancer Core Quality of Life Questionnaire; HRQoL = health-related quality of life; ITT = intention-to-treat; QoL = quality of life; SD = standard deviation; W = Week.

Table S5. Baseline EORTC QLQ-OV28 scores and adjusted mean change from baseline in the modified ITT population

Scale scores	Olaparib plus bevacizumab (N = 517)	Placebo plus bevacizumab (N = 253)	Between-group difference	Treatment-by-time interaction
Functional scales				
Body image				
Baseline	N = 514	N = 245		
Mean (SD)	64.3 (30.6)	60.9 (32.2)		
Median (range)	66.7 (0-100)	66.7 (0-100)		
Adjusted mean global change from baseline (95% CI)	8.05 (6.36 to 9.74)	5.91 (3.46 to 8.35)	2.14 (−0.83 to 5.11)	*P*=.0200
Adjusted mean change from baseline (95% CI)				
W12	5.48 (3.29 to 7.67)	6.28 (3.13 to 9.43)	−0.80 (−4.63 to 3.04)	
W24	7.90 (5.67 to 10.13)	6.59 (3.35 to 9.82)	1.31 (−2.62 to 5.25)	
W36	6.75 (4.46 to 9.04)	6.80 (3.49 to 10.11)	−0.05 (−4.08 to 3.98)	
W48	7.97 (5.67 to 10.27)	7.60 (4.25 to 10.96)	0.37 (−3.71 to 4.44)	
W60	9.49 (7.12 to 11.87)	4.06 (0.61 to 7.51)	5.43 (1.24 to 9.62)	
W72	8.23 (5.82 to 10.64)	6.20 (2.75 to 9.66)	2.03 (−2.19 to 6.24)	
W84	9.75 (7.27 to 12.22)	6.88 (3.32 to 10.44)	2.87 (−1.47 to 7.20)	
W96	8.83 (6.30 to 11.37)	2.84 (−0.85 to 6.52)	6.00 (1.52 to 10.47)	
Sexual function				
Baseline	N = 489	N = 238		
Mean (SD)	13.6 (21.0)	14.0 (21.3)		
Median (range)	0.0 (0-100)	0.0 (0-100)		
Adjusted mean global change from baseline (95% CI)	1.42 (0.10 to 2.73)	4.14 (2.27 to 6.01)	−2.73 (−5.01 to −0.44)	*P*=.2813
Adjusted mean change from baseline (95% CI)				
W12	1.72 (0.01 to 3.44)	4.82 (2.39 to 7.26)	−3.10 (−6.08 to −0.13)	
W24	2.01 (0.25 to 3.78)	6.19 (3.67 to 8.72)	−4.18 (−7.26 to −1.09)	
W36	1.13 (−0.70 to 2.96)	5.37 (2.77 to 7.97)	−4.24 (7.42 to −1.06)	
W48	0.04 (−1.80 to 1.88)	4.05 (1.41 to 6.69)	−4.01 (−7.23 to −0.79)	
W60	0.93 (−0.98 to 2.84)	2.65 (−0.05 to 5.35)	−1.72 (−5.03 to 1.59)	

Scale scores	Olaparib plus bevacizumab (N = 517)	Placebo plus bevacizumab (N = 253)	Between-group difference	Treatment-by-time interaction
W72	1.96 (0.03 to 3.89)	2.31 (−0.42 to 5.04)	−0.34 (−3.69 to 3.00)	
W84	2.13 (0.13 to 4.13)	3.66 (0.85 to 6.48)	−1.53 (−4.99 to 1.92)	
W96	1.41 (−0.63 to 3.45)	4.08 (1.19 to 6.97)	−2.67 (−6.21 to 0.86)	
Attitude to disease/treatment				
Baseline	N = 511	N = 246		
Mean (SD)	43.7 (27.0)	42.7 (27.4)		
Median (range)	44.4 (0-100)	44.4 (0-100)		
Adjusted mean global change from baseline (95% CI)	10.18 (8.63 to 11.73)	8.58 (6.34 to 10.82)	1.60 (−1.13 to 4.32)	*P*=.0003
Adjusted mean change from baseline (95% CI)				
W12	4.83 (2.76 to 6.91)	8.66 (5.68 to 11.64)	−3.82 (−7.45 to −0.19)	
W24	7.51 (5.39 to 9.63)	8.27 (5.21 to 11.34)	−0.76 (−4.49 to 2.96)	
W36	8.44 (4.26 to 10.62)	7.43 (4.29 to 10.56)	1.01 (−2.80 to 4.83)	
W48	10.18 (1.99 to 12.36)	9.59 (6.40 to 12.79)	0.58 (−3.29 to 4.46)	
W60	12.68 (6.42 to 14.93)	7.53 (4.25 to 10.82)	5.14 (1.16 to 9.13)	
W72	12.34 (3.04 to 14.64)	9.07 (5.78 to 12.35)	3.27 (−0.74 to 7.28)	
W84	12.44 (4.07 to 14.81)	10.09 (6.69 to 13.49)	2.35 (−1.80 to 6.49)	
W96	13.02 (0.59 to 15.45)	8.01 (4.47 to 11.54)	5.01 (0.72 to 9.30)	
Symptomatic scales				
Abdominal/GI symptoms[a]				
Baseline	N = 517	N = 247		
Mean (SD)	19.5 (17.4)	19.8 (16.8)		
Median (range)	14.3 (0-90)	14.3 (0-86)		
Adjusted mean global change from baseline (95% CI)	2.95 (1.92 to 3.99)	4.82 (3.32 to 6.33)	−1.87 (−3.70 to −0.04)	*P*=.3106
Adjusted mean change from baseline (95% CI)				
W12	1.93 (0.52 to 3.35)	1.35 (−0.69 to 3.40)	0.58 (−1.90 to 3.06)	
W24	1.57 (0.13 to 3.02)	3.34 (1.24 to 5.43)	−1.76 (−4.31 to 0.78)	
W36	3.81 (2.31 to 5.30)	5.25 (3.12 to 7.39)	−1.45 (−4.05 to 1.16)	
W48	3.14 (1.64 to 4.63)	4.81 (2.63 to 6.99)	−1.67 (−4.32 to 0.97)	

Scale scores	Olaparib plus bevacizumab (N = 517)	Placebo plus bevacizumab (N = 253)	Between-group difference	Treatment-by-time interaction
W60	2.97 (1.42 to 4.52)	5.52 (3.27 to 7.76)	−2.55 (−5.28 to 0.18)	
W72	2.65 (1.07 to 4.22)	4.95 (2.68 to 7.22)	−2.30 (−5.07 to 0.46)	
W84	2.91 (1.29 to 4.53)	5.66 (3.33 to 7.98)	−2.75 (−5.58 to 0.09)	
W96	4.66 (2.99 to 6.33)	7.71 (5.30 to 10.12)	−3.06 (−5.99 to −0.12)	
Peripheral neuropathy[a]				
Baseline	N = 517	N = 246		
Mean (SD)	41.8 (29.8)	44.4 (30.3)		
Median (range)	33.3 (0-100)	44.4 (0-100)		
Adjusted mean global change from baseline (95% CI)	−13.77 (−15.21 to −12.32)	−12.32 (−14.41 to −10.23)	−1.45 (−3.99 to 1.10)	*P*=.1894
Adjusted mean change from baseline (95% CI)				
W12	−7.42 (−9.28 to −5.55)	−7.00 (−9.70 to −4.29)	−0.42 (−3.70 to 2.86)	
W24	−12.87 (−14.78 to −10.97)	−8.65 (−11.41 to −5.88)	−4.23 (−7.59 to −0.87)	
W36	−12.36 (−14.32 to −10.39)	−11.84 (−14.65 to −9.02)	−0.52 (−3.96 to 2.91)	
W48	−14.12 (−16.08 to −12.16)	−14.41 (−17.28 to −11.54)	0.29 (−3.18 to 3.77)	
W60	−14.46 (−16.49 to −12.42)	−13.16 (−16.11 to −10.22)	−1.29 (−4.87 to 2.29)	
W72	−16.73 (−18.78 to −14.68)	−14.71 (−17.68 to −11.75)	−2.02 (−5.63 to 1.60)	
W84	−16.62 (−18.73 to −14.51)	−13.66 (−16.70 to −10.61)	−2.96 (−6.67 to 0.74)	
W96	−15.57 (−17.75 to −13.39)	−15.14 (−18.27 to −12.01)	−0.43 (−4.25 to 3.39)	
Hormonal/menopausal symptoms[a]				
Baseline	N = 516	N = 245		
Mean (SD)	22.5 (28.3)	22.6 (29.0)		
Median (range)	16.7 (0-100)	16.7 (0-100)		
Adjusted mean global change from baseline (95% CI)	−2.45 (−3.88 to −1.01)	0.87 (−1.22 to 2.96)	−3.31 (−5.85 to −0.78)	*P*=.1145
Adjusted mean change from baseline (95% CI)				
W12	−4.65 (−6.58 to −2.72)	1.32 (−1.47 to 4.12)	−5.97 (−9.37 to −2.58)	
W24	−5.01 (−6.99 to −3.04)	1.29 (−1.58 to 4.16)	−6.30 (−9.78 to −2.82)	
W36	−3.20 (−5.23 to −1.17)	−0.97 (−3.88 to 1.94)	−2.23 (−5.78 to 1.32)	
W48	−3.56 (−5.59 to −1.52)	−0.95 (−3.94 to 2.03)	−2.60 (−6.21 to 1.01)	

Scale scores	Olaparib plus bevacizumab (N = 517)	Placebo plus bevacizumab (N = 253)	Between-group difference	Treatment-by-time interaction
W60	−2.60 (−4.71 to −0.50)	0.32 (−2.74 to 3.38)	−2.93 (−6.64 to 0.79)	
W72	−0.82 (−2.95 to 1.32)	1.80 (−1.27 to 4.88)	−2.62 (−6.37 to 1.12)	
W84	−1.09 (−1.12 to 3.29)	2.56 (−0.61 to 5.73)	−1.48 (−5.34 to 2.39)	
W96	−0.81 (−3.08 to 1.46)	1.58 (−1.70 to 4.87)	−2.39 (−6.38 to 1.60)	
Other chemotherapy side effects[a]				
Baseline	N = 517	N = 246		
Mean (SD)	23.7 (16.9)	23.3 (17.6)		
Median (range)	20.0 (0-73)	20.0 (0-93)		
Adjusted mean global change from baseline (95% CI)	−3.44 (−4.34 to −2.54)	−2.33 (−3.63 to −1.02)	−1.11 (−2.70 to 0.47)	*P*=.1139
Adjusted mean change from baseline (95% CI)				
W12	−0.36 (−1.57 to 0.85)	−1.13 (−2.88 to 0.63)	0.76 (−1.36 to 2.89)	
W24	−1.54 (−2.77 to −0.30)	−0.65 (−2.44 to 1.15)	−0.89 (−3.07 to 1.29)	
W36	−1.51 (−2.79 to −0.23)	−1.05 (−2.89 to 0.78)	−0.46 (−2.69 to 1.77)	
W48	−2.58 (−3.86 to −1.30)	−1.32 (−3.19 to 0.55)	−1.26 (−3.53 to 1.01)	
W60	−5.00 (−6.32 to −3.67)	−2.72 (−4.64 to −0.79)	−2.28 (−4.62 to 0.06)	
W72	−5.28 (−6.62 to −3.93)	−4.14 (−6.08 to −2.20)	−1.14 (−3.50 to 1.22)	
W84	−6.33 (−7.71 to −4.94)	−3.23 (−5.23 to −1.24)	−3.09 (−5.52 to −0.67)	
W96	−4.94 (−6.37 to −3.51)	−4.39 (−6.44 to −2.33)	−0.56 (−3.06 to 1.94)	
Hair loss[a]				
Baseline	N = 502	N = 244		
Mean (SD)	32.2 (37.9)	31.6 (39.6)		
Median (range)	0.0 (0-100)	0.0 (0-100)		
Adjusted mean global change from baseline (95% CI)	−21.92 (−23.18 to −20.65)	−21.38 (−23.19 to −19.57)	−0.53 (−2.74 to 1.67)	*P*=.4264
Adjusted mean change from baseline (95% CI)				
W12	−24.77 (−26.74 to −22.81)	−23.77 (−26.57 to −20.97)	−1.01 (−4.43 to 2.42)	
W24	−28.05 (−30.07 to −26.03)	−24.80 (−27.70 to −21.90)	−3.24 (−6.78 to 0.29)	
W36	−27.20 (−29.29 to −25.10)	−25.35 (−28.30 to −22.39)	−1.85 (−5.48 to 1.77)	
W48	−24.35 (−26.44 to −22.26)	−25.39 (−28.43 to −22.36)	1.04 (−2.65 to 4.72)	

Scale scores	Olaparib plus bevacizumab (N = 517)	Placebo plus bevacizumab (N = 253)	Between-group difference	Treatment-by-time interaction
W60	−19.93 (−22.13 to −17.73)	−18.66 (−21.82 to −15.50)	−1.27 (−5.12 to 2.58)	
W72	−17.53 (−19.75 to −15.30)	−16.80 (−19.98 to −13.62)	−0.73 (−4.61 to 3.16)	
W84	−16.78 (−19.09 to −14.47)	−17.97 (−21.25 to −14.70)	1.19 (−2.82 to 5.21)	
W96	−16.73 (−19.13 to −14.32)	−18.31 (−21.72 to −14.89)	1.58 (−2.59 to 5.75)	

Longitudinal analysis of HRQoL data was conducted using a mixed model for repeated measures until 2 years of follow-up for each HRQoL scale separately. When time-by-treatment interactions are significant, time-specific estimates may be more relevant than estimates of mean global change.

[a]Symptomatic scale where higher scores represent higher symptomatic burden. For all other HRQoL scales, higher scores represent higher QoL level.

CI = confidence interval; EORTC QLQ-OV28 = European Organisation for Research and Treatment of Cancer Quality of Life Questionnaire Ovarian Cancer module; GI = gastrointestinal; HRQoL = health-related quality of life; ITT = intention-to-treat; QoL = quality of life; SD = standard deviation; W = Week.

Table S6. Baseline EORTC QLQ-C30 scores and adjusted mean change from baseline in the HRD-positive subgroup

Scale scores	Olaparib plus bevacizumab (N = 249)	Placebo plus bevacizumab (N = 127)	Between-group difference	Treatment-by-time interaction
Functional scales				
Global Health Status				
Baseline	N = 247	N = 125		
Mean (SD)	69.4 (17.4)	68.7 (15.8)		
Median (range)	66.7 (17-100)	66.7 (33-100)		
Adjusted mean global change from baseline (95% CI)	−0.09 (−1.51 to 1.32)	−1.32 (−3.36 to 0.71)	1.23 (−1.25 to 3.71)	*P*=.0076
Adjusted mean change from baseline (95% CI)				
W12	−4.01 (−6.05 to −1.97)	−1.60 (−4.47 to 1.27)	−2.41 (−5.93 to 1.11)	
W24	−1.37 (−3.44 to 0.69)	0.07 (−2.90 to 3.05)	−1.45 (−5.07 to 2.18)	
W36	−0.18 (−2.28 to 1.92)	−2.01 (−5.02 to 1.00)	1.83 (−1.84 to 5.50)	
W48	−1.43 (−3.54 to 0.67)	−1.68 (−4.76 to 1.40)	0.24 (−3.49 to 3.97)	
W60	0.73 (−1.45 to 2.90)	0.28 (−2.89 to 3.44)	0.45 (−3.39 to 4.29)	
W72	1.88 (−0.31 to 4.06)	0.42 (−2.74 to 3.59)	1.45 (−2.40 to 5.30)	
W84	2.56 (0.32 to 4.81)	−2.17 (−5.50 to 1.16)	4.74 (0.72 to 8.75)	
W96	1.09 (−1.19 to 3.37)	−3.90 (−7.24 to −0.56)	4.99 (0.94 to 9.04)	
Physical functioning				
Baseline	N = 247	N = 127		
Mean (SD)	79.4 (16.9)	76.6 (18.6)		
Median (range)	80.0 (20-100)	80.0 (20-100)		
Adjusted mean global change from baseline (95% CI)	1.61 (0.14 to 3.07)	0.40 (−1.69 to 2.48)	1.21 (−1.34 to 3.76)	*P*=.0023
Adjusted mean change from baseline (95% CI)				
W12	−1.23 (−3.12 to 0.66)	−0.77 (−3.41 to 1.87)	−0.46 (−3.72 to 2.79)	
W24	0.25 (−1.65 to 2.15)	1.93 (−0.80 to 4.66)	−1.68 (−5.01 to 1.65)	
W36	0.34 (−1.60 to 2.28)	0.58 (−2.17 to 3.33)	−0.23 (−3.60 to 3.14)	
W48	−0.23 (−2.17 to 1.70)	−0.34 (−3.14 to 2.46)	0.11 (−3.30 to 3.51)	
W60	1.57 (−0.42 to 3.57)	1.24 (−1.63 to 4.12)	0.33 (−3.17 to 3.84)	
W72	3.22 (1.22 to 5.23)	0.61 (−2.26 to 3.47)	2.62 (−0.88 to 6.12)	

Scale scores	Olaparib plus bevacizumab (N = 249)	Placebo plus bevacizumab (N = 127)	Between-group difference	Treatment-by-time interaction
W84	4.06 (2.02 to 6.11)	−0.15 (−3.13 to 2.82)	4.22 (0.60 to 7.83)	
W96	4.89 (2.82 to 6.95)	0.08 (−2.93 to 3.08)	4.81 (1.16 to 8.46)	
Role functioning				
Baseline	N = 247	N = 127		
Mean (SD)	79.4 (26.6)	76.6 (18.6)		
Median (range)	80.0 (0-100)	80.0 (20-100)		
Adjusted mean global change from baseline (95% CI)	2.13 (0.16 to 4.11)	−0.38 (−3.20 to 2.44)	2.52 (−0.93 to 5.96)	*P*=.0305
Adjusted mean change from baseline (95% CI)				
W12	−3.89 (−6.69 to −1.08)	−3.36 (−7.27 to 0.54)	−0.53 (−5.34 to 4.28)	
W24	1.59 (−1.24 to 4.41)	0.05 (−4.01 to 4.10)	1.54 (−3.40 to 6.48)	
W36	0.81 (−2.07 to 3.70)	−0.88 (−4.98 to 3.22)	1.70 (−3.31 to 6.71)	
W48	−1.07 (−3.94 to 1.81)	1.31 (−2.88 to 5.50)	−2.37 (−7.46 to 2.71)	
W60	3.14 (0.16 to 6.13)	0.32 (−4.00 to 4.64)	2.82 (−2.43 to 8.07)	
W72	4.61 (1.61 to 7.60)	1.18 (−3.10 to 5.47)	3.42 (−1.80 to 8.65)	
W84	5.64 (2.57 to 8.71)	−0.74 (−5.23 to 3.75)	6.38 (0.94 to 11.81)	
W96	6.24 (3.12 to 9.35)	−0.94 (−5.49 to 3.61)	7.17 (1.66 to 12.69)	
Emotional functioning				
Baseline	N = 248	N = 125		
Mean (SD)	76.2 (22.1)	77.6 (18.2)		
Median (range)	83.3 (0-100)	83.3 (17-100)		
Adjusted mean global change from baseline (95% CI)	−2.60 (−4.35 to −0.86)	−4.16 (−6.67 to −1.65)	1.56 (−1.50 to 4.62)	*P*=.6061
Adjusted mean change from baseline (95% CI)				
W12	−2.25 (−4.63 to 0.13)	−3.76 (−7.13 to −0.40)	1.51 (−2.60 to 5.63)	
W24	−1.93 (−4.33 to 0.46)	−3.13 (−6.61 to 0.34)	1.20 (−3.02 to 5.42)	
W36	−1.32 (−3.76 to 1.12)	−2.85 (−6.36 to 0.67)	1.53 (−2.75 to 5.80)	
W48	−4.05 (−6.49 to −1.61)	−3.56 (−7.15 to 0.03)	−0.49 (−4.83 to 3.85)	
W60	−2.46 (−4.98 to 0.06)	−5.94 (−9.62 to −2.27)	3.48 (−0.98 to 7.94)	
W72	−3.44 (−5.98 to −0.91)	−2.78 (−6.46 to 0.90)	−0.66 (−5.13 to 3.80)	

Scale scores	Olaparib plus bevacizumab (N = 249)	Placebo plus bevacizumab (N = 127)	Between-group difference	Treatment-by-time interaction
W84	−1.85 (−4.46 to 0.76)	−4.72 (−8.55 to −0.88)	2.87 (−1.77 to 7.51)	
W96	−3.53 (−6.16 to −0.89)	−6.57 (−10.44 to −2.70)	3.04 (−1.64 to 7.72)	
Cognitive functioning				
Baseline	N = 248	N = 125		
Mean (SD)	81.5 (20.4)	81.5 (19.0)		
Median (range)	83.3 (0-100)	83.3 (17-100)		
Adjusted mean global change from baseline (95% CI)	−1.62 (−3.25 to 0.02)	−1.83 (−4.19 to 0.52)	0.22 (−2.65 to 3.09)	*P*=.8898
Adjusted mean change from baseline (95% CI)				
W12	−1.03 (−3.26 to 1.21)	−2.30 (−5.46 to 0.86)	1.27 (−2.60 to 5.14)	
W24	0.33 (−1.93 to 2.58)	−1.69 (−4.96 to 1.58)	2.02 (−1.95 to 5.99)	
W36	−2.05 (−4.35 to 0.24)	−2.36 (−5.67 to 0.95)	0.31 (−3.72 to 4.33)	
W48	−2.28 (−4.58 to 0.02)	−2.32 (−5.70 to 1.05)	0.04 (−4.04 to 4.12)	
W60	−0.95 (−3.32 to 1.42)	−0.69 (−4.15 to 2.77)	−0.26 (−4.46 to 3.94)	
W72	−1.57 (−3.96 to 0.81)	−0.99 (−4.45 to 2.47)	−0.58 (−4.78 to 3.62)	
W84	−2.21 (−4.66 to 0.24)	−0.86 (−4.46 to 2.75)	−1.36 (−5.71 to 3.00)	
W96	−3.15 (−5.63 to −0.67)	−3.45 (−7.08 to 0.19)	0.30 (−4.10 to 4.70)	
Social functioning				
Baseline	N = 248	N = 125		
Mean (SD)	74.1 (27.8)	73.6 (24.6)		
Median (range)	83.3 (0-100)	66.7 (0-100)		
Adjusted mean global change from baseline (95% CI)	4.64 (2.75 to 6.53)	3.48 (0.76 to 6.20)	1.16 (−2.16 to 4.48)	*P*=.3111
Adjusted mean change from baseline (95% CI)				
W12	1.83 (−0.87 to 4.53)	2.72 (−1.09 to 6.53)	−0.89 (−5.56 to 3.79)	
W24	3.36 (0.64 to 6.09)	4.11 (0.16 to 8.06)	−0.75 (−5.55 to 4.05)	
W36	4.71 (1.93 to 7.48)	3.53 (−0.47 to 7.53)	1.18 (−3.69 to 6.04)	
W48	2.07 (−0.71 to 4.86)	3.93 (−0.16 to 8.02)	−1.86 (−6.80 to 3.09)	
W60	6.09 (3.22 to 8.97)	5.37 (1.17 to 9.57)	0.72 (−4.37 to 5.81)	
W72	8.53 (5.63 to 11.42)	4.63 (0.43 to 8.84)	3.89 (−1.21 to 9.00)	

Scale scores	Olaparib plus bevacizumab (N = 249)	Placebo plus bevacizumab (N = 127)	Between-group difference	Treatment-by-time interaction
W84	4.85 (1.87 to 7.82)	0.81 (−3.58 to 5.20)	4.04 (−1.27 to 9.34)	
W96	5.68 (2.67 to 8.70)	2.73 (−1.70 to 7.17)	2.95 (−2.41 to 8.31)	
Symptomatic scales				
Fatigue[a]				
Baseline	N = 246	N = 126		
Mean (SD)	32.9 (23.1)	34.6 (22.3)		
Median (range)	33.3 (0-100)	33.3 (0-100)		
Adjusted mean global change from baseline (95% CI)	1.07 (−0.80 to 2.93)	1.93 (−0.73 to 4.59)	−0.86 (−4.11 to 2.39)	*P*=.0064
Adjusted mean change from baseline (95% CI)				
W12	6.30 (3.74 to 8.86)	3.14 (−0.44 to 6.71)	3.17 (−1.23 to 7.56)	
W24	1.95 (−0.63 to 4.52)	0.56 (−3.15 to 4.26)	1.39 (−3.13 to 5.90)	
W36	1.65 (−0.98 to 4.28)	2.74 (−0.99 to 6.47)	−1.10 (−5.66 to 3.47)	
W48	3.45 (0.83 to 6.07)	0.88 (−2.92 to 4.69)	2.57 (−2.05 to 7.20)	
W60	0.60 (−2.11 to 3.31)	0.75 (−3.17 to 4.67)	−0.15 (−4.92 to 4.62)	
W72	−1.93 (−4.65 to 0.80)	2.57 (−1.32 to 6.46)	−4.50 (−9.25 to 0.26)	
W84	−1.44 (−4.22 to 1.34)	2.82 (−1.25 to 6.88)	−4.26 (−9.18 to 0.67)	
W96	−2.03 (−4.86 to 0.79)	1.98 (−2.13 to 6.10)	−4.02 (−9.01 to 0.98)	
Nausea and vomiting[a]				
Baseline	N = 247	N = 127		
Mean (SD)	4.3 (11.7)	3.1 (8.8)		
Median (range)	0.0 (0-100)	0.0 (0-50)		
Adjusted mean global change from baseline (95% CI)	6.01 (4.64 to 7.37)	2.33 (0.38 to 4.28)	3.68 (1.30 to 6.06)	*P*<.0001
Adjusted mean change from baseline (95% CI)				
W12	10.30 (8.35 to 12.26)	1.24 (−1.49 to 3.97)	9.06 (5.71 to 12.42)	
W24	6.16 (4.19 to 8.13)	−0.47 (−3.31 to 2.36)	6.63 (3.18 to 10.08)	
W36	5.20 (3.18 to 7.21)	1.64 (−1.22 to 4.50)	3.56 (0.06 to 7.06)	
W48	7.66 (5.65 to 9.67)	3.26 (0.33 to 6.19)	4.40 (0.84 to 7.95)	
W60	6.09 (4.00 to 8.17)	1.01 (−2.01 to 4.03)	5.08 (1.41 to 8.75)	

Scale scores	Olaparib plus bevacizumab (N = 249)	Placebo plus bevacizumab (N = 127)	Between-group difference	Treatment-by-time interaction
W72	5.10 (3.00 to 7.20)	2.56 (−0.44 to 5.56)	2.54 (−1.13 to 6.20)	
W84	4.26 (2.12 to 6.41)	4.10 (0.95 to 7.24)	0.17 (−3.65 to 3.98)	
W96	3.30 (1.12 to 5.48)	5.29 (2.11 to 8.48)	−1.99 (−5.86 to 1.87)	
Pain[a]				
Baseline	N = 249	N = 127		
Mean (SD)	22.6 (23.3)	23.8 (23.0)		
Median (range)	16.7 (0-100)	16.7 (0-100)		
Adjusted mean global change from baseline (95% CI)	4.15 (2.05 to 6.25)	6.36 (3.36 to 9.36)	−2.21 (−5.87 to 1.45)	*P*=.1616
Adjusted mean change from baseline (95% CI)				
W12	7.71 (4.78 to 10.64)	9.53 (5.44 to 13.63)	−1.82 (−6.86 to 3.21)	
W24	5.27 (2.32 to 8.23)	6.66 (2.43 to 10.90)	−1.39 (−6.56 to 3.78)	
W36	7.32 (4.31 to 10.33)	8.58 (4.28 to 12.87)	−1.26 (−6.50 to 3.99)	
W48	7.85 (4.84 to 10.86)	6.50 (2.11 to 10.89)	1.35 (−3.97 to 6.67)	
W60	3.48 (0.38 to 6.58)	5.11 (0.59 to 9.63)	−1.63 (−7.11 to 3.85)	
W72	2.69 (−0.42 to 5.81)	3.56 (−0.93 to 8.04)	−0.86 (−6.32 to 4.60)	
W84	−0.88 (−4.08 to 2.32)	7.18 (2.49 to 11.88)	−8.06 (−13.74 to −2.38)	
W96	−0.25 (−3.50 to 3.01)	3.77 (−0.99 to 8.52)	−4.01 (−9.78 to 1.75)	
Dyspnea[a]				
Baseline	N = 244	N = 127		
Mean (SD)	23.8 (26.7)	23.4 (27.0)		
Median (range)	33.3 (0-100)	33.3 (0-100)		
Adjusted mean global change from baseline (95% CI)	−1.15 (−3.31 to 1.00)	−1.15 (−4.20 to 1.91)	−0.01 (−3.74 to 3.73)	*P*=.2853
Adjusted mean change from baseline (95% CI)				
W12	2.21 (−0.82 to 5.23)	−2.30 (−6.48 to 1.88)	4.51 (−0.65 to 9.67)	
W24	−1.55 (−4.60 to 1.50)	−1.59 (−5.93 to 2.74)	0.04 (−5.26 to 5.34)	
W36	−2.83 (−5.93 to 0.27)	−1.09 (−5.47 to 3.30)	−1.74 (−7.11 to 3.63)	
W48	−0.70 (−3.79 to 2.39)	−2.55 (−7.03 to 1.93)	1.85 (−3.59 to 7.29)	
W60	−0.52 (−3.72 to 2.69)	0.49 (−4.12 to 5.10)	−1.01 (−6.63 to 4.60)	

Scale scores	Olaparib plus bevacizumab (N = 249)	Placebo plus bevacizumab (N = 127)	Between-group difference	Treatment-by-time interaction
W72	−2.26 (−5.48 to 0.95)	−1.96 (−6.56 to 2.63)	−0.30 (−5.91 to 5.31)	
W84	−1.75 (−5.05 to 1.55)	−1.86 (−6.65 to 2.92)	0.11 (−5.70 to 5.93)	
W96	−1.83 (−5.17 to 1.51)	1.67 (−3.19 to 6.52)	−3.50 (−9.39 to 2.40)	
Insomnia[a]				
Baseline	N = 245	N = 127		
Mean (SD)	28.4 (30.1)	22.0 (24.9)		
Median (range)	33.3 (0-100)	33.3 (0-100)		
Adjusted mean global change from baseline (95% CI)	4.69 (2.21 to 7.18)	7.12 (3.59 to 10.66)	−2.43 (−6.76 to 1.90)	*P*=.7011
Adjusted mean change from baseline (95% CI)				
W12	3.93 (0.45 to 7.42)	7.99 (3.14 to 12.84)	−4.06 (−10.05 to 1.93)	
W24	6.01 (2.48 to 9.53)	4.48 (−0.56 to 9.52)	1.53 (−4.64 to 7.70)	
W36	5.54 (1.95 to 9.13)	6.33 (1.22 to 11.44)	−0.79 (−7.05 to 5.47)	
W48	5.15 (1.57 to 8.73)	6.65 (1.45 to 11.85)	−1.51 (−7.83 to 4.82)	
W60	6.72 (2.99 to 10.44)	9.73 (4.36 to 15.11)	−3.02 (−9.57 to 3.54)	
W72	1.90 (−1.84 to 5.64)	6.43 (1.11 to 11.75)	−4.54 (−11.05 to 1.98)	
W84	4.20 (0.38 to 8.01)	7.96 (2.38 to 13.54)	−3.76 (−10.54 to 3.01)	
W96	4.09 (0.21 to 7.98)	7.41 (1.74 to 13.08)	−3.32 (−10.20 to 3.57)	
Appetite loss[a]				
Baseline	N = 247	N = 127		
Mean (SD)	8.0 (17.1)	8.7 (17.4)		
Median (range)	0.0 (0-67)	0.0 (0-67)		
Adjusted mean global change from baseline (95% CI)	4.60 (2.78 to 6.41)	3.55 (0.95 to 6.14)	1.05 (−2.11 to 4.21)	*P*=.0003
Adjusted mean change from baseline (95% CI)				
W12	10.21 (7.50 to 12.91)	1.90 (−1.88 to 5.68)	8.31 (3.66 to 12.95)	
W24	5.44 (2.70 to 8.18)	2.18 (−1.75 to 6.11)	3.26 (−1.53 to 8.05)	
W36	4.93 (2.12 to 7.74)	1.97 (−2.04 to 5.98)	2.96 (−1.93 to 7.85)	
W48	7.59 (4.80 to 10.38)	4.09 (0.01 to 8.17)	3.50 (−1.44 to 8.44)	
W60	2.81 (−0.10 to 5.71)	5.01 (0.80 to 9.22)	−2.20 (−7.32 to 2.91)	

Scale scores	Olaparib plus bevacizumab (N = 249)	Placebo plus bevacizumab (N = 127)	Between-group difference	Treatment-by-time interaction
W72	2.51 (−0.42 to 5.44)	3.10 (−1.07 to 7.27)	−0.59 (−5.69 to 4.51)	
W84	0.09 (−2.91 to 3.09)	4.47 (0.06 to 8.88)	−4.38 (−9.72 to 0.96)	
W96	3.20 (0.15 to 6.25)	5.65 (1.20 to 10.11)	−2.45 (−7.85 to 2.95)	
Constipation[a]				
Baseline	N = 245	N = 125		
Mean (SD)	17.7 (27.2)	14.7 (24.5)		
Median (range)	0.0 (0-100)	0.0 (0-100)		
Adjusted mean global change from baseline (95% CI)	0.70 (−1.27 to 2.67)	2.44 (−0.38 to 5.26)	−1.74 (−5.18 to 1.71)	*P*=.5589
Adjusted mean change from baseline (95% CI)				
W12	−2.53 (−5.51 to 0.46)	−3.20 (−7.35 to 0.95)	0.68 (−4.44 to 5.79)	
W24	−2.04 (−5.04 to 0.96)	0.74 (−3.63 to 5.12)	−2.78 (−8.09 to 2.53)	
W36	−1.43 (−4.50 to 1.63)	0.66 (−3.71 to 5.03)	−2.09 (−7.43 to 3.26)	
W48	−1.59 (−4.66 to 1.48)	4.39 (−0.09 to 8.86)	−5.98 (−11.41 to −0.55)	
W60	2.66 (−0.53 to 5.86)	3.20 (−1.42 to 7.83)	−0.54 (−6.17 to 5.10)	
W72	2.11 (−1.11 to 5.34)	2.81 (−1.79 to 7.41)	−0.70 (−6.33 to 4.94)	
W84	3.40 (0.11 to 6.69)	5.77 (0.92 to 10.62)	−2.37 (−8.25 to 3.50)	
W96	5.03 (1.67 to 8.38)	5.13 (0.26 to 10.01)	−0.11 (−6.03 to 5.82)	
Diarrhea[a]				
Baseline	N = 245	N = 126		
Mean (SD)	9.5 (19.1)	13.0 (24.6)		
Median (range)	0.0 (0-100)	0.0 (0-100)		
Adjusted mean global change from baseline (95% CI)	0.14 (−1.48 to 1.75)	1.33 (−0.98 to 3.63)	−1.19 (−4.01 to 1.63)	*P*=.0409
Adjusted mean change from baseline (95% CI)				
W12	3.03 (0.59 to 5.47)	−1.33 (−4.75 to 2.08)	4.36 (0.16 to 8.57)	
W24	1.54 (−0.93 to 4.01)	0.44 (−3.12 to 4.00)	1.11 (−3.23 to 5.44)	
W36	−0.38 (−2.90 to 2.14)	1.47 (−2.14 to 5.09)	−1.85 (−6.27 to 2.56)	
W48	0.22 (−2.30 to 2.74)	1.48 (−2.20 to 5.16)	−1.26 (−5.73 to 3.20)	
W60	0.32 (−2.30 to 2.94)	3.55 (−0.25 to 7.36)	−3.24 (−7.86 to 1.39)	

Scale scores	Olaparib plus bevacizumab (N = 249)	Placebo plus bevacizumab (N = 127)	Between-group difference	Treatment-by-time interaction
W72	−0.48 (−3.13 to 2.16)	1.10 (−2.71 to 4.90)	−1.58 (−6.23 to 3.07)	
W84	−2.61 (−5.32 to 0.10)	1.35 (−2.66 to 5.36)	−3.96 (−8.81 to 0.88)	
W96	−0.53 (−3.29 to 2.23)	2.54 (−1.48 to 6.56)	−3.07 (−7.96 to 1.82)	
Financial difficulties[a]				
Baseline	N = 246	N = 123		
Mean (SD)	17.8 (29.6)	19.8 (30.4)		
Median (range)	0.0 (0-100)	0.0 (0-100)		
Adjusted mean global change from baseline (95% CI)	−2.44 (−4.56 to −0.31)	−1.67 (−4.72 to 1.38)	−0.77 (−4.49 to 2.95)	*P*=.5157
Adjusted mean change from baseline (95% CI)				
W12	−1.30 (−4.04 to 1.44)	0.71 (−3.16 to 4.59)	−2.01 (−6.75 to 2.74)	
W24	−1.78 (−4.55 to 1.00)	−2.18 (−6.16 to 1.80)	0.40 (−4.45 to 5.25)	
W36	−1.97 (−4.78 to 0.84)	−4.16 (−8.17 to −0.14)	2.18 (−2.72 to 7.09)	
W48	−1.71 (−4.51 to 1.10)	−2.47 (−6.55 to 1.62)	0.76 (−4.20 to 5.71)	
W60	−2.67 (−5.55 to 0.21)	−1.01 (−5.21 to 3.19)	−1.66 (−6.75 to 3.44)	
W72	−3.17 (−6.08 to −0.26)	−1.53 (−5.72 to 2.65)	−1.64 (−6.74 to 3.47)	
W84	−3.38 (−6.33 to −0.43)	−0.40 (−4.74 to 3.95)	−2.98 (−8.23 to 2.27)	
W96	−3.53 (−6.52 to −0.54)	−2.31 (−6.69 to 2.08)	−1.22 (−6.53 to 4.08)	

Mixed model for repeated measures analysis. When time-by-treatment interactions are significant, time-specific estimates may be more relevant than estimates of mean global change.

[a]Symptomatic scale where a high score represents a high symptomatic level. For all other HRQoL scales, a high score represents a high QoL level.

CI = confidence interval; EORTC QLQ-C30 = European Organisation for Research and Treatment of Cancer Core Quality of Life Questionnaire; HRD = homologous recombination deficiency; HRQoL = health-related quality of life; QoL = quality of life; SD = standard deviation; W = week.

Table S7. Baseline EORTC QLQ-OV28 scores and adjusted mean change from baseline in the HRD-positive subgroup

Scale scores	Olaparib plus bevacizumab (N = 249)	Placebo plus bevacizumab (N = 127)	Between-group difference	Treatment-by-time interaction
Functional scales				
Body image				
Baseline	N = 247	N = 125		
Mean (SD)	61.8 (31.7)	60.0 (33.9)		
Median (range)	66.7 (0-100)	66.7 (0-100)		
Adjusted mean global change from baseline (95% CI)	9.34 (6.96 to 11.72)	6.73 (3.33 to 10.13)	2.61 (−1.55 to 6.76)	*P*=.0361
Adjusted mean change from baseline (95% CI)				
W12	4.81 (1.73 to 7.89)	7.56 (3.22 to 11.90)	−2.75 (−8.07 to 2.57)	
W24	7.59 (4.48 to 10.71)	5.35 (0.89 to 9.81)	2.25 (−3.20 to 7.69)	
W36	7.89 (4.72 to 11.06)	7.16 (2.63 to 11.69)	0.73 (−4.80 to 6.26)	
W48	9.62 (6.47 to 12.77)	8.59 (4.00 to 13.18)	1.03 (−4.54 to 6.60)	
W60	10.84 (7.61 to 14.08)	7.20 (2.48 to 11.91)	3.65 (−2.07 to 9.36)	
W72	10.88 (7.62 to 14.14)	7.21 (2.53 to 11.89)	3.68 (−2.02 to 9.38)	
W84	12.36 (9.03 to 15.69)	7.73 (2.88 to 12.58)	4.63 (−1.26 to 10.51)	
W96	10.70 (7.31 to 14.08)	3.05 (−1.88 to 7.99)	7.64 (1.66 to 13.62)	
Sexual function				
Baseline	N = 235	N = 122		
Mean (SD)	15.1 (21.3)	17.8 (22.3)		
Median (range)	0.0 (0-83)	0.0 (0-83)		
Adjusted mean global change from baseline (95% CI)	2.20 (0.31 to 4.08)	3.12 (0.47 to 5.77)	−0.92 (−4.18 to 2.33)	*P*=.2350
Adjusted mean change from baseline (95% CI)				
W12	2.09 (−0.41 to 4.60)	4.71 (1.23 to 8.18)	−2.61 (−6.90 to 1.68)	
W24	2.98 (0.44 to 5.53)	4.89 (1.30 to 8.48)	−1.91 (−6.31 to 2.49)	
W36	1.74 (−0.89 to 4.37)	3.38 (−0.33 to 7.09)	−1.64 (−6.19 to 2.91)	
W48	0.47 (−2.15 to 3.08)	3.84 (0.10 to 7.59)	−3.38 (−7.95 to 1.20)	
W60	1.24 (−1.46 to 3.93)	3.09 (−0.75 to 6.93)	−1.85 (−6.55 to 2.84)	

Scale scores	Olaparib plus bevacizumab (N = 249)	Placebo plus bevacizumab (N = 127)	Between-group difference	Treatment-by-time interaction
W72	3.11 (0.38 to 5.83)	0.19 (−3.65 to 4.03)	2.92 (−1.79 to 7.63)	
W84	3.22 (0.43 to 6.01)	2.24 (−1.74 to 6.22)	0.98 (−3.88 to 5.84)	
W96	2.72 (−0.09 to 5.53)	2.62 (−1.37 to 6.61)	0.10 (−4.79 to 4.98)	
Attitude to disease/treatment				
Baseline	N = 245	N = 125		
Mean (SD)	43.2 (27.6)	42.7 (27.1)		
Median (range)	44.4 (0-100)	44.4 (0-100)		
Adjusted mean global change from baseline (95% CI)	11.98 (9.80 to 14.16)	10.26 (7.15 to 13.38)	1.72 (−2.08 to 5.52)	*P*<.0001
Adjusted mean change from baseline (95% CI)				
W12	4.69 (1.77 to 7.61)	8.99 (4.86 to 13.12)	−4.30 (−9.36 to 0.76)	
W24	7.16 (4.21 to 10.12)	9.39 (5.16 to 13.63)	−2.23 (−7.39 to 2.93)	
W36	9.53 (6.52 to 12.54)	10.10 (5.78 to 14.41)	−0.57 (−5.83 to 4.69)	
W48	11.31 (8.31 to 14.31)	13.03 (8.64 to 17.42)	−1.72 (−7.04 to 3.60)	
W60	14.29 (11.22 to 17.36)	11.20 (6.68 to 15.72)	3.09 (−2.37 to 8.55)	
W72	16.31 (13.19 to 19.42)	12.45 (7.99 to 16.90)	3.86 (−1.58 to 9.29)	
W84	15.97 (12.78 to 19.15)	9.70 (5.05 to 14.35)	6.26 (0.63 to 11.90)	
W96	16.58 (13.35 to 19.81)	7.24 (2.48 to 11.99)	9.34 (3.59 to 15.09)	
Symptomatic scales				
Abdominal/GI symptoms[a]				
Baseline	N = 249	N = 126		
Mean (SD)	19.4 (17.6)	19.5 (15.9)		
Median (range)	14.3 (0-81)	14.3 (0-67)		
Adjusted mean global change from baseline (95% CI)	2.36 (0.95 to 3.76)	3.82 (1.80 to 5.84)	−1.46 (−3.92 to 1.00)	*P*=.3475
Adjusted mean change from baseline (95% CI)				
W12	2.78 (0.87 to 4.68)	1.23 (−1.46 to 3.92)	1.55 (−1.75 to 4.85)	
W24	1.12 (−0.80 to 3.05)	3.45 (0.69 to 6.22)	−2.33 (−5.70 to 1.04)	
W36	2.21 (0.24 to 4.18)	3.74 (0.94 to 6.54)	−1.53 (−4.95 to 1.89)	
W48	2.80 (0.84 to 4.75)	4.25 (1.39 to 7.11)	−1.46 (−4.92 to 2.00)	

Scale scores	Olaparib plus bevacizumab (N = 249)	Placebo plus bevacizumab (N = 127)	Between-group difference	Treatment-by-time interaction
W60	2.48 (0.47 to 4.49)	5.21 (2.27 to 8.15)	−2.73 (−6.29 to 0.83)	
W72	1.34 (−0.69 to 3.37)	2.79 (−0.13 to 5.71)	−1.45 (−5.00 to 2.11)	
W84	1.89 (−0.18 to 3.97)	4.59 (1.55 to 7.63)	−2.69 (−6.37 to 0.98)	
W96	4.24 (2.12 to 6.35)	5.28 (2.19 to 8.37)	−1.04 (−4.80 to 2.71)	
Peripheral neuropathy[a]				
Baseline	N = 249	N = 125		
Mean (SD)	39.3 (28.9)	41.6 (29.2)		
Median (range)	33.3 (0-100)	33.3 (0-100)		
Adjusted mean global change from baseline (95% CI)	−11.26 (−13.29 to −9.23)	−11.51 (−14.43 to −8.60)	0.25 (−3.30 to 3.81)	*P*=.9302
Adjusted mean change from baseline (95% CI)				
W12	−4.41 (−7.04 to −1.77)	−5.33 (−9.08 to −1.58)	0.92 (−3.67 to 5.50)	
W24	−10.64 (−13.31 to −7.97)	−8.96 (−12.81 to −5.11)	−1.68 (−6.36 to 3.00)	
W36	−10.99 (−13.72 to −8.26)	−11.73 (−15.63 to −7.84)	0.75 (−4.01 to 5.50)	
W48	−11.54 (−14.25 to −8.83)	−13.08 (−17.06 to −9.10)	1.54 (−3.28 to 6.36)	
W60	−12.07 (−14.85 to −9.30)	−12.24 (−16.31 to −8.17)	0.17 (−4.76 to 5.10)	
W72	−14.52 (−17.32 to 11.72)	−14.05 (−18.10 to 10.01)	−0.47 (−5.39 to 4.45)	
W84	−13.57 (−16.42 to 10.71)	−13.33 (−17.53 to −9.13)	−0.24 (−5.31 to 4.84)	
W96	−12.34 (−15.26 to −9.42)	−13.39 (−17.64 to −9.14)	1.05 (−4.12 to 6.22)	
Hormonal/menopausal symptoms[a]				
Baseline	N = 248	N = 124		
Mean (SD)	26.1 (29.8)	26.2 (31.5)		
Median (range)	16.7 (0-100)	16.7 (0-100)		
Adjusted mean global change from baseline (95% CI)	−2.63 (−4.82 to −0.45)	−0.74 (−3.89 to 2.41)	−1.89 (−5.73 to 1.94)	*P*=.0282
Adjusted mean change from baseline (95% CI)				
W12	−4.33 (−7.18 to −1.47)	3.24 (−0.84 to 7.32)	−7.57 (−12.55 to −2.58)	
W24	−4.17 (−7.07 to −1.27)	−0.57 (−4.76 to 3.62)	−3.60 (−8.69 to 1.49)	
W36	−2.95 (−5.90 to 0.00)	−3.12 (−7.34 to 1.09)	0.17 (−4.97 to 5.32)	
W48	−4.12 (−7.06 to −1.19)	−3.10 (−7.41 to 1.21)	−1.02 (−6.23 to 4.20)	

Scale scores	Olaparib plus bevacizumab (N = 249)	Placebo plus bevacizumab (N = 127)	Between-group difference	Treatment-by-time interaction
W60	−3.98 (−6.99 to −0.97)	−0.49 (−4.91 to 3.92)	−3.49 (−8.83 to 1.86)	
W72	−1.13 (−4.16 to 1.91)	−0.48 (−4.86 to 3.91)	−0.65 (−5.98 to 4.69)	
W84	0.54 (−2.57 to 3.64)	−0.94 (−5.48 to 3.61)	1.47 (−4.04 to 6.98)	
W96	−0.93 (−4.08 to 2.22)	−0.46 (−5.08 to 4.17)	−0.48 (−6.07 to 5.12)	
Other chemotherapy side effects[a]				
Baseline	N = 249	N = 125		
Mean (SD)	23.3 (16.9)	22.4 (16.5)		
Median (range)	20.0 (0-73)	20.0 (0-87)		
Adjusted mean global change from baseline (95% CI)	−2.78 (−4.03 to −1.53)	−1.95 (−3.75 to −0.16)	−0.83 (−3.02 to 1.36)	*P*=.1098
Adjusted mean change from baseline (95% CI)				
W12	0.86 (−0.84 to 2.55)	−0.30 (−2.71 to 2.11)	1.16 (−1.79 to 4.10)	
W24	−0.32 (−2.04 to 1.40)	0.81 (−1.67 to 3.29)	−1.13 (−4.15 to 1.89)	
W36	−0.73 (−2.49 to 1.03)	−2.33 (−4.84 to 0.18)	1.61 (−1.46 to 4.67)	
W48	−2.51 (−4.26 to −0.76)	−1.57 (−4.14 to 1.00)	−0.95 (−4.06 to 2.17)	
W60	−4.30 (−6.09 to −2.50)	−2.34 (−4.97 to 0.30)	−1.96 (−5.15 to 1.23)	
W72	−4.65 (−6.46 to −2.84)	−3.90 (−6.52 to −1.28)	−0.75 (−3.94 to 2.44)	
W84	−6.19 (−8.05 to −4.33)	−2.83 (−5.56 to −0.10)	−3.36 (−6.66 to −0.06)	
W96	−4.41 (−6.30 to −2.52)	−3.17 (−5.94 to −0.41)	−1.23 (−4.58 to 2.11)	
Hair loss[a]				
Baseline	N = 241	N = 125		
Mean (SD)	27.9 (36.6)	34.4 (39.6)		
Median (range)	0.0 (0-100)	0.0 (0-100)		
Adjusted mean global change from baseline (95% CI)	−21.15 (−22.83 to 19.47)	−19.90 (−22.28 to 17.51)	−1.25 (−4.17 to 1.66)	*P*=.9463
Adjusted mean change from baseline (95% CI)				
W12	−24.08 (−26.80 to 21.36)	−22.75 (−26.55 to −18.94)	−1.34 (−6.02 to 3.34)	
W24	−26.43 (−29.21 to 23.66)	−22.82 (−26.77 to 18.87)	−3.61 (−8.44 to 1.23)	
W36	−25.45 (−28.31 to −22.60)	−24.85 (−28.83 to 20.87)	−0.60 (−5.50 to 4.30)	
W48	−24.47 (−27.29 to −21.65)	−24.93 (−29.02 to 20.84)	0.46 (−4.52 to 5.43)	

Scale scores	Olaparib plus bevacizumab (N = 249)	Placebo plus bevacizumab (N = 127)	Between-group difference	Treatment-by-time interaction
W60	−19.86 (−22.80 to 16.92)	−19.45 (−23.69 to 15.21)	−0.41 (−5.57 to 4.74)	
W72	−16.62 (−19.57 to −13.67)	−14.41 (−18.63 to −10.18)	−2.21 (−7.36 to 2.94)	
W84	−16.77 (−19.82 to 13.72)	−15.49 (−19.90 to 11.07)	−1.28 (−6.64 to 4.08)	
W96	−15.51 (−18.66 to 12.36)	−14.49 (−19.01 to −9.96)	−1.03 (−6.56 to 4.50)	

Mixed model for repeated measures analysis. When time-by-treatment interactions are significant, time-specific estimates may be more relevant than estimates of mean global change.

[a]Symptomatic scale where a high score represents a high symptomatic level. For all other HRQoL scales, a high score represents a high QoL level.

CI = confidence interval; EORTC QLQ-OV28 = Quality of Life Questionnaire Ovarian Cancer module; GI = gastrointestinal; HRD = homologous recombination deficiency; HRQoL = health-related quality of life; QoL = quality of life; SD = standard deviation; W = week.

Table S8. Baseline EORTC QLQ-C30 scores and mean change from baseline in the HRD-negative subgroup

Scale scores	Olaparib plus bevacizumab (N = 182)	Placebo plus bevacizumab (N = 78)	Between-group difference	Treatment-by-time interaction
Functional scales				
Global Health Status				
Baseline	N = 180	N = 77		
Mean (SD)	67.5 (18.2)	63.6 (17.6)		
Median (range)	66.7 (0-100)	66.7 (25-100)		
Adjusted mean global change from baseline (95% CI)	−2.24 (−4.29 to −0.18)	−3.53 (−6.57 to −0.49)	1.30 (−2.39 to 4.98)	*P*=.2146
Adjusted mean change from baseline (95% CI)				
W12	−3.44 (−6.25 to −0.63)	−022 (−4.47 to 4.03)	−3.22 (−8.34 to 1.90)	
W24	−2.11 (−5.00 to 0.77)	−1.68 (−6.12 to 2.76)	−0.43 (−5.74 to 4.88)	
W36	−3.50 (−6.58 to −0.43)	−6.42 (−10.91 to −1.93)	2.91 (−2.54 to 8.37)	
W48	−1.70 (−4.79 to 1.40)	−2.34 (−6.88 to 2.20)	0.65 (−4.86 to 6.15)	
W60	−0.29 (−3.63 to 3.05)	−5.22 (−9.98 to −0.47)	4.93 (−0.90 to 10.77)	
W72	−0.40 (−3.79 to 2.98)	−4.15 (−8.99 to 0.69)	3.74 (−2.18 to 9.67)	
W84	−4.72 (−8.29 to −1.16)	−4.13 (−9.09 to 0.84)	−0.60 (−6.74 to 5.54)	
W96	−1.71 (−5.44 to 2.01)	−4.08 (−9.38 to 1.21)	2.37 (−4.11 to 8.86)	
Physical functioning				
Baseline	N = 181	N = 78		
Mean (SD)	77.8 (18.8)	73.7 (20.7)		
Median (range)	80.0 (20-100)	76.7 (27-100)		
Adjusted mean global change from baseline (95% CI)	−1.21 (−3.15 to 0.72)	−0.69 (−3.54 to 2.15)	−0.52 (−3.97 to 2.93)	*P*=.5395
Adjusted mean change from baseline (95% CI)				
W12	−2.24 (−4.77 to 0.30)	0.16 (−3.69 to 4.00)	−2.39 (−7.01 to 2.22)	
W24	−0.88 (−3.48 to 1.72)	2.30 (−1.65 to 6.24)	−3.18 (−7.91 to 1.56)	
W36	−2.54 (−5.28 to 0.21)	−3.49 (−7.51 to 0.53)	0.96 (−3.93 to 5.84)	
W48	−0.68 (−3.44 to 2.09)	−1.26 (−5.30 to 2.78)	0.59 (−4.32 to 5.50)	
W60	−0.36 (−3.31 to 2.59)	−2.38 (−6.60 to 1.84)	2.02 (−3.15 to 7.18)	
W72	0.38 (−2.62 to 3.38)	0.53 (−3.73 to 4.80)	−0.16 (−5.40 to 5.09)	

Scale scores	Olaparib plus bevacizumab (N = 182)	Placebo plus bevacizumab (N = 78)	Between-group difference	Treatment-by-time interaction
W84	−3.14 (−6.28 to 0.01)	−1.22 (−5.62 to 3.17)	−1.91 (−7.34 to 3.52)	
W96	−0.26 (−3.53 to 3.02)	−0.18 (−4.80 to 4.43)	−0.08 (−5.75 to 5.60)	
Role functioning				
Baseline	N = 181	N = 78		
Mean (SD)	71.6 (28.4)	70.9 (29.0)		
Median (range)	66.7 (0-100)	66.7 (0-100)		
Adjusted mean global change from baseline (95% CI)	−1.66 (−4.42 to 1.10)	−3.82 (−7.87 to 0.23)	2.16 (−2.75 to 7.06)	*P*=.1024
Adjusted mean change from baseline (95% CI)				
W12	−1.34 (−5.05 to 2.37)	−4.05 (−9.63 to 1.53)	2.71 (−3.99 to 9.42)	
W24	−1.98 (−5.78 to 1.82)	0.15 (−5.64 to 5.93)	−2.13 (−9.05 to 4.79)	
W36	−3.18 (−7.23 to 0.87)	−6.60 (−12.50 to −0.70)	3.42 (−3.73 to 10.57)	
W48	−2.13 (−6.21 to 1.95)	−6.01 (−11.98 to −0.04)	3.88 (−3.36 to 11.12)	
W60	1.85 (−2.49 to 6.18)	−6.58 (−12.79 to −0.37)	8.42 (0.84 to 16.01)	
W72	0.53 (−3.88 to 4.95)	−4.58 (−10.84 to 1.69)	5.11 (−2.56 to 12.79)	
W84	−4.42 (−9.08 to 0.24)	−0.19 (−6.64 to 6.26)	−4.23 (−12.19 to 3.74)	
W96	−2.63 (−7.50 to 2.23)	−2.70 (−9.48 to 4.08)	0.07 (−8.28 to 8.42)	
Emotional functioning				
Baseline	N = 181	N = 78		
Mean (SD)	77.6 (21.2)	76.5 (20.0)		
Median (range)	83.3 (0-100)	83.3 (0-100)		
Adjusted mean global change from baseline (95% CI)	−3.52 (−5.89 to −1.16)	−6.26 (−9.76 to −2.76)	2.74 (−1.49 to 6.96)	*P*=.4044
Adjusted mean change from baseline (95% CI)				
W12	−3.17 (−6.17 to −0.16)	−2.17 (−6.69 to 2.36)	−1.00 (−6.43 to 4.43)	
W24	−0.79 (−3.87 to 2.28)	−3.49 (−8.17 to 1.19)	2.69 (−2.92 to 8.30)	
W36	−3.20 (−6.44 to 0.04)	−4.32 (−9.04 to 0.41)	1.12 (−4.61 to 6.85)	
W48	−3.23 (−6.50 to 0.05)	−5.07 (−9.86 to −0.27)	1.84 (−3.97 to 7.66)	
W60	−3.31 (−6.77 to 0.16)	−8.77 (−13.75 to −3.79)	5.46 (−0.60 to 11.52)	
W72	−3.20 (−6.71 to 0.31)	−8.99 (−14.04 to −3.95)	5.79 (−0.36 to 11.95)	

Scale scores	Olaparib plus bevacizumab (N = 182)	Placebo plus bevacizumab (N = 78)	Between-group difference	Treatment-by-time interaction
W84	−5.89 (−9.59 to −2.18)	−7.75 (−12.90 to −2.60)	1.86 (−4.48 to 8.21)	
W96	−5.42 (−9.26 to −1.58)	−9.53 (−14.98 to −4.08)	4.11 (−2.54 to 10.76)	
Cognitive functioning				
Baseline	N = 181	N = 78		
Mean (SD)	84.0 (20.6)	79.3 (22.8)		
Median (range)	83.3 (0-100)	83.3 (0-100)		
Adjusted mean global change from baseline (95% CI)	−1.55 (−3.80 to 0.70)	−5.20 (−8.54 to −1.86)	3.65 (−0.39 to 7.69)	*P*=.9107
Adjusted mean change from baseline (95% CI)				
W12	−0.19 (−3.04 to 2.67)	−3.10 (−7.41 to 1.21)	2.91 (−2.26 to 8.09)	
W24	−0.61 (−3.53 to 2.31)	−3.88 (−8.34 to 0.58)	3.27 (−2.08 to 8.62)	
W36	−2.26 (−5.34 to 0.81)	−3.78 (−8.28 to 0.72)	1.52 (−3.94 to 6.98)	
W48	0.45 (−2.68 to 3.57)	−5.13 (−9.70 to −0.55)	5.57 (0.01 to 11.14)	
W60	−0.94 (−4.25 to 2.37)	−4.70 (−9.45 to 0.06)	3.76 (−2.08 to 9.60)	
W72	−0.88 (−4.23 to 2.46)	−6.35 (−11.18 to −1.52)	5.47 (−0.44 to 11.38)	
W84	−5.14 (−8.68 to −1.60)	−8.40 (−13.33 to −3.47)	3.26 (−2.86 to 9.38)	
W96	−2.84 (−6.48 to 0.80)	−6.29 (−11.49 to −1.09)	3.45 (−2.92 to 9.82)	
Social functioning				
Baseline	N = 181	N = 78		
Mean (SD)	75.7 (25.2)	72.6 (26.5)		
Median (range)	83.3 (0-100)	75.0 (0-100)		
Adjusted mean global change from baseline (95% CI)	−0.04 (−2.69 to 2.60)	−2.22 (−6.11 to 1.67)	2.18 (−2.54 to 6.89)	*P*=.9509
Adjusted mean change from baseline (95% CI)				
W12	0.66 (−2.88 to 4.20)	−1.42 (−6.73 to 3.90)	2.08 (−4.33 to 8.48)	
W24	2.59 (−1.03 to 6.21)	2.22 (−3.30 to 7.73)	0.37 (−6.24 to 6.99)	
W36	−0.58 (−4.41 to 3.25)	−1.92 (−7.50 to 3.66)	1.34 (−5.44 to 8.12)	
W48	1.30 (−2.59 to 5.19)	−0.55 (−6.21 to 5.11)	1.85 (−5.04 to 8.74)	
W60	−0.79 (−4.92 to 3.35)	−6.29 (−12.21 to -0.37)	5.50 (−1.75 to 12.76)	
W72	1.86 (−2.34 to 6.07)	−1.64 (−7.66 to 4.38)	3.50 (−3.89 to 10.89)	

Scale scores	Olaparib plus bevacizumab (N = 182)	Placebo plus bevacizumab (N = 78)	Between-group difference	Treatment-by-time interaction
W84	−1.32 (−5.77 to 3.13)	−2.38 (−8.56 to 3.79)	1.06 (−6.30 to 9.74)	
W96	−4.07 (−8.68 to 0.54)	−5.79 (−12.34 to 0.76)	1.72 (−6.30 to 9.74)	
Symptomatic scales				
Fatigue[a]				
Baseline	N = 181	N = 78		
Mean (SD)	34.9 (22.9)	36.5 (23.8)		
Median (range)	33.3 (0-100)	33.3 (0-100)		
Adjusted mean global change from baseline (95% CI)	2.52 (0.14 to 4.90)	2.28 (−1.23 to 5.78)	0.24 (−4.00 to 4.49)	*P*=.2989
Adjusted mean change from baseline (95% CI)				
W12	4.12 (0.87 to 7.37)	−1.20 (−6.09 to 3.69)	5.32 (−0.56 to 11.20)	
W24	2.98 (−0.35 to 6.31)	0.46 (−4.61 to 5.54)	5.25 (−3.56 to 8.59)	
W36	5.90 (2.35 to 9.44)	4.27 (−0.89 to 9.44)	1.62 (−4.65 to 7.90)	
W48	2.72 (−0.86 to 6.29)	2.74 (−2.47 to 7.94)	−0.02 (−6.34 to 6.30)	
W60	0.31 (−3.50 to 4.12)	3.55 (−1.91 to 9.02)	−3.24 (−9.92 to 3.43)	
W72	−1.01 (−4.89 to 2.88)	2.01 (−3.50 to 7.51)	−3.01 (−9.77 to 3.74)	
W84	3.13 (−0.98 to 7.23)	2.72 (−2.96 to 8.40)	0.41 (−6.63 to 7.44)	
W96	2.02 (−2.27 to 6.31)	3.66 (−2.35 to 9.66)	−1.64 (−9.05 to 5.78)	
Nausea and vomiting[a]				
Baseline	N = 181	N = 78		
Mean (SD)	5.3 (14.7)	5.1 (10.5)		
Median (range)	0.0 (0-100)	0.0 (0-33)		
Adjusted mean global change from baseline (95% CI)	6.13 (4.30 to 7.95)	2.99 (0.32 to 5.65)	3.14 (-0.09 to 6.38)	*P*=.4203
Adjusted mean change from baseline (95% CI)				
W12	8.96 (6.24 to 11.68)	1.81 (−2.30 to 5.91)	7.15 (2.23 to 12.08)	
W24	7.22 (4.42 to 10.02)	3.43 (−0.85 to 7.70)	3.79 (−1.32 to 8.90)	
W36	8.22 (5.20 to 11.23)	4.05 (−0.31 to 8.41)	4.17 (−1.14 to 9.47)	
W48	6.31 (3.28 to 9.35)	4.94 (0.54 to 9.34)	1.38 (−3.97 to 6.72)	
W60	2.69 (−0.56 to 5.95)	3.77 (−0.87 to 8.42)	−1.08 (−6.75 to 4.59)	

Scale scores	Olaparib plus bevacizumab (N = 182)	Placebo plus bevacizumab (N = 78)	Between-group difference	Treatment-by-time interaction
W72	4.69 (1.38 to 8.01)	1.32 (−3.35 to 6.00)	3.37 (−2.36 to 9.10)	
W84	5.10 (1.57 to 8.64)	1.40 (−3.44 to 6.24)	3.71 (−2.28 to 9.70)	
W96	5.81 (2.11 to 9.52)	3.16 (−1.97 to 8.30)	2.65 (−3.68 to 8.98)	
Pain[a]				
Baseline	N = 181	N = 78		
Mean (SD)	22.6 (26.2)	26.1 (22.4)		
Median (range)	16.7 (0-100)	16.7 (0-83)		
Adjusted mean global change from baseline (95% CI)	2.40 (−0.34 to 5.13)	7.51 (3.50 to 11.53)	−5.11 (−9.98 to −0.25)	*P*=.9358
Adjusted mean change from baseline (95% CI)				
W12	1.80 (−1.95 to 5.56)	6.52 (0.85 to 12.19)	−4.72 (−11.54 to 2.10)	
W24	2.06 (−1.80 to 5.93)	7.92 (2.00 to 13.85)	−5.86 (−12.94 to 1.22)	
W36	6.26 (2.15 to 10.37)	12.69 (6.74 to 18.64)	−6.43 (−13.67 to 0.80)	
W48	8.28 (4.14 to 12.42)	9.46 (3.42 to 15.51)	−1.18 (−8.53 to 6.16)	
W60	0.12 (−4.31 to 4.55)	7.10 (0.77 to 13.43)	−6.98 (−14.72 to 0.76)	
W72	0.44 (−4.07 to 4.95)	5.95 (−0.44 to 12.34)	−5.51 (−13.35 to 2.33)	
W84	0.84 (−3.92 to 5.61)	5.49 (−1.11 to 12.09)	−4.65 (−12.79 to 3.50)	
W96	−0.63 (−5.61 to 4.36)	4.95 (−2.01 to 11.92)	−5.58 (−14.13 to 2.97)	
Dyspnea[a]				
Baseline	N = 179	N = 78		
Mean (SD)	23.5 (24.9)	22.6 (29.7)		
Median (range)	33.3 (0-100)	33.3 (0-100)		
Adjusted mean global change from baseline (95% CI)	2.00 (−0.63 to 4.63)	0.62 (−3.20 to 4.45)	1.38 (−3.26 to 6.02)	*P*=.2085
Adjusted mean change from baseline (95% CI)				
W12	3.01 (−0.77 to 6.79)	−6.35 (−12.01 to -0.69)	9.36 (2.56 to 16.16)	
W24	1.60 (−2.29 to 5.49)	−1.37 (−7.29 to 4.55)	2.97 (−4.11 to 10.05)	
W36	2.00 (−2.25 to 6.25)	0.75 (−5.25 to 6.74)	1.25 (−6.10 to 8.60)	
W48	−0.35 (−4.54 to 3.84)	1.72 (−4.41 to 7.85)	−2.07 (−9.49 to 5.36)	
W60	1.09 (−3.39 to 5.57)	2.11 (−4.25 to 8.47)	−1.02 (−8.80 to 6.76)	

Scale scores	Olaparib plus bevacizumab (N = 182)	Placebo plus bevacizumab (N = 78)	Between-group difference	Treatment-by-time interaction
W72	2.36 (−2.22 to 6.94)	1.30 (−5.11 to 7.71)	1.06 (−6.82 to 8.94)	
W84	2.87 (−2.00 to 7.74)	3.76 (−2.87 to 10.40)	−0.89 (−9.11 to 7.32)	
W96	3.43 (−1.69 to 8.55)	3.06 (−4.02 to 10.14)	0.37 (−8.36 to 9.09)	
Insomnia[a]				
Baseline	N = 181	N = 78		
Mean (SD)	26.0 (30.9)	25.6 (29.4)		
Median (range)	33.3 (0-100)	33.3 (0-100)		
Adjusted mean global change from baseline (95% CI)	1.96 (−0.78 to 4.69)	3.91 (−0.12 to 7.93)	−1.95 (−6.82 to 2.92)	*P*=.9083
Adjusted mean change from baseline (95% CI)				
W12	−1.41 (−5.12 to 2.31)	1.93 (−3.68 to 7.54)	−3.34 (−10.07 to 3.39)	
W24	1.24 (−2.58 to 5.05)	1.10 (−4.76 to 6.95)	0.14 (−6.85 to 7.13)	
W36	3.95 (−0.12 to 8.02)	4.20 (−1.72 to 10.12)	−0.25 (−7.44 to 6.94)	
W48	1.69 (−2.42 to 5.79)	1.30 (−4.67 to 7.27)	0.39 (−6.85 to 7.64)	
W60	3.50 (−0.88 to 7.87)	5.00 (−1.28 to 11.28)	−1.51 (−9.19 to 6.17)	
W72	0.32 (−4.12 to 4.76)	5.02 (−1.28 to 11.32)	−4.70 (−12.41 to 3.02)	
W84	3.66 (−1.06 to 8.37)	6.95 (0.44 to 13.47)	−3.30 (−11.36 to 4.77)	
W96	2.72 (−2.20 to 7.63)	5.75 (−1.13 to 12.62)	−3.03 (−11.50 to 5.45)	
Appetite loss[a]				
Baseline	N = 180	N = 78		
Mean (SD)	10.0 (20.8)	12.4 (25.8)		
Median (range)	0.0 (0-100)	0.0 (0-100)		
Adjusted mean global change from baseline (95% CI)	5.36 (2.69 to 8.02)	4.21 (0.32 to 8.11)	1.14 (−3.58 to 5.87)	*P*=.0160
Adjusted mean change from baseline (95% CI)				
W12	6.36 (2.59 to 10.13)	−1.71 (−7.37 to 3.95)	8.07 (1.27 to 14.88)	
W24	5.05 (1.16 to 8.94)	−1.77 (−7.67 to 4.13)	6.82 (−0.24 to 13.89)	
W36	9.38 (5.24 to 13.53)	4.17 (−1.87 to 10.22)	5.21 (−2.14 to 12.55)	
W48	8.01 (3.83 to 12.20)	6.66 (0.61 to 12.72)	1.35 (−6.01 to 8.70)	
W60	3.47 (−1.02 to 7.96)	7.12 (0.78 to 13.47)	−3.65 (−11.46 to 4.15)	

Scale scores	Olaparib plus bevacizumab (N = 182)	Placebo plus bevacizumab (N = 78)	Between-group difference	Treatment-by-time interaction
W72	4.28 (−0.28 to 8.84)	3.06 (−3.33 to 9.46)	1.21 (−6.63 to 9.06)	
W84	3.72 (−1.10 to 8.54)	7.80 (1.20 to 14.40)	−4.08 (−12.25 to 4.10)	
W96	2.58 (−2.49 to 7.66)	8.37 (1.40 to 15.33)	−5.79 (−14.41 to 2.83)	
Constipation[a]				
Baseline	N = 181	N = 78		
Mean (SD)	17.1 (26.9)	20.9 (31.4)		
Median (range)	0.0 (0-100)	0.0 (0-100)		
Adjusted mean global change from baseline (95% CI)	0.45 (−2.04 to 2.94)	0.22 (−3.43 to 3.87)	0.23 (−4.19 to 4.65)	*P*=.2213
Adjusted mean change from baseline (95% CI)				
W12	−2.24 (−5.81 to 1.33)	−3.57 (−8.97 to 1.84)	1.33 (−5.15 to 7.81)	
W24	−2.03 (−5.74 to 1.68)	−2.94 (−8.62 to 2.74)	0.91 (−5.89 to 7.71)	
W36	2.68 (−1.30 to 6.66)	0.48 (−5.24 to 6.20)	2.20 (−4.77 to 9.17)	
W48	0.74 (−3.24 to 4.73)	−2.64 (−8.41 to 3.13)	3.38 (−3.63 to 10.39)	
W60	0.32 (−3.95 to 4.58)	2.33 (−3.74 to 8.40)	−2.01 (−9.44 to 5.41)	
W72	−0.60 (−4.92 to 3.72)	5.25 (−0.92 to 11.41)	−5.85 (−13.38 to 1.68)	
W84	1.64 (−2.98 to 6.26)	5.58 (−0.74 to 11.91)	−3.95 (−11.79 to 3.90)	
W96	3.08 (−1.74 to 7.89)	−2.74 (−9.49 to 4.01)	5.82 (−2.47 to 14.11)	
Diarrhea[a]				
Baseline	N = 179	N = 78		
Mean (SD)	11.9 (23.3)	15.4 (23.8)		
Median (range)	0.0 (0-100)	0.0 (0-100)		
Adjusted mean global change from baseline (95% CI)	−2.14 (−4.34 to 0.07)	1.92 (−1.28 to 5.11)	−4.05 (−7.94 to −0.17)	*P*=.2954
Adjusted mean change from baseline (95% CI)				
W12	1.41 (−1.98 to 4.79)	1.86 (−3.23 to 6.95)	−0.45 (−6.58 to 5.67)	
W24	−1.26 (−4.75 to 2.23)	4.67 (−0.67 to 10.01)	−5.92 (−12.30 to 0.46)	
W36	0.26 (−3.51 to 4.03)	6.99 (1.55 to 12.43)	−6.73 (−13.37 to −0.10)	
W48	−1.84 (−5.64 to 1.96)	7.26 (1.78 to 12.73)	−9.10 (−15.76 to −2.43)	
W60	−5.51 (−9.62 to −1.40)	0.14 (−5.65 to 5.93)	−5.65 (−12.78 to 1.47)	

Scale scores	Olaparib plus bevacizumab (N = 182)	Placebo plus bevacizumab (N = 78)	Between-group difference	Treatment-by-time interaction
W72	−5.00 (−9.20 to −0.81)	−1.08 (−6.91 to 4.76)	−3.93 (−11.13 to 3.28)	
W84	−4.59 (−9.12 to −0.05)	−5.31 (−11.41 to 0.78)	0.73 (−6.87 to 8.33)	
W96	−0.55 (−5.24 to 4.14)	0.81 (−5.69 to 7.30)	−1.36 (−9.37 to 6.66)	
Financial difficulties[a]				
Baseline	N = 179	N = 77		
Mean (SD)	10.2 (24.0)	14.7 (27.8)		
Median (range)	0.0 (0-100)	0.0 (0-100)		
Adjusted mean global change from baseline (95% CI)	0.70 (−1.40 to 2.79)	1.31 (−1.79 to 4.40)	−0.61 (−4.35 to 3.13)	*P*=.1739
Adjusted mean change from baseline (95% CI)				
W12	−0.31 (−3.09 to 2.47)	−2.15 (−6.32 to 2.02)	1.84 (−3.18 to 6.85)	
W24	−1.30 (−4.14 to 1.54)	0.61 (−3.72 to 4.94)	−1.91 (−7.10 to 3.28)	
W36	−0.37 (−3.35 to 2.62)	4.38 (0.00 to 8.75)	−4.74 (−10.04 to 0.56)	
W48	−0.35 (−3.39 to 2.70)	1.90 (−2.53 to 6.33)	−2.25 (−7.65 to 3.15)	
W60	0.72 (−2.51 to 3.96)	0.26 (−4.40 to 4.91)	0.47 (−5.22 to 6.15)	
W72	1.38 (−1.90 to 4.65)	4.17 (−0.50 to 8.84)	−2.79 (−8.51 to 2.93)	
W84	3.93 (0.42 to 7.44)	0.63 (−4.23 to 5.50)	3.30 (−2.77 to 9.36)	
W96	1.86 (−1.77 to 5.48)	0.64 (−4.55 to 5.82)	1.22 (−5.16 to 7.60)	

Mixed model for repeated measures analysis. When time-by-treatment interactions are significant, time-specific estimates may be more relevant than estimates of mean global change.

[a]Symptomatic scale where a high score represents a high symptomatic level. For all other HRQoL scales, a high score represents a high QoL level.

CI = confidence interval; EORTC QLQ-C30 = European Organisation for Research and Treatment of Cancer Core Quality of Life Questionnaire; HRD = homologous recombination deficiency; HRQoL = health-related quality of life; QoL = quality of life; SD = standard deviation; W = week.

Table S9. Baseline EORTC QLQ-OV28 scores and adjusted mean change from baseline in the HRD-negative subgroup

Scale scores	Olaparib plus bevacizumab (N = 182)	Placebo plus bevacizumab (N = 78)	Between-group difference	Treatment-by-time interaction
Functional scales				
Body image				
Baseline	N = 181	N = 75		
Mean (SD)	68.0 (30.3)	63.6 (29.5)		
Median (range)	66.7 (0-100)	66.7 (0-100)		
Adjusted mean global change from baseline (95% CI)	5.55 (2.67 to 8.43)	2.74 (−1.60 to 7.08)	2.81 (−2.40 to 8.02)	*P*=.3701
Adjusted mean change from baseline (95% CI)				
W12	5.09 (1.38 to 8.80)	4.75 (−0.98 to 10.48)	0.34 (−6.48 to 7.17)	
W24	7.17 (3.37 to 10.96)	6.57 (0.70 to 12.45)	0.59 (−6.40 to 7.59)	
W36	4.00 (0.00 to 8.01)	4.02 (−2.01 to 10.05)	−0.01 (−7.25 to 7.23)	
W48	4.18 (0.10 to 8.26)	3.51 (−2.46 to 9.49)	0.67 (−6.57 to 7.91)	
W60	7.46 (3.14 to 11.78)	0.37 (−5.86 to 6.59)	7.09 (−0.49 to 14.68)	
W72	4.70 (0.31 to 9.08)	1.99 (−4.33 to 8.31)	2.70 (−5.01 to 10.41)	
W84	6.62 (2.05 to 11.19)	3.91 (−2.59 to 10.41)	2.71 (−5.24 to 10.65)	
W96	5.19 (0.43 to 9.94)	−3.18 (−10.00 to 3.64)	8.36 (0.05 to 16.68)	
Sexual function				
Baseline	N = 174	N = 73		
Mean (SD)	12.6 (21.4)	8.1 (17.7)		
Median (range)	0.0 (0-100)	0.0 (0-75)		
Adjusted mean global change from baseline (95% CI)	0.46 (−1.65 to 2.57)	4.09 (0.98 to 7.20)	−3.64 (−7.40 to 0.13)	*P*=.2418
Adjusted mean change from baseline (95% CI)				
W12	1.19 (−1.50 to 3.87)	2.23 (−1.90 to 6.36)	−1.05 (−5.98 to 3.89)	
W24	1.65 (−1.19 to 4.49)	6.43 (2.12 to 10.75)	-4.78 (−9.96 to 0.40)	
W36	0.51 (−2.47 to 3.49)	8.43 (4.02 to 12.83)	−7.92 (−13.26 to −2.57)	
W48	−1.08 (−4.17 to 2.00)	3.63 (−0.79 to 8.05)	−4.72 (−10.13 to 0.69)	
W60	0.33 (−2.93 to 3.60)	1.41 (−3.13 to 5.95)	−1.08 (−6.68 to 4.52)	
W72	0.58 (−2.70 to 3.86)	3.34 (−1.32 to 8.00)	−2.76 (−8.46 to 2.94)	

Scale scores	Olaparib plus bevacizumab (N = 182)	Placebo plus bevacizumab (N = 78)	Between-group difference	Treatment-by-time interaction
W84	1.16 (−2.31 to 4.62)	3.42 (−1.35 to 8.19)	−2.26 (−8.17 to 3.65)	
W96	−0.69 (−4.27 to 2.90)	3.85 (−1.24 to 8.93)	−4.53 (−10.77 to 1.70)	
Attitude to disease/treatment				
Baseline	N = 181	N = 76		
Mean (SD)	45.7 (26.9)	42.4 (28.8)		
Median (range)	44.4 (0-100)	33.3 (0-100)		
Adjusted mean global change from baseline (95% CI)	7.20 (4.36 to 10.03)	5.62 (1.38 to 9.85)	1.58 (−3.52 to 6.68)	*P*=.2892
Adjusted mean change from baseline (95% CI)				
W12	4.55 (0.85 to 8.25)	8.76 (3.08 to 14.43)	−4.21 (−10.99 to 2.57)	
W24	6.25 (2.46 to 10.05)	6.15 (0.30 to 12.01)	0.10 (−6.88 to 7.08)	
W36	5.55 (1.54 to 9.56)	2.95 (−3.03 to 8.93)	2.60 (−4.60 to 9.81)	
W48	7.46 (3.38 to 11.55)	4.05 (−1.92 to 10.02)	3.41 (−3.83 to 10.66)	
W60	10.30 (5.97 to 14.63)	4.18 (−2.04 to 10.40)	6.12 (−1.46 to 13.71)	
W72	7.53 (3.14 to 11.92)	3.24 (−3.09 to 9.58)	4.29 (−3.44 to 12.01)	
W84	8.47 (3.87 to 13.06)	9.41 (2.90 to 15.92)	−0.94 (−8.91 to 7.02)	
W96	7.46 (2.67 to 12.24)	6.18 (−0.66 to 13.02)	1.28 (−7.07 to 9.63)	
Symptomatic scales				
Abdominal/GI symptoms[a]				
Baseline	N = 182	N = 76		
Mean (SD)	20.1 (17.9)	23.0 (18.8)		
Median (range)	14.3 (0-90)	19.0 (0-86)		
Adjusted mean global change from baseline (95% CI)	3.81 (1.93 to 5.69)	4.67 (1.87 to 7.48)	−0.86 (−4.25 to 2.52)	*P*=.1997
Adjusted mean change from baseline (95% CI)				
W12	0.21 (−2.35 to 2.78)	−0.88 (−4.84 to 3.09)	1.09 (−3.63 to 5.81)	
W24	2.59 (−0.06 to 5.23)	0.97 (−3.10 to 5.05)	1.62 (−3.25 to 6.49)	
W36	6.57 (3.76 to 9.38)	3.77 (−0.38 to 7.92)	2.79 (−2.22 to 7.81)	
W48	4.30 (1.45 to 7.14)	4.61 (0.46 to 8.77)	−0.32 (−5.35 to 4.72)	
W60	3.07 (0.00 to 6.14)	3.58 (−0.77 to 7.93)	−0.51 (−5.84 to 4.82)	

Scale scores	Olaparib plus bevacizumab (N = 182)	Placebo plus bevacizumab (N = 78)	Between-group difference	Treatment-by-time interaction
W72	3.59 (0.51 to 6.66)	7.29 (2.84 to 11.73)	−3.70 (−9.11 to 1.71)	
W84	3.89 (0.65 to 7.14)	7.65 (3.10 to 12.21)	−3.76 (−9.35 to 1.83)	
W96	6.27 (2.86 to 9.67)	10.39 (5.64 to 15.15)	−4.12 (−9.97 to 1.72)	
Peripheral neuropathy[a]				
Baseline	N = 182	N = 76		
Mean (SD)	46.6 (32.1)	48.5 (30.9)		
Median (range)	44.4 (0-100)	44.4 (0-100)		
Adjusted mean global change from baseline (95% CI)	−16.35 (−18.97 to −13.72)	−14.25 (−18.17 to −10.33)	−2.10 (−6.82 to 2.62)	*P*=.7070
Adjusted mean change from baseline (95% CI)				
W12	−12.21 (−15.51 to −8.90)	−10.53 (−15.61 to −5.44)	−1.68 (−7.74 to 4.38)	
W24	−13.87 (−17.28 to −10.45)	−9.22 (−14.42 to −4.02)	−4.65 (−10.87 to 1.57)	
W36	−12.01 (−15.60 to −8.42)	−13.44 (−18.76 to −8.13)	1.43 (−4.98 to 7.85)	
W48	−16.04 (−19.64 to −12.45)	−15.33 (−20.62 to −10.03)	−0.72 (−7.12 to 5.68)	
W60	−16.96 (−20.82 to −13.09)	−15.73 (−21.23 to −10.22)	−1.23 (−7.96 to 5.50)	
W72	−18.27 (−22.13 to −14.41)	−15.29 (−20.87 to −9.71)	−2.98 (−9.77 to 3.80)	
W84	−19.84 (−23.89 to −15.80)	−15.68 (−21.41 to −9.95)	−4.16 (−11.17 to 2.85)	
W96	−21.59 (−25.83 to −17.35)	−18.79 (−24.74 to −12.84)	−2.80 (−10.10 to 4.50)	
Hormonal/menopausal symptoms[a]				
Baseline	N = 182	N = 76		
Mean (SD)	19.9 (27.3)	19.5 (24.4)		
Median (range)	8.3 (0-100)	16.7 (0-100)		
Adjusted mean global change from baseline (95% CI)	−2.13 (−4.46 to 0.19)	1.03 (−2.43 to 4.50)	−3.17 (−7.34 to 1.01)	*P*=.3088
Adjusted mean change from baseline (95% CI)				
W12	−4.99 (−8.15 to −1.82)	−2.30 (−7.18 to 2.58)	−2.69 (−8.51 to 3.14)	
W24	−4.97 (−8.22 to −1.71)	1.40 (−3.61 to 6.41)	−6.37 (−12.34 to −0.40)	
W36	−3.55 (−7.03 to −0.08)	−3.29 (−8.39 to 1.81)	−0.26 (−6.43 to 5.91)	
W48	−3.03 (−6.53 to 0.47)	−0.24 (−5.36 to 4.87)	−2.79 (−8.99 to 3.40)	
W60	−0.24 (−3.98 to 3.50)	−2.36 (−7.73 to 3.00)	2.12 (−4.42 to 8.66)	

Scale scores	Olaparib plus bevacizumab (N = 182)	Placebo plus bevacizumab (N = 78)	Between-group difference	Treatment-by-time interaction
W72	0.52 (−3.26 to 4.31)	5.29 (−0.15 to 10.72)	−4.76 (−11.38 to 1.86)	
W84	0.27 (−3.73 to 4.28)	5.76 (0.14 to 11.37)	−5.48 (−12.39 to 1.43)	
W96	−1.09 (−5.28 to 3.11)	4.01 (−1.90 to 9.92)	−5.10 (−12.34 to 2.14)	
Other chemotherapy side effects[a]				
Baseline	N = 182	N = 76		
Mean (SD)	24.5 (17.6)	25.7 (19.0)		
Median (range)	20.0 (0-73)	2.00 (0-93)		
Adjusted mean global change from baseline (95% CI)	−4.44 (−6.07 to −2.80)	−3.16 (−5.60 to −0.71)	−1.28 (−4.22 to 1.66)	*P*=.6248
Adjusted mean change from baseline (95% CI)				
W12	−2.72 (−4.87 to −0.58)	−2.69 (−6.00 to 0.62)	−0.03 (−3.97 to 3.91)	
W24	−3.02 (−5.22 to −0.81)	−3.53 (−6.92 to −0.14)	0.51 (−3.53 to 4.56)	
W36	−1.63 (−3.98 to 0.72)	−1.01 (−-4.48 to 2.46)	−0.62 (−4.81 to 3.56)	
W48	−2.38 (−4.73 to −0.03)	−1.36 (−4.81 to 2.10)	−1.02 (−5.20 to 3.16)	
W60	−7.14 (−9.68 to −4.59)	−3.23 (−6.84 to 0.37)	−3.90 (−8.32 to 0.51)	
W72	−6.13 (−8.67 to −3.58)	−3.38 (−7.04 to 0.28)	−2.74 (−7.20 to 1.71)	
W84	−7.27 (−9.94 to −4.60)	−5.10 (−8.87 to −1.33)	−2.17 (−6.79 to 2.45)	
W96	−5.23 (−8.03 to −2.42)	−4.95 (−8.87 to −1.02)	−0.28 (−5.10 to 4.55)	
Hair loss[a]				
Baseline	N = 175	N = 75		
Mean (SD)	36.9 (38.3)	26.7 (37.6)		
Median (range)	33.3 (0-100)	0.0 (0-100)		
Adjusted mean global change from baseline (95% CI)	−22.72 (−24.96 to −20.48)	−23.62 (−26.89 to −20.35)	0.90 (−3.08 to 4.89)	*P*=.4233
Adjusted mean change from baseline (95% CI)				
W12	−24.96 (−28.47 to −21.44)	−24.54 (−29.84 to −19.23)	−0.42 (−6.81 to 5.97)	
W24	−29.27 (−32.90 to −25.63)	−29.18 (−34.69 to −23.67)	−0.08 (−6.71 to 6.54)	
W36	−29.07 (−32.94 to −25.20)	−25.31 (−30.94 to −19.68)	−3.76 (−10.63 to 3.11)	
W48	−23.68 (−27.61 to −19.74)	−25.93 (−31.62 to −20.23)	2.25 (−4.75 to 9.25)	
W60	−21.00 (−25.33 to −16.68)	−17.29 (−23.39 to −11.18)	−3.72 (−11.26 to 3.82)	

Scale scores	Olaparib plus bevacizumab (N = 182)	Placebo plus bevacizumab (N = 78)	Between-group difference	Treatment-by-time interaction
W72	−17.73 (−22.02 to −13.44)	−21.23 (−27.32 to −15.15)	3.50 (−3.96 to 10.96)	
W84	−17.03 (−21.60 to −12.45)	−20.91 (−27.26 to −14.57)	3.89 (−3.95 to 11.73)	
W96	−19.03 (−23.86 to −14.19)	−24.60 (−31.23 to −17.96)	5.57 (−2.69 to 13.82)	

Mixed model for repeated measures analysis. When time-by-treatment interactions are significant, time-specific estimates may be more relevant than estimates of mean global change.

[a]Symptomatic scale where a high score represents a high symptomatic level. For all other HRQoL scales, a high score represents a high QoL level.

CI = confidence interval; EORTC QLQ-OV28 = Quality of Life Questionnaire Ovarian Cancer module; GI = gastrointestinal; HRD = homologous recombination deficiency; HRQoL = health-related quality of life; QoL = quality of life; SD = standard deviation; W = Week.

Table S10. Mean change in EORTC QLQ-C30 scores post-progression by treatment arm

EORTC QLQ-C30 score	Olaparib plus bevacizumab	Placebo plus bevacizumab
**Functional scales**		
Global Health Status	N = 56	N = 46
Mean change (95% CI)	−9.38 (−15.89 to −2.86)	−5.07 (−11.88 to 1.74)
Physical functioning	N = 57	N = 46
Mean change (95% CI)	−3.16 (−7.39 to 1.07)	−2.21 (−5.79 to 1.37)
Role functioning	N = 57	N = 46
Mean change (95% CI)	−14.04 (−21.17 to −6.90)	−1.45 (−8.36 to 5.46)
Emotional functioning	N = 57	N = 46
Mean change (95% CI)	−12.09 (−17.60 to −6.57)	−12.56 (−19.15 to −5.97)
Cognitive functioning	N = 57	N = 46
Mean change (95% CI)	−3.80 (−8.22 to 0.62)	−8.70 (−13.99 to −3.40)
Social functioning	N = 57	N = 46
Mean change (95% CI)	−14.04 (−20.87 to −7.21)	−7.61 (−15.26 to 0.04)
**Symptomatic scales**		
Fatigue[a]	N = 57	N = 46
Mean change (95% CI)	7.89 (2.23 to 13.56)	3.50 (−3.41 to 10.42)
Nausea and vomiting[a]	N = 57	N = 46
Mean change (95% CI)	5.56 (−0.13 to 11.24)	0.72 (−5.36 to 6.80)
Pain[a]	N = 57	N = 46
Mean change (95% CI)	3.22 (−3.44 to 9.87)	2.17 (−5.61 to 9.95)
Dyspnea[a]	N = 57	N = 45
Mean change (95% CI)	4.68 (−2.69 to 12.05)	5.19 (−2.20 to 12.57)
Insomnia[a]	N = 57	N = 46
Mean change (95% CI)	9.36 (2.52 to 16.20)	1.45 (−7.64 to 10.54)
Appetite loss[a]	N = 57	N = 45
Mean change (95% CI)	7.60 (−0.92 to 16.13)	9.63 (1.19 to 18.07)
Constipation	N = 56	N = 45
Mean change (95% CI)	8.93 (0.68 to 17.18)	10.37 (2.73 to 18.02)
Diarrhea[a]	N = 57	N = 45
Mean change (95% CI)	−0.58 (−7.37 to 6.20)	4.44 (−2.17 to 11.06)
Financial difficulties[a]	N = 53	N = 45
Mean change (95% CI)	−0.00 (−5.70 to 5.70)	0.74 (−5.10 to 6.58)

EORTC QLQ-C30 scores following diagnosis of disease progression were compared with the last measurement collected prior to progression. Assessments were conducted up to a maximum of 60 days before and 60 days after progression.

[a]Symptomatic scale where higher scores represent higher symptomatic burden. For all other HRQoL scales, higher scores represent higher QoL level.

CI = confidence interval; EORTC QLQ-C30 = European Organisation for Research and Treatment of Cancer Core Quality of Life Questionnaire; HRQoL = health-related quality of life; QoL = quality of life.

Figure S2. Adjusted mean change over time in EORTC QLQ-C30 scores for (**A**) physical functioning, (**B**) role functioning, (**C**) emotional functioning, (**D**) cognitive functioning, and (**E**) social functioning in the modified ITT population

A Physical functioning

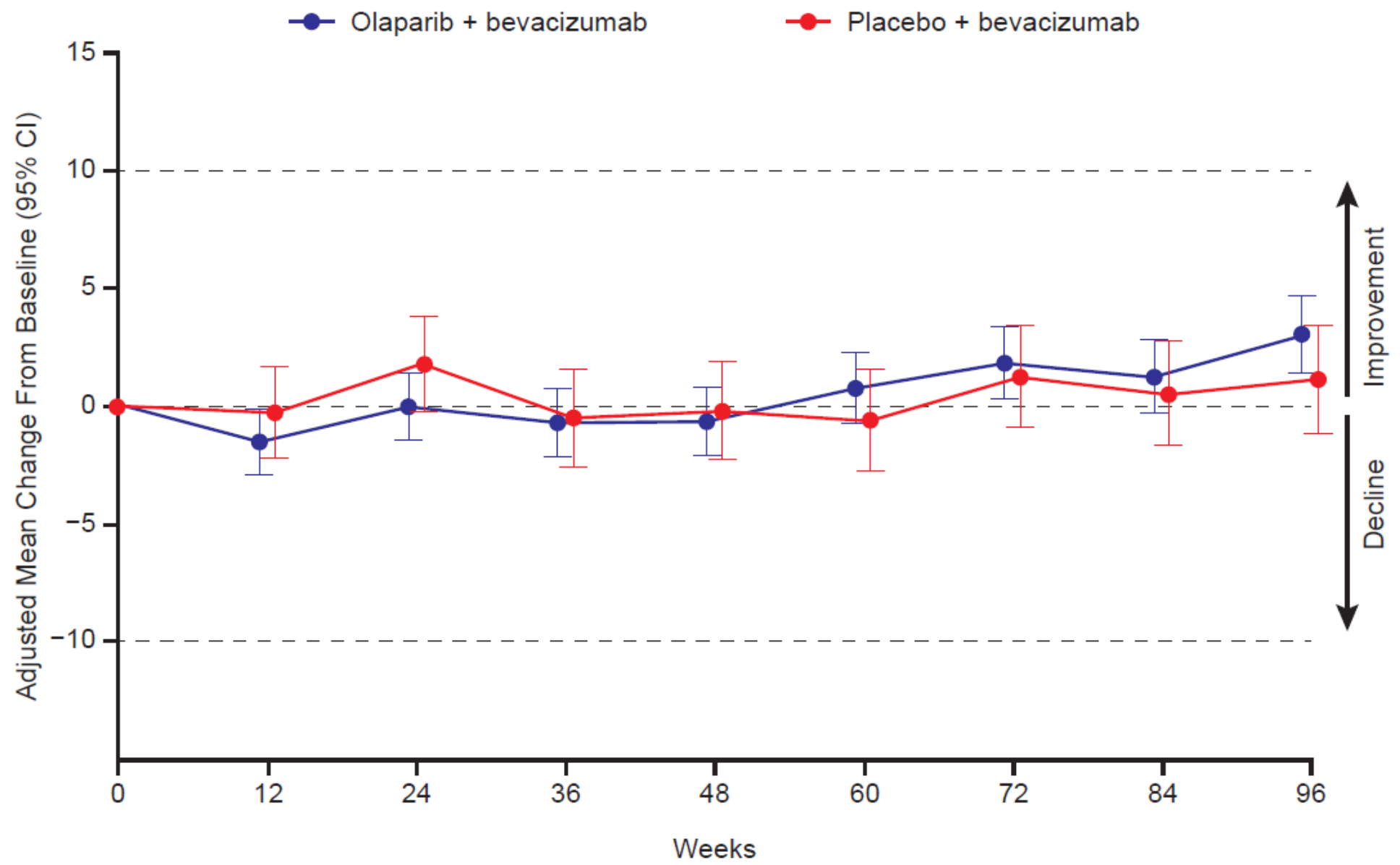

No. of patients:									
Olaparib + bevacizumab	513	460	438	398	399	357	342	315	291
Placebo + bevacizumab	252	229	211	202	189	174	171	158	142

B Role functioning

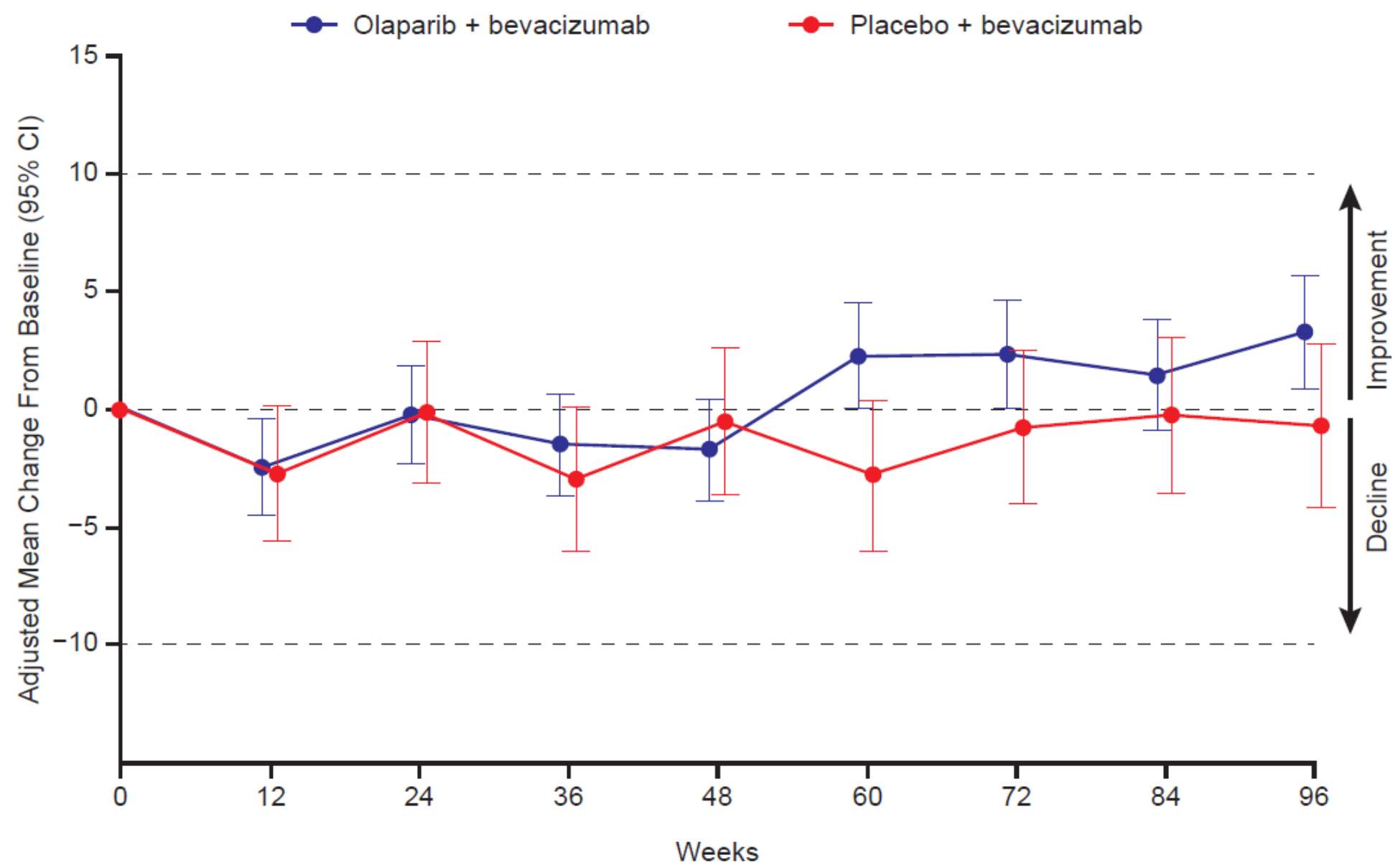

No. of patients:									
Olaparib + bevacizumab	514	460	441	401	399	359	345	316	292
Placebo + bevacizumab	252	231	211	202	188	174	172	158	142

C Emotional functioning

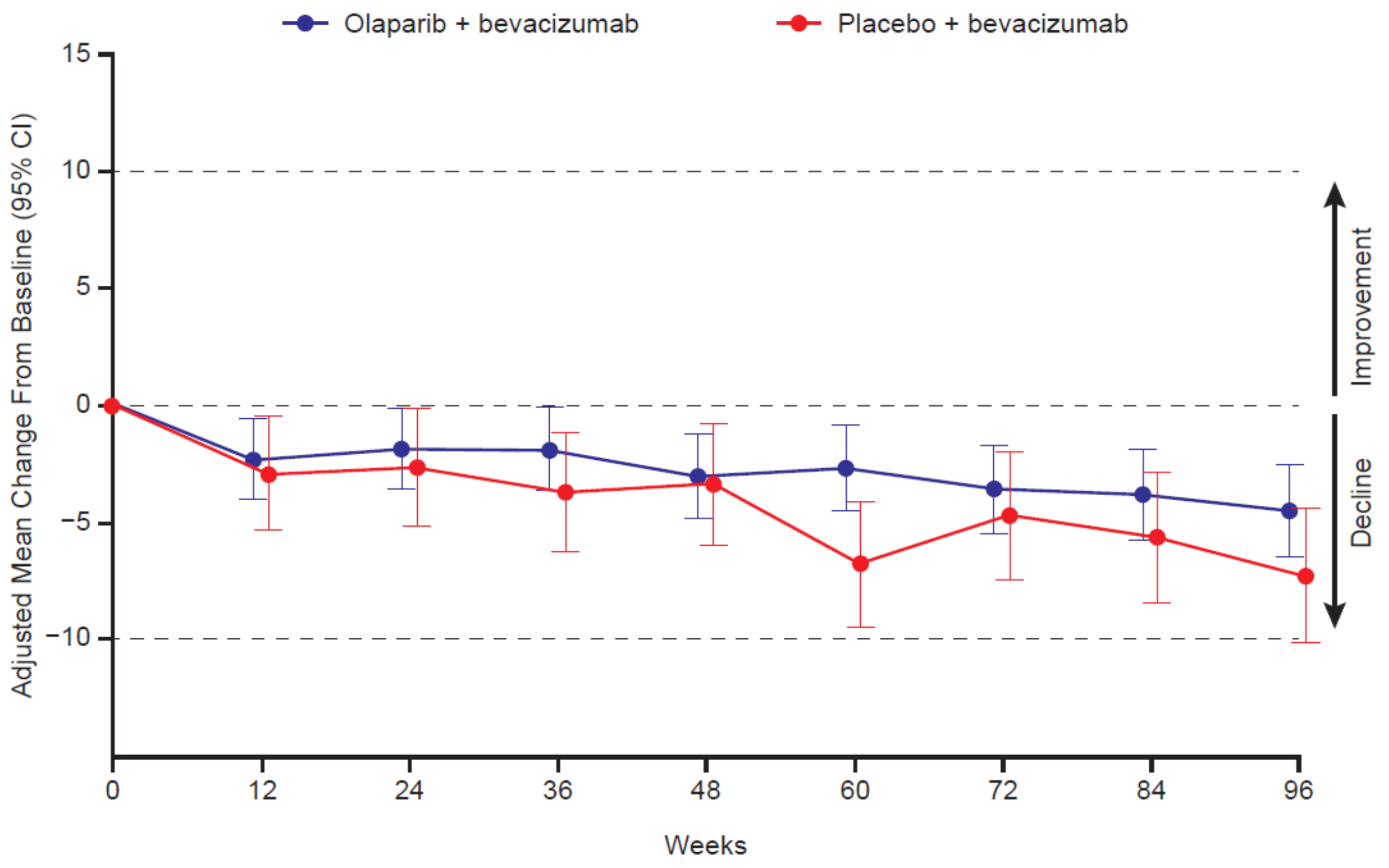

No. of patients:

Olaparib + bevacizumab	515	463	441	405	397	360	344	312	293
Placebo + bevacizumab	251	229	209	201	187	173	167	156	140

D Cognitive functioning

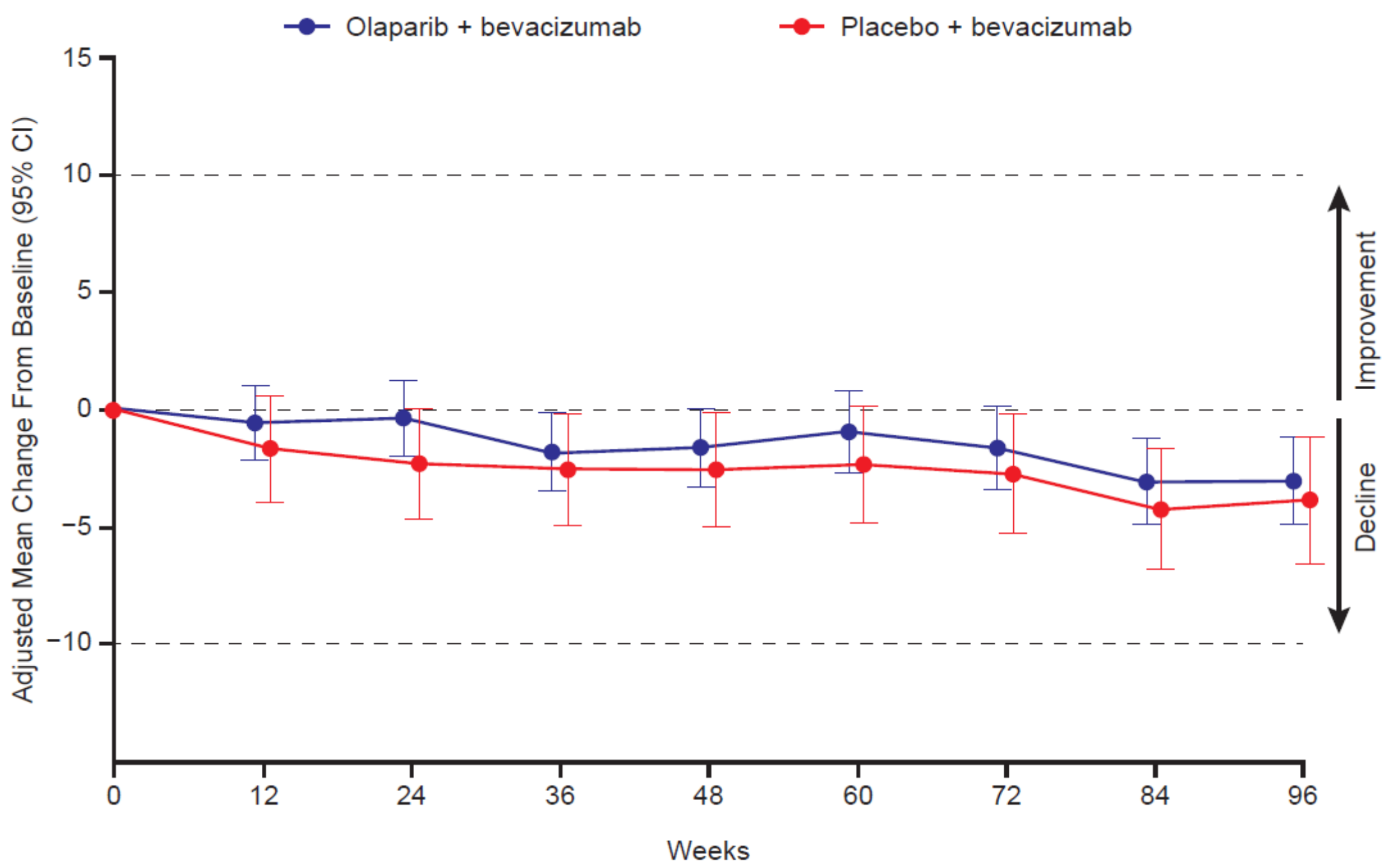

No. of patients:

Olaparib + bevacizumab	515	463	441	405	398	360	345	313	293
Placebo + bevacizumab	251	229	209	201	187	173	167	156	140

E Social functioning

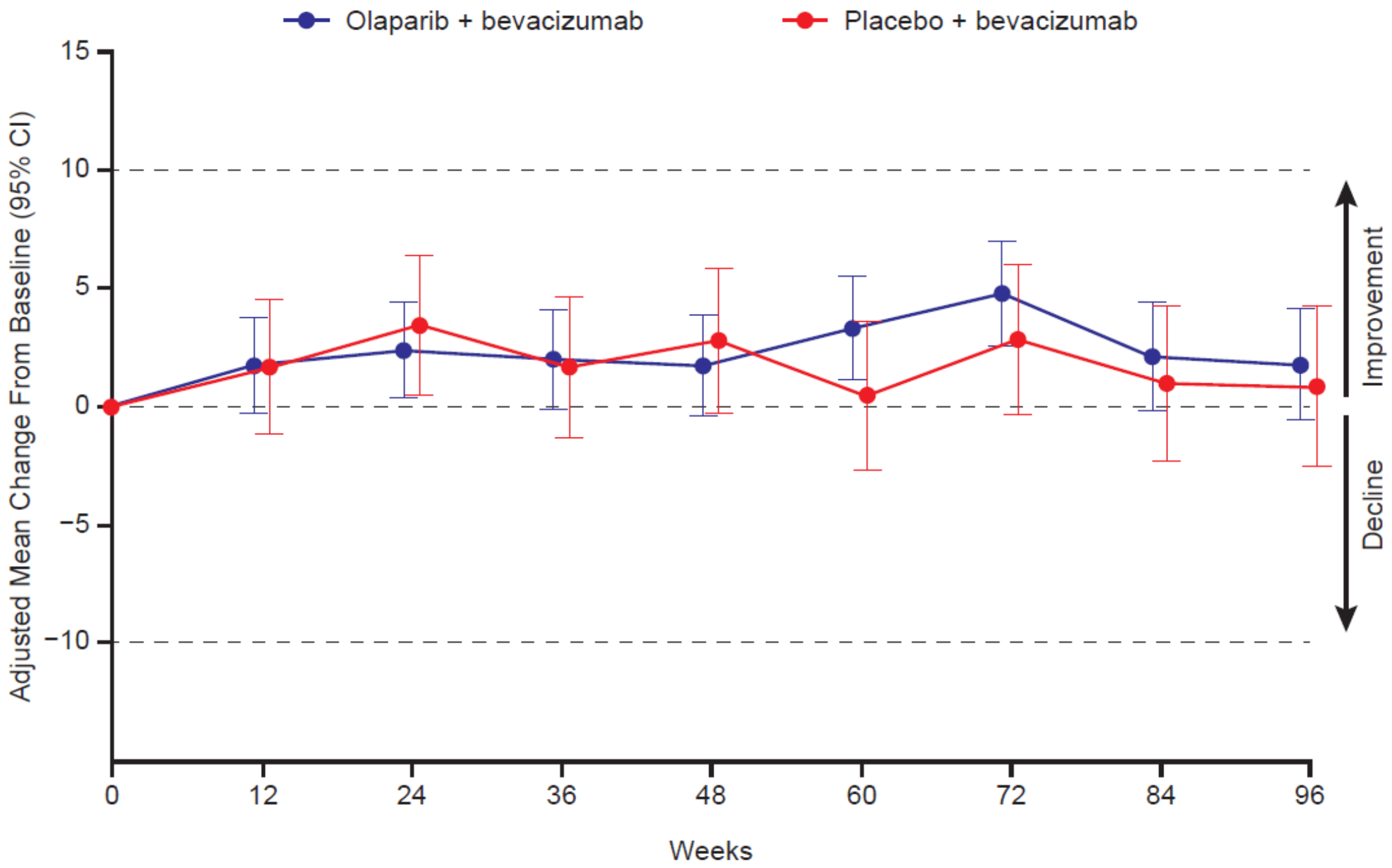

No. of patients:									
Olaparib + bevacizumab	515	461	441	405	397	360	344	314	293
Placebo + bevacizumab	251	229	209	201	187	173	167	156	139

Adjusted mean change using mixed model for repeated measures at each follow-up timepoint; error bars represent 95% CI. Horizontal dashed lines represent 10-point MID. For functional scales, higher scores represent higher levels of function.

CI = confidence interval; EORTC QLQ-C30 = European Organisation for Research and Treatment of Cancer Core Quality of Life Questionnaire; ITT = intention-to-treat; MID = minimal important difference.

Figure S3. Adjusted mean change over time in EORTC QLQ-C30 scores for (**A**) pain, (**B**) dyspnea, and (**C**) diarrhea in the modified ITT population

A Pain

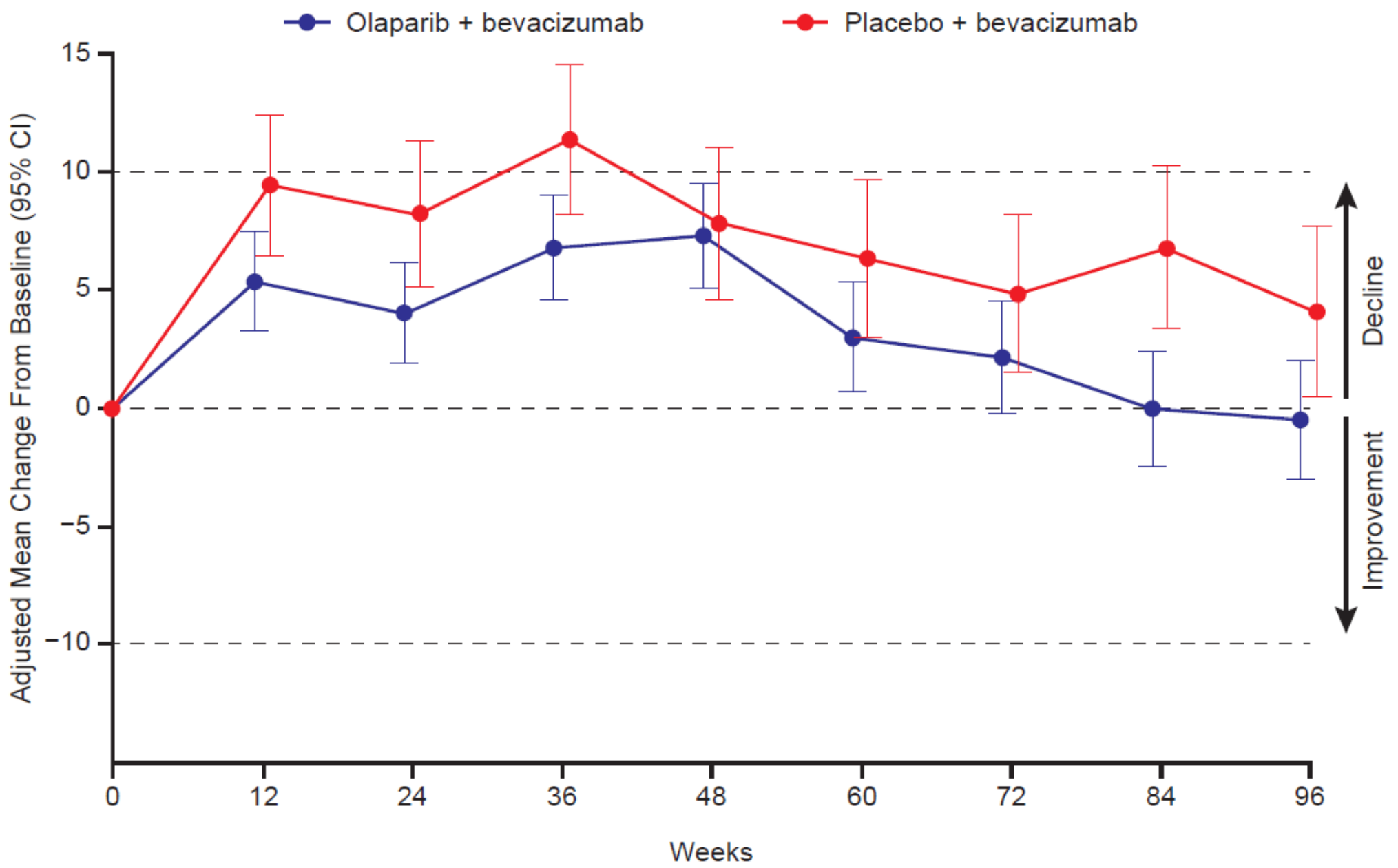

No. of patients:									
Olaparib + bevacizumab	516	464	441	404	401	363	347	317	294
Placebo + bevacizumab	253	232	211	203	189	174	172	158	142

B Dyspnea

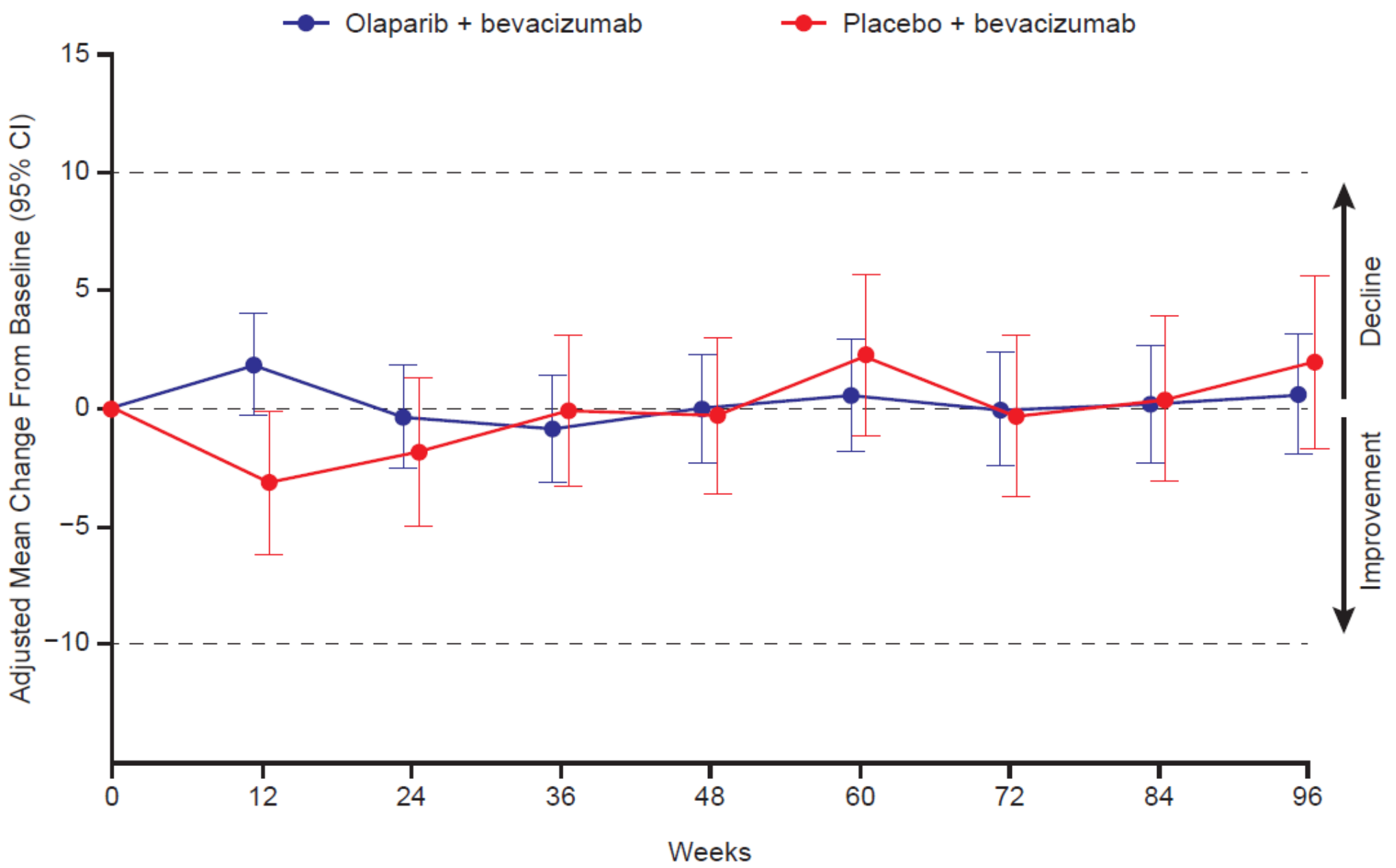

No. of patients:									
Olaparib + bevacizumab	509	454	433	390	396	355	340	311	289
Placebo + bevacizumab	252	231	210	202	187	173	171	158	141

C Diarrhea

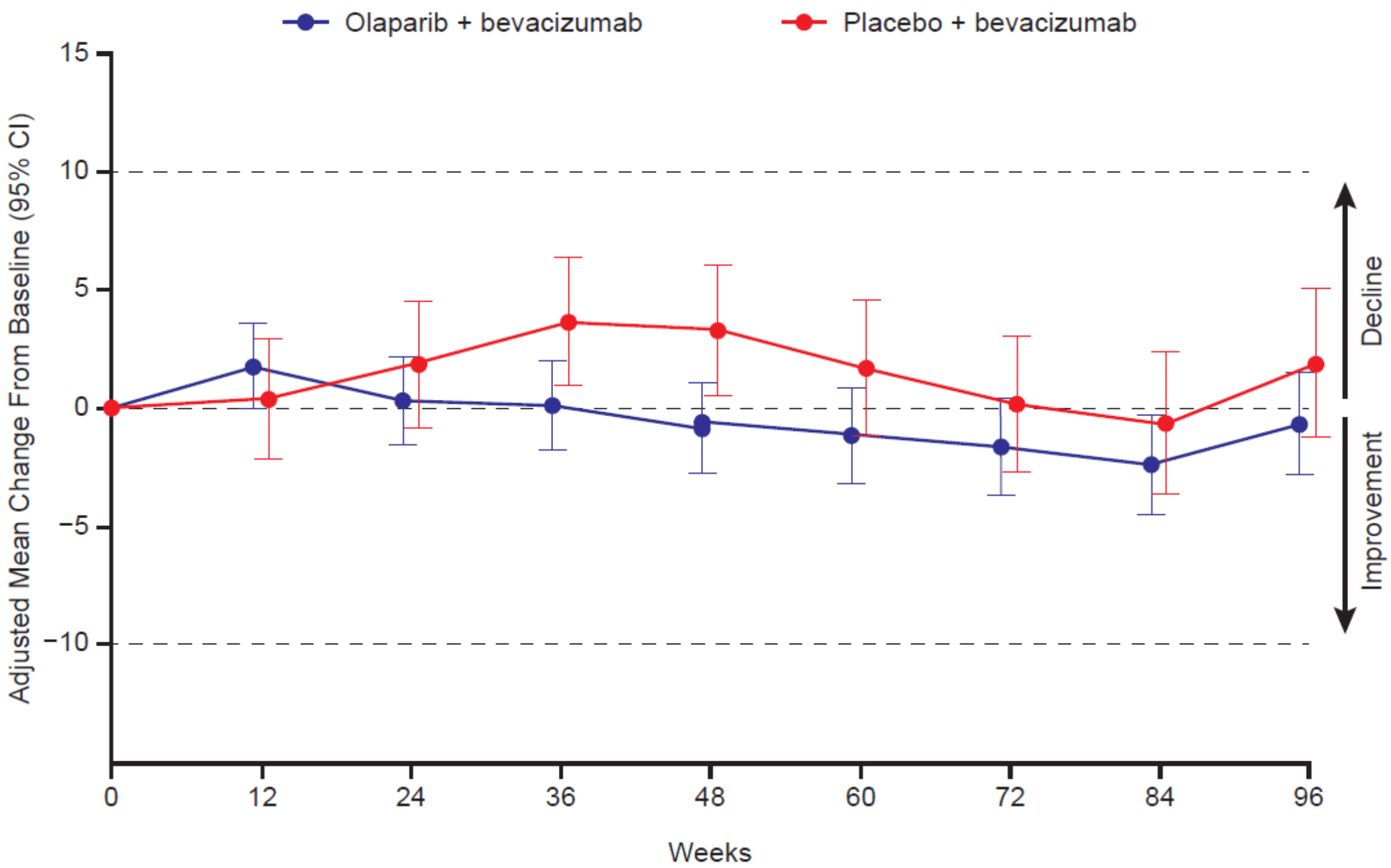

No. of patients:									
Olaparib + bevacizumab	510	457	435	398	393	353	337	307	287
Placebo + bevacizumab	252	230	208	199	188	173	169	154	141

Adjusted mean change using mixed model for repeated measures at each follow-up timepoint; error bars represent 95% CI. The horizontal dashed lines shown on the graphs are used to represent the 10-point MID cutoff. For symptomatic scales, higher scores for represent higher symptomatic burden.

CI = confidence interval; EORTC QLQ-C30 = European Organisation for Research and Treatment of Cancer Core Quality of Life Questionnaire; ITT = intention-to-treat; MID = minimal important difference.

Figure S4. Adjusted mean change over time in EORTC QLQ-OV28 scores for (**A**) body image, (**B**) Sexual function, (**C**) attitude to disease/treatment, (**D**) abdominal/GI symptoms, (**E**) peripheral neuropathy, (**F**) hormonal/menopause symptoms, (**G**) other chemotherapy side effects, and (**H**) hair loss in the modified ITT population

A Body image

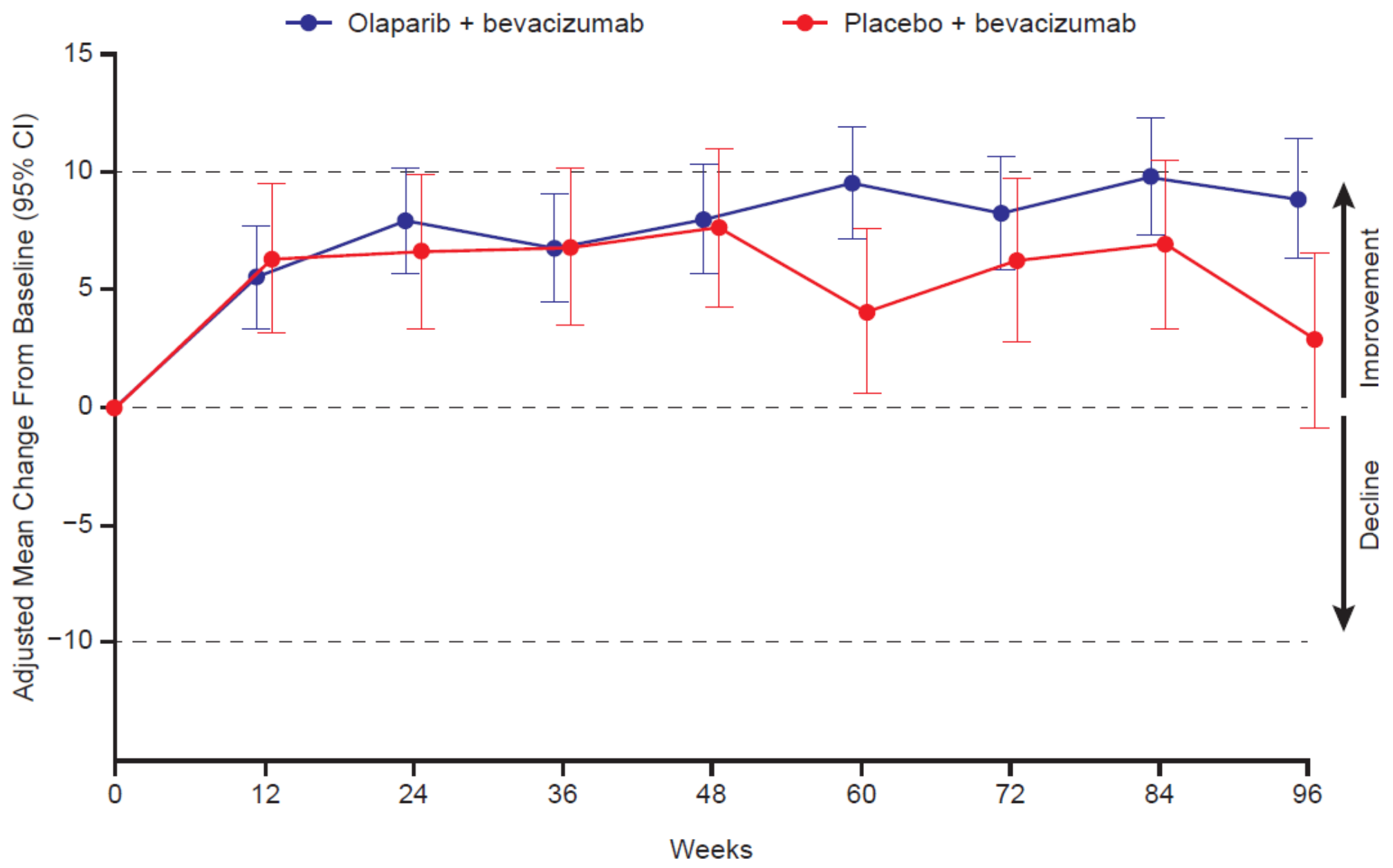

No. of patients:									
Olaparib + bevacizumab	514	458	433	398	393	356	339	312	289
Placebo + bevacizumab	245	221	205	191	183	168	167	152	137

B Sexual function

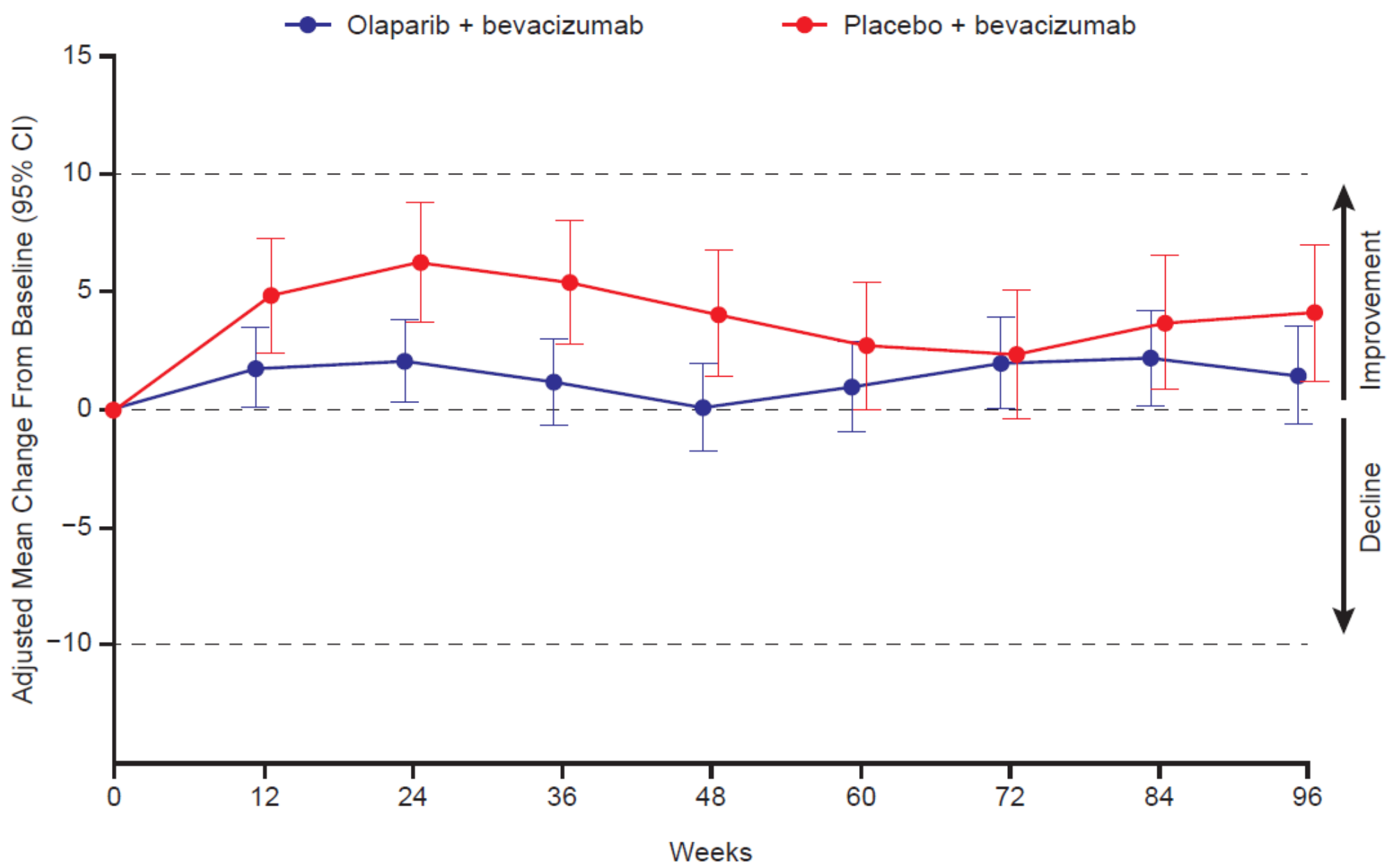

No. of patients:									
Olaparib + bevacizumab	489	420	385	347	342	307	296	268	253
Placebo + bevacizumab	238	210	188	173	165	154	149	136	126

C Attitude to disease/treatment

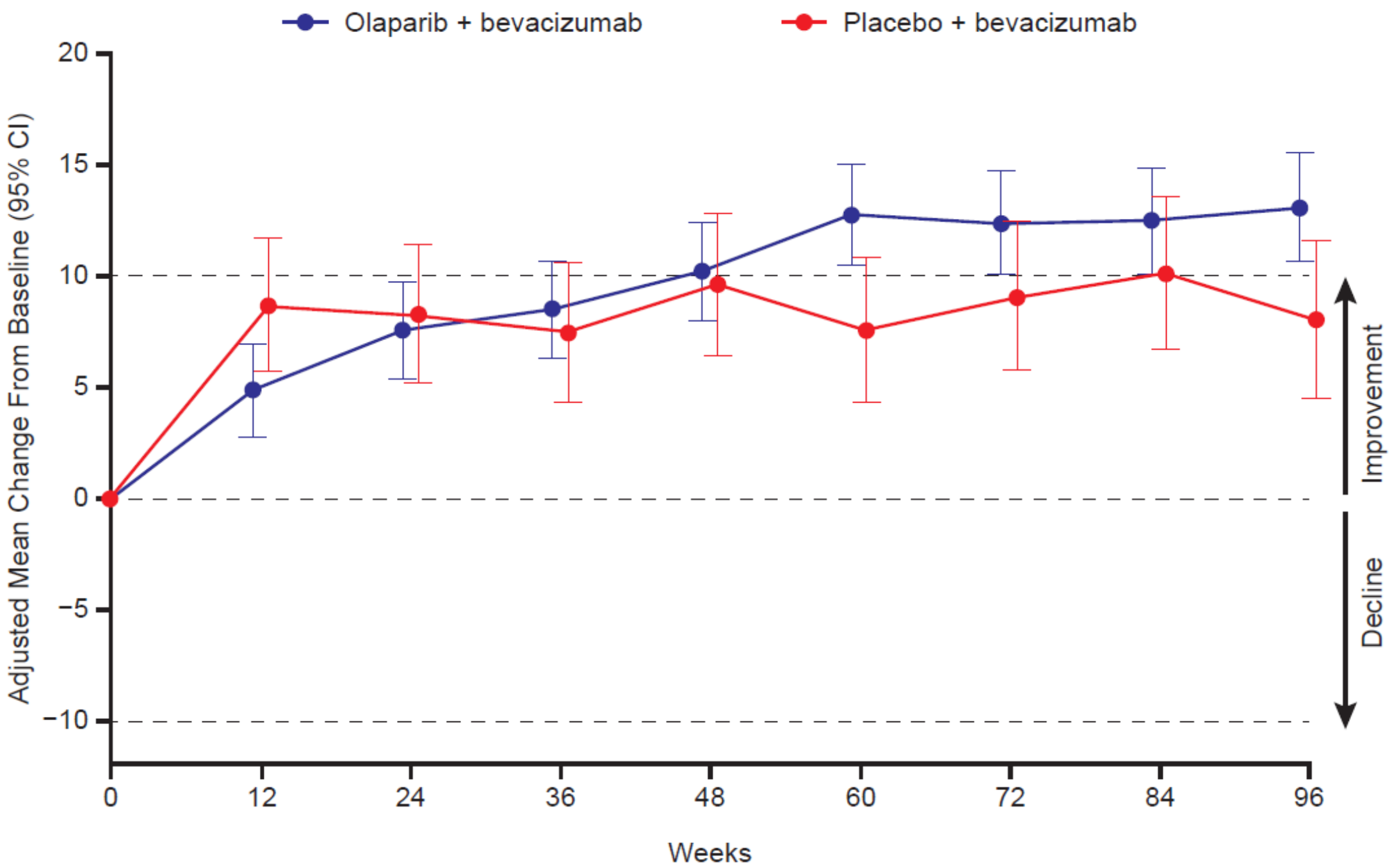

No. of patients:									
Olaparib + bevacizumab	511	454	429	396	390	357	336	310	287
Placebo + bevacizumab	246	220	205	191	181	167	167	151	135

D Abdominal/GI symptoms

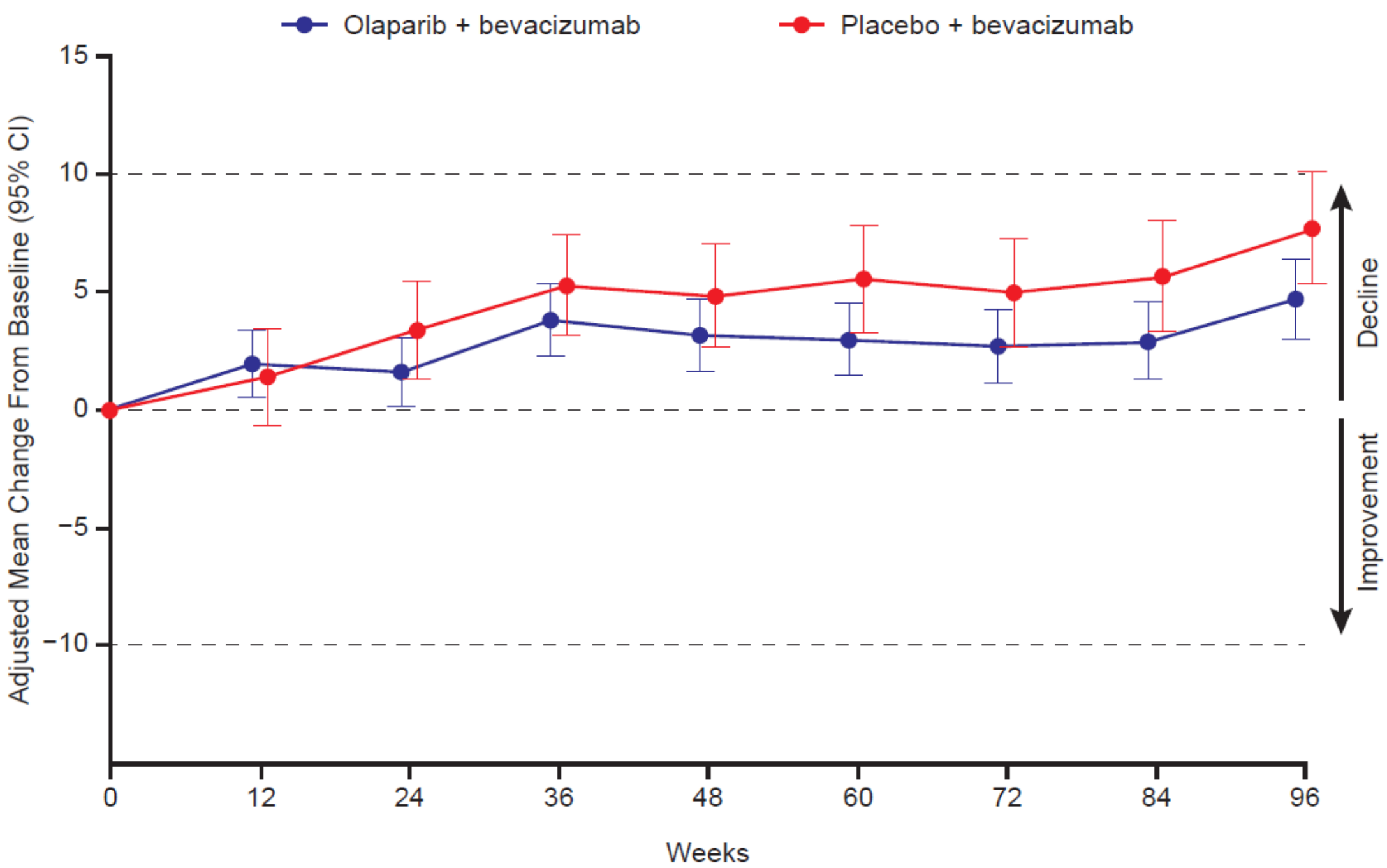

No. of patients:									
Olaparib + bevacizumab	517	465	439	400	400	360	345	318	292
Placebo + bevacizumab	247	223	208	198	186	171	166	155	141

E Peripheral neuropathy

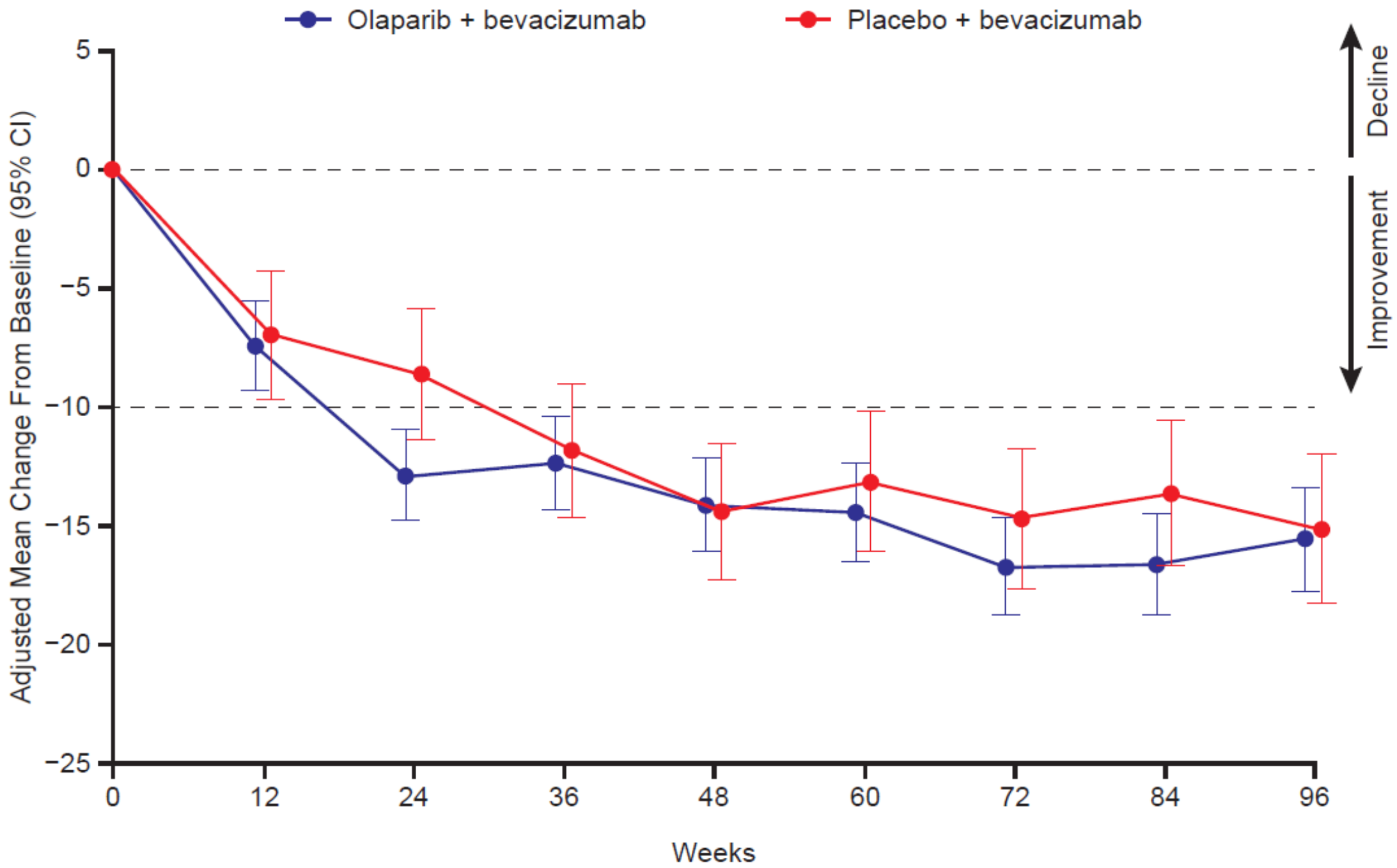

No. of patients:									
Olaparib + bevacizumab	517	465	434	396	399	357	345	317	288
Placebo + bevacizumab	246	221	207	196	184	170	166	154	141

F Hormonal/menopause symptoms

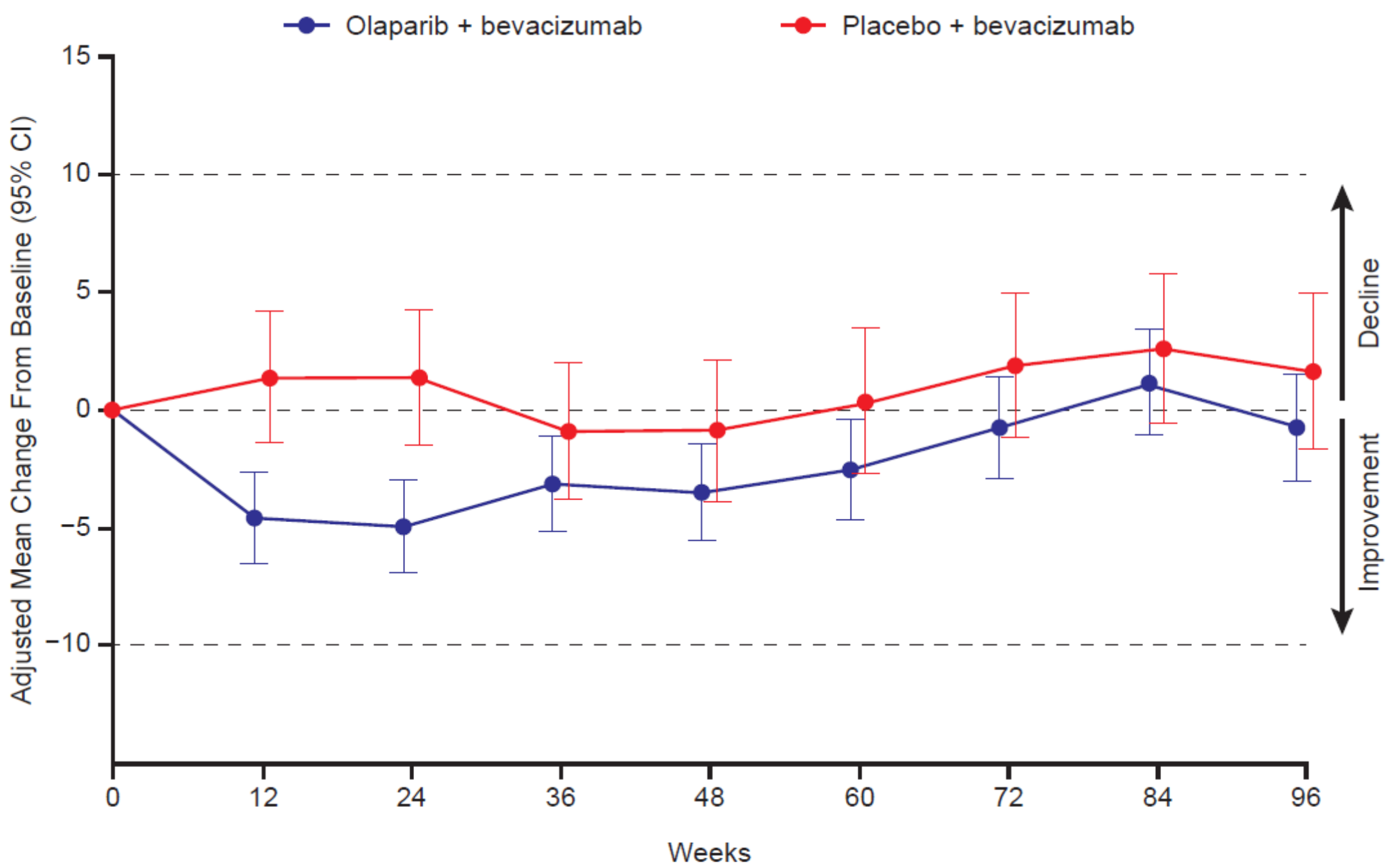

No. of patients:									
Olaparib + bevacizumab	516	463	435	398	398	361	345	315	291
Placebo + bevacizumab	245	220	206	197	184	170	168	154	139

G Other chemotherapy side effects

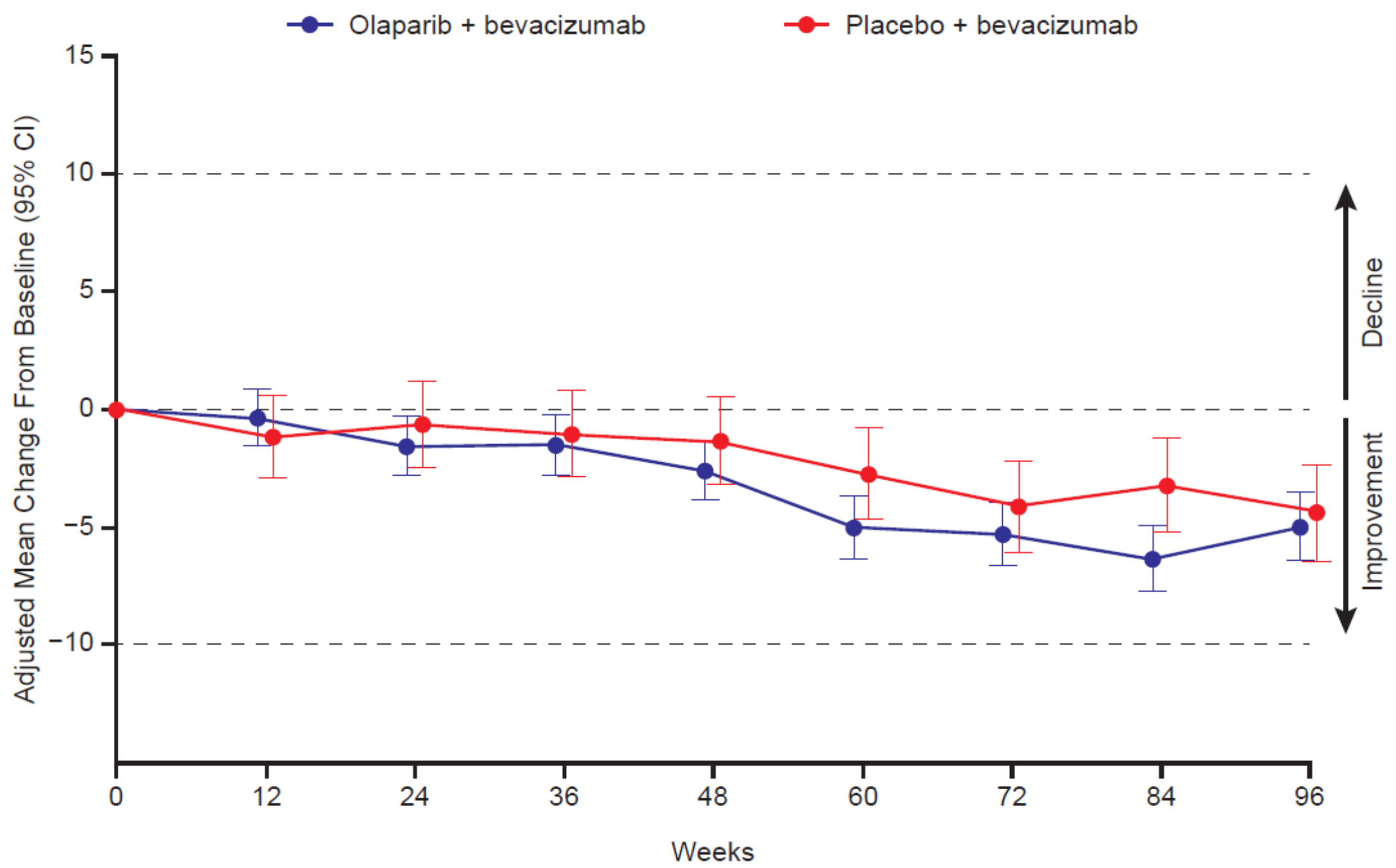

No. of patients:									
Olaparib + bevacizumab	517	465	438	396	399	357	345	315	289
Placebo + bevacizumab	246	221	207	196	184	170	166	154	141

H Hair loss

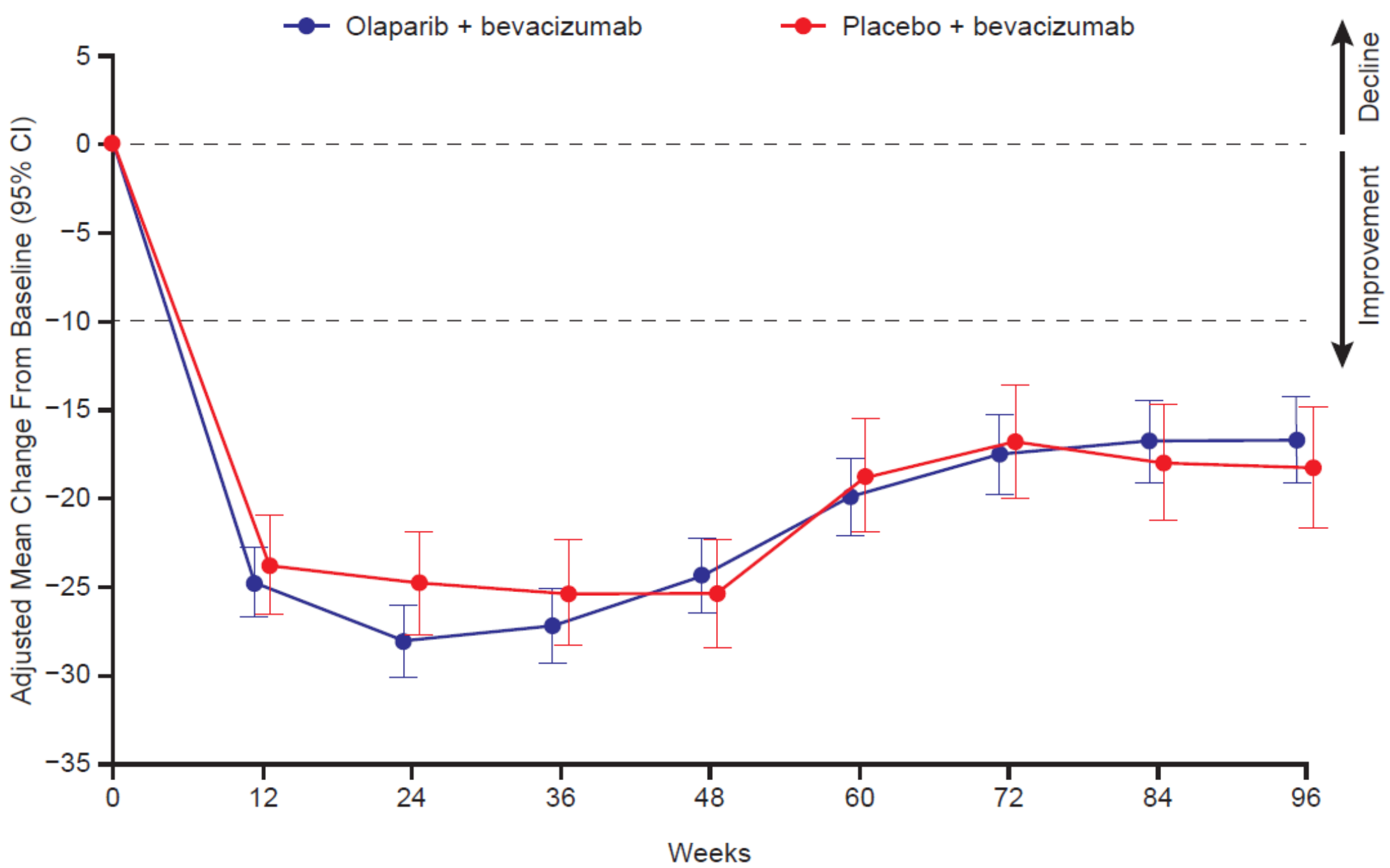

No. of patients:									
Olaparib + bevacizumab	502	446	418	384	386	342	333	303	277
Placebo + bevacizumab	244	219	203	194	183	166	163	152	138

Adjusted mean change using mixed model for repeated measures at each follow-up timepoint; error bars represent 95% CI. Horizontal dashed lines represent the 10-point MID. Higher scores for functional scales represent higher functional level and higher scores for symptomatic scales represent higher symptomatic burden.

CI = confidence interval; EORTC QLQ-OV28 = Quality of Life Questionnaire Ovarian cancer module; GI = gastrointestinal; ITT = intention-to-treat; MID = minimal important difference.

Figure S5. Between-group difference in adjusted mean global change from baseline for (**A**) functional scales and (**B**) symptomatic scales of the EORTC QLQ-C30 by HRD status[a]

A

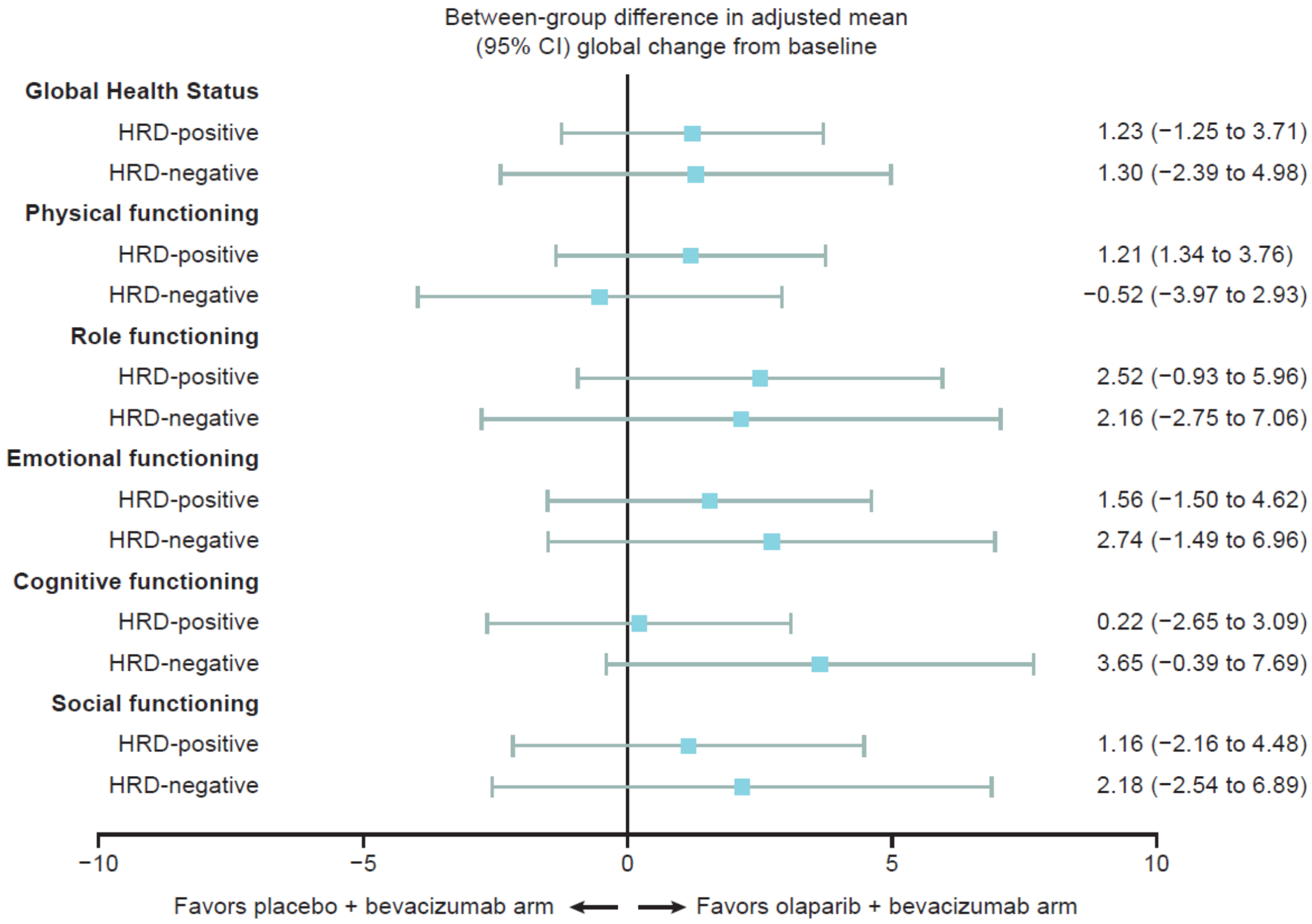

B

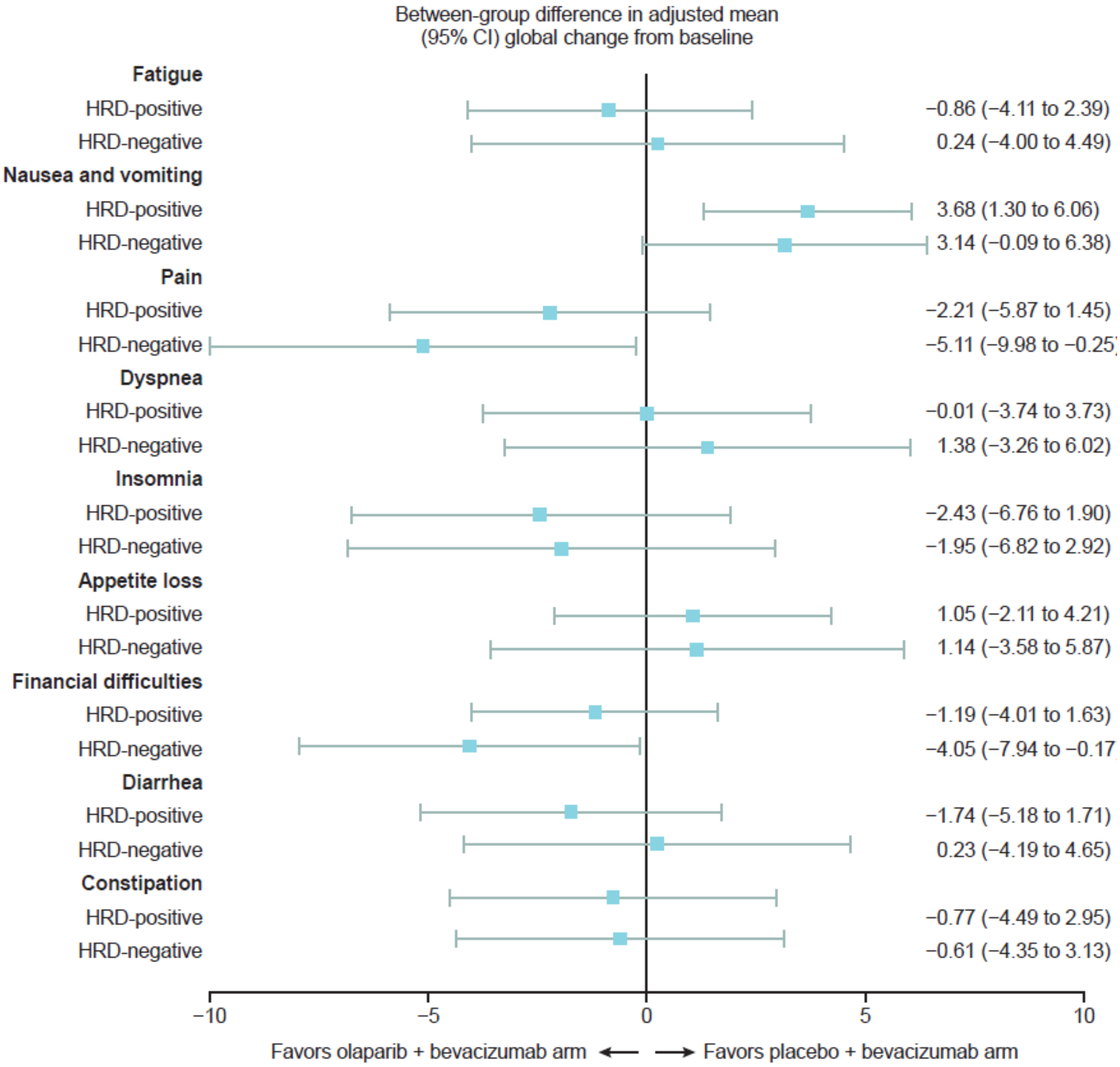

Error bars represent 95% CI.

[a]Mixed model for repeated measures analysis.

CI = confidence interval; EORTC QLQ-C30 = European Organisation for Research and Treatment of Cancer Core Quality of Life Questionnaire; HRD = homologous recombination deficiency.

Figure S6. Between-group difference in adjusted mean global change from baseline for (**A**) functional scales and (**B**) symptomatic scales of the EORTC QLQ-OV28 by HRD status[a]

A

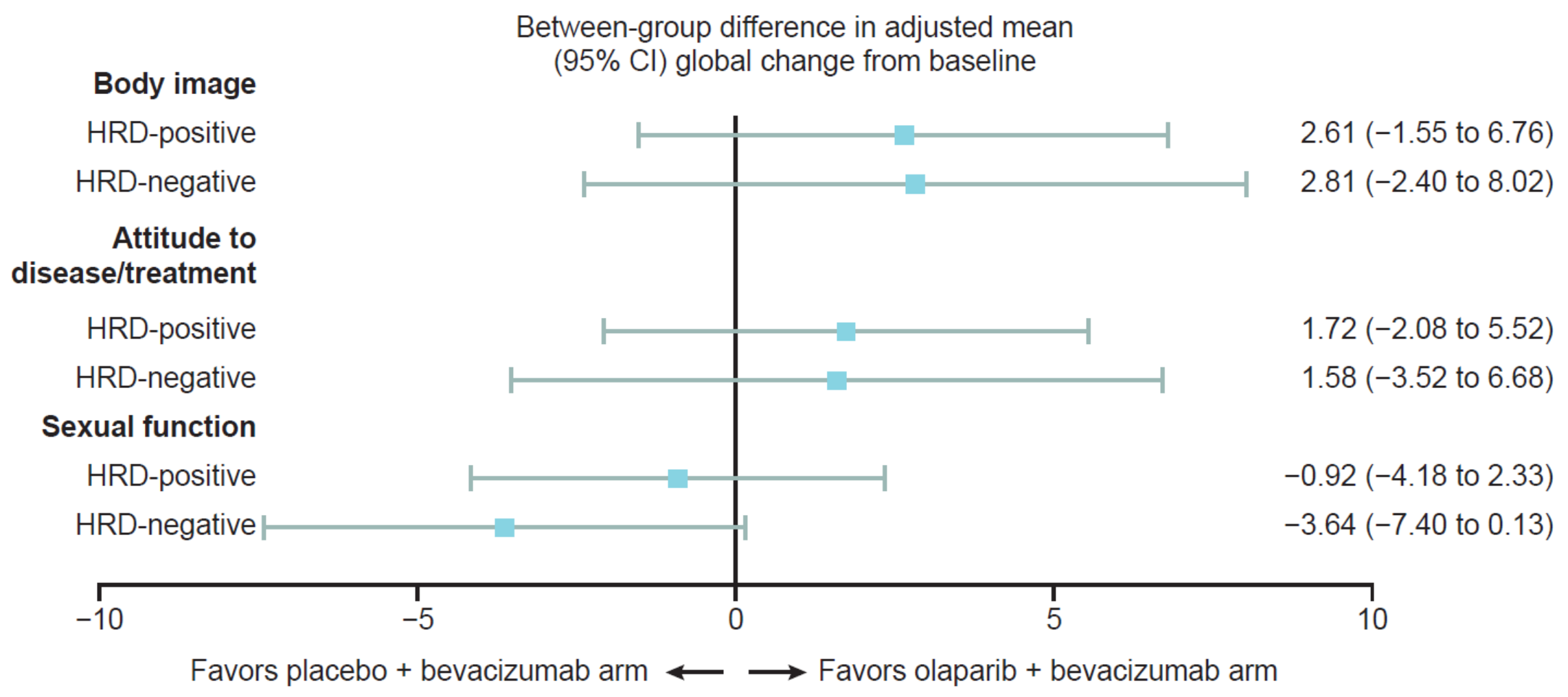

B

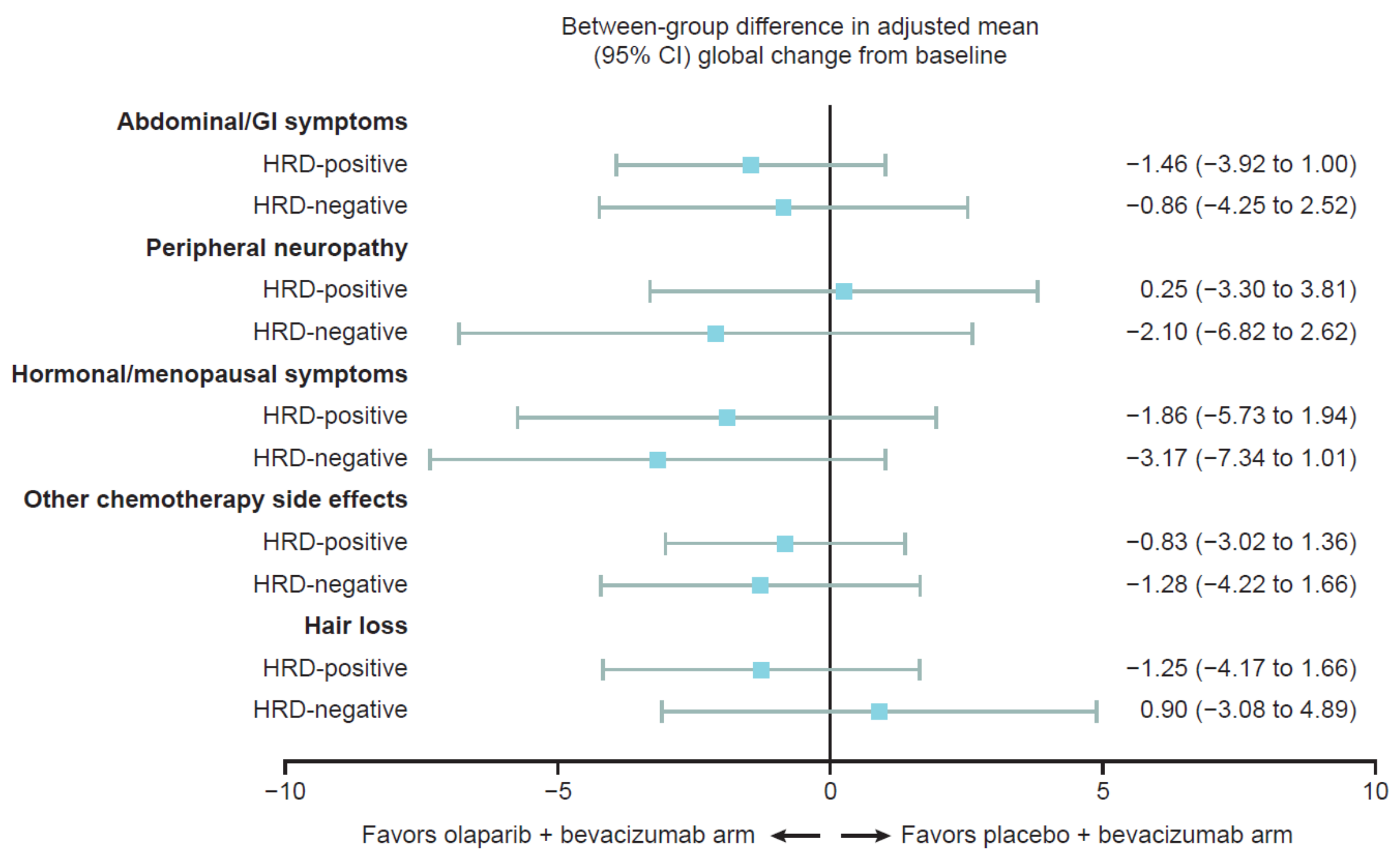

Error bars represent 95% CI.

[a]Mixed model for repeated measures analysis.

CI = confidence interval; EORTC QLQ-OV28 = European Organisation for Research and Treatment of Cancer Quality of Life Questionnaire Ovarian Cancer module; GI = gastrointestinal; HRD = homologous recombination deficiency.

Figure S7. TWiST by HRD status (HRD-positive and HRD-negative/unknown subgroups) for TOX defined as (**A**) all grade ≥2 AEs with symptoms, (**B**) grade ≥2 AEs with symptoms associated with olaparib (fatigue/asthenia, nausea, or vomiting), and (**C**) all grade ≥2 AEs.

A All grade ≥2 AEs with symptoms

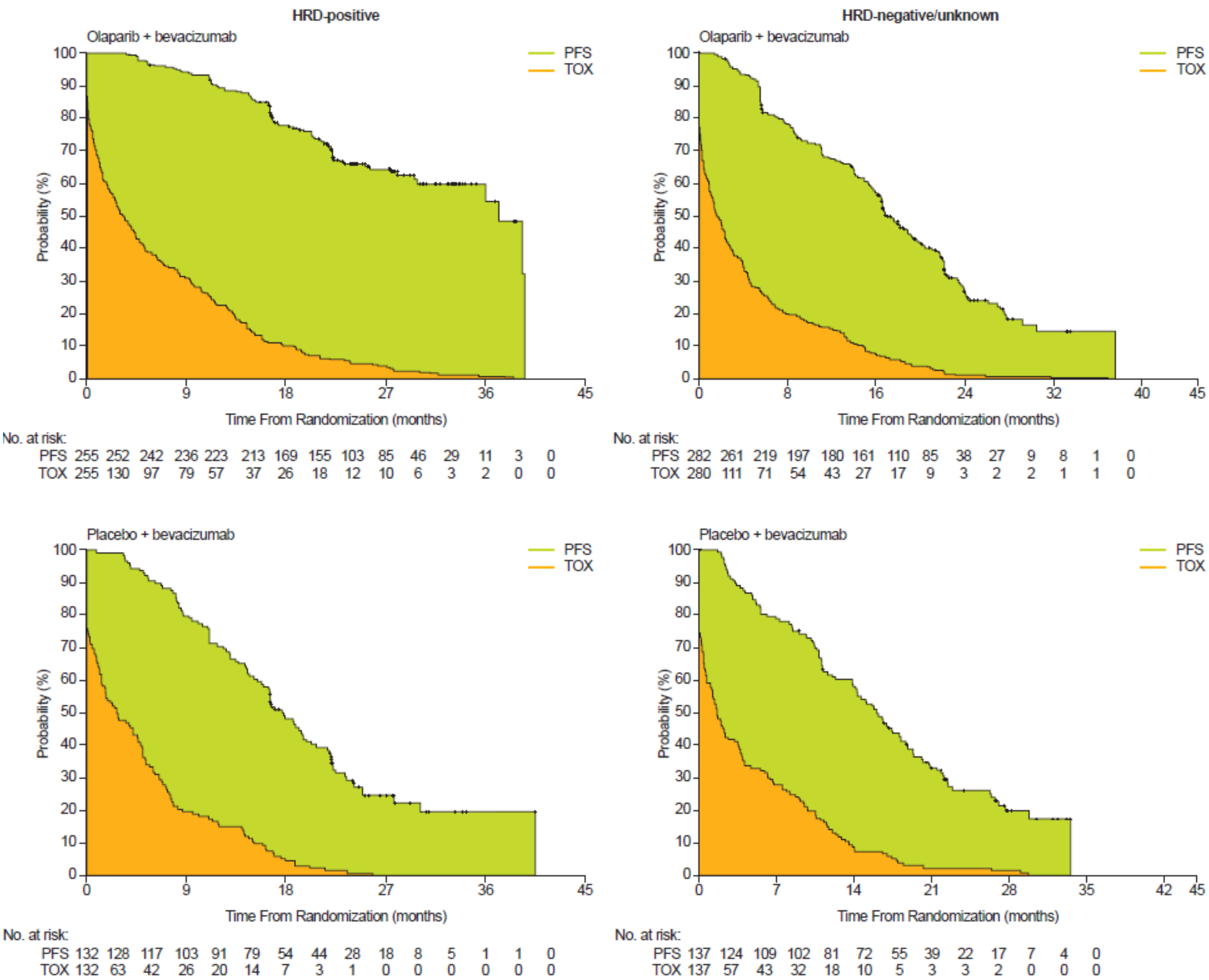

	Olaparib + bevacizumab	Placebo + bevacizumab
Median TWiST, months (95% CI)	36.6 (22.4 to NE)	12.6 (9.8 to 15.6)
P value	<.0001	

	Olaparib + bevacizumab	Placebo + bevacizumab
Median TWiST, months (95% CI)	11.5 (10.0 to 14.9)	10.5 (8.5 to 14.0)
P value	.3772	

B Grade ≥2 AEs with symptoms associated with olaparib

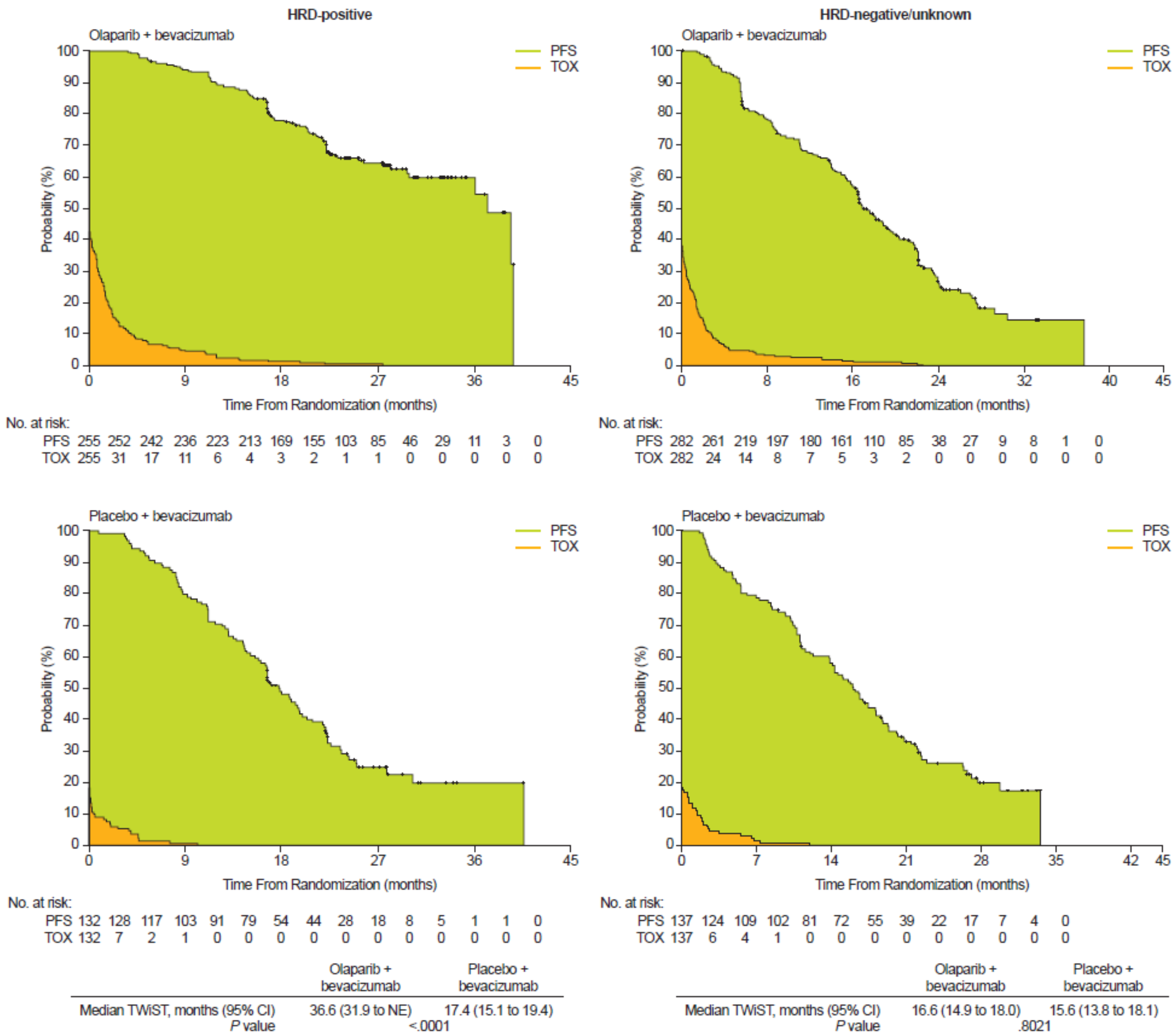

C All grade ≥2 AEs

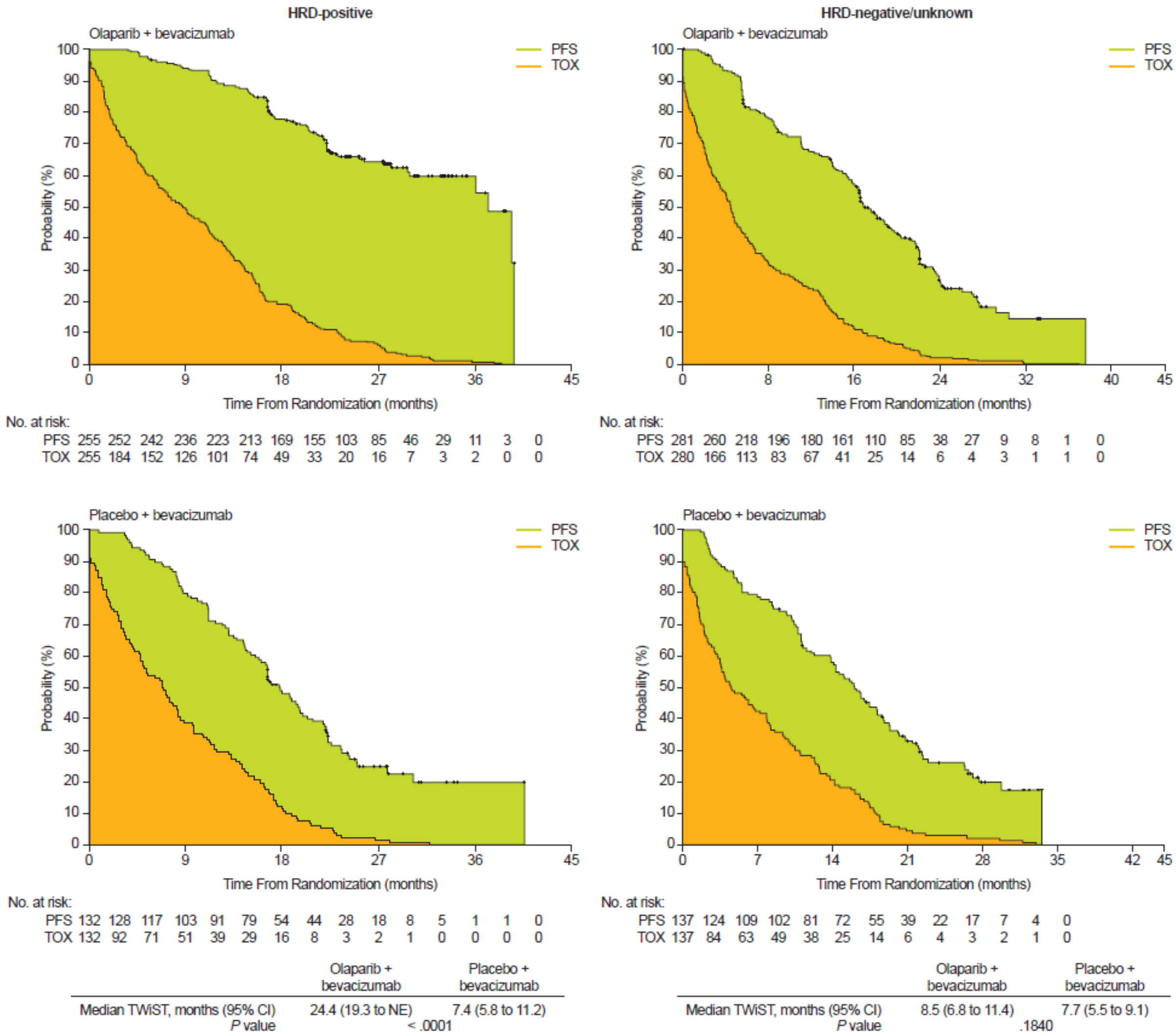

DCO March 22, 2019.

AE = adverse event; CI = confidence interval; DCO = data cutoff; HRD = homologous recombination deficiency; NE = not estimable; PFS = progression-free survival; TOX = toxicity; TWiST = time without symptoms or toxicity.
